# Hybrid *de novo* genome assembly of Chinese chestnut (*Castanea mollissima*)

**DOI:** 10.1093/gigascience/giz112

**Published:** 2019-09-12

**Authors:** Yu Xing, Yang Liu, Qing Zhang, Xinghua Nie, Yamin Sun, Zhiyong Zhang, Huchen Li, Kefeng Fang, Guangpeng Wang, Hongwen Huang, Ton Bisseling, Qingqin Cao, Ling Qin

**Affiliations:** 1 Beijing Advanced Innovation Center for Tree Breeding by Molecular Design, Beijing University of Agriculture, 7 Beinong Rd., Beijing 102206, China; 2 College of Plant Science and Technology, Beijing Key Laboratory for Agricultural Application and New Technique, Beijing University of Agriculture, 7 Beinong Rd., Beijing 102206, China; 3 Research Center for Functional Genomics and Biochip, 23 Hongda St., Tianjin 300457, China; 4 Laboratory of Molecular Biology, Department of Plant Sciences, Wageningen University, Droevendaalsesteeg 1, Wageningen 6708 PB, The Netherlands; 5 College of Landscape Architecture, Beijing Collaborative Innovation Center for Eco-Environmental Improvement with Forestry and Fruit Trees, Beijing University of Agriculture, 7 Beinong Rd., Beijing 102206, China; 6 Changli Institute of Pomology, Hebei Academy of Agriculture and Forestry Sciences, 39 E Jieyangdajie, Changli 066600, China; 7 South China Botanical Garden, Chinese Academy of Sciences, 723 Xingke Rd., Guangzhou 510650, China

**Keywords:** *Castanea mollissima*, genome assembly, annotation, evolution

## Abstract

**Background:**

The Chinese chestnut (*Castanea mollissima*) is widely cultivated in China for nut production. This plant also plays an important ecological role in afforestation and ecosystem services. To facilitate and expand the use of *C. mollissima* for breeding and its genetic improvement, we report here the whole-genome sequence of *C. mollissima*.

**Findings:**

We produced a high-quality assembly of the *C. mollissima* genome using Pacific Biosciences single-molecule sequencing. The final draft genome is ∼785.53 Mb long, with a contig N50 size of 944 kb, and we further annotated 36,479 protein-coding genes in the genome. Phylogenetic analysis showed that *C. mollissima* diverged from *Quercus robur*, a member of the Fagaceae family, ∼13.62 million years ago.

**Conclusions:**

The high-quality whole-genome assembly of *C. mollissima* will be a valuable resource for further genetic improvement and breeding for disease resistance and nut quality.

## Data Description

### Background information

*Castanea*, a genus of the Fagaceae family, occurs naturally throughout the forests of eastern North America, Europe, and Asia, where it is ecologically and economically important. *Castanea* contains 7 species. Chinese chestnut (*Castanea mollissima*), Chinese seguin (*Castanea seguinii*), Chinese chinkapin (*Castanea henryi*), and Japanese chestnut (*Castanea crenata*) occur in East Asia and show high genetic diversity [1]. The American chestnut (*Castanea dentata*) and chinkapin (*Castanea pumila*) occur only in North America, while the European chestnut (*Castanea sativa*) is distributed in Europe, and they are the predominant tree species in the deciduous forests of eastern North America and some parts of Northern Italy and Southern France [2]. Chestnuts are important forest resources that provide wood products and food, and they are also keystone species due to their ecological roles in afforestation and ecosystem services [3].

The Chinese chestnut is geographically widespread and is cultivated in 26 Chinese provinces for commercial nut production [4], and the country is rich in diverse germplasm resources. Cultivation of Chinese chestnut has a long history, which spans >6,000 years, according to archeological discoveries in the Banpo Ruins of Xi'an, China [5]. The annual nut yield of Chinese chestnut is high. In 2017, Chinese chestnut production was 1,939,719 tonnes, accounting for 83.34% of the world's total chestnut production that year [6]. Owing to its high nut quality, easily peeled pellicle, excellent adaptability to infertile soil, and natural resistance to diseases, Chinese chestnut has been broadly used in breeding programs in the United States, especially to introduce resistance to the fungal pathogen chestnut blight (*Cryphonectria parasitica*) [7, 8]. An accidental introduction of the chestnut blight fungus at the beginning of the 20^th^ century destroyed 4 billion American chestnuts, which were a predominant forest tree species by 1950 [9–11]. Three quantitative trait loci (QTLs) of resistance to blight disease were identified in the F_2_ mapping population of an interspecies cross of *C. mollissima* × *C. dentata* and 2 of them shared synteny with 2 QTLs for powdery mildew resistance in peach [12, 13]. Recently, 2 QTLs were also identified for resistance to *Phytophthora cinnamomi* in the population of *C. sativa* × *C. crenata* and the QTL located in linkage group E is in line with a previous preliminary study on a segregating population of a cross between *C. mollissima* and *C. dentata* [14]. Chinese chestnut has substantial levels of resistance to chestnut blight, and the first QTL analyses show that it is a good resource to introduce resistance into American chestnut [7].

A chestnut genome sequence project was launched within the Fagaceae Genomic Tools because of the economic and ecological importance of this tree species. This has resulted in a genome sequence using data obtained with a Roche 454 platform and Sanger sequencing data (V1.1). This genome sequence was released in 2014 at the Hardwood Genomics website [15]. Recently, an updated version of this Chinese chestnut genome was made available online on bioRxiv [16]. The assembly quality of these 2 genome sequences is compared in Table S1. The updated version showed improved assembly quality compared with the previous versions in some parameters, such as contig length range, counts of contig sequences, and maximum length of contigs; however, a high-quality annotated whole-genome sequence for Chinese chestnut is still urgently needed. This is essential for molecular studies on major traits involved in nut quality and disease resistance [17–19]. In the present study, we report a high-quality whole-genome sequence of *C. mollissima*. This genome sequence will facilitate studies on the evolution of *Castanea* including comparative genomics and processes underlying domestication. Furthermore, it will support breeding programs leading to genetic improvement of chestnuts.

### Sampling and sequencing

**Figure 1:**
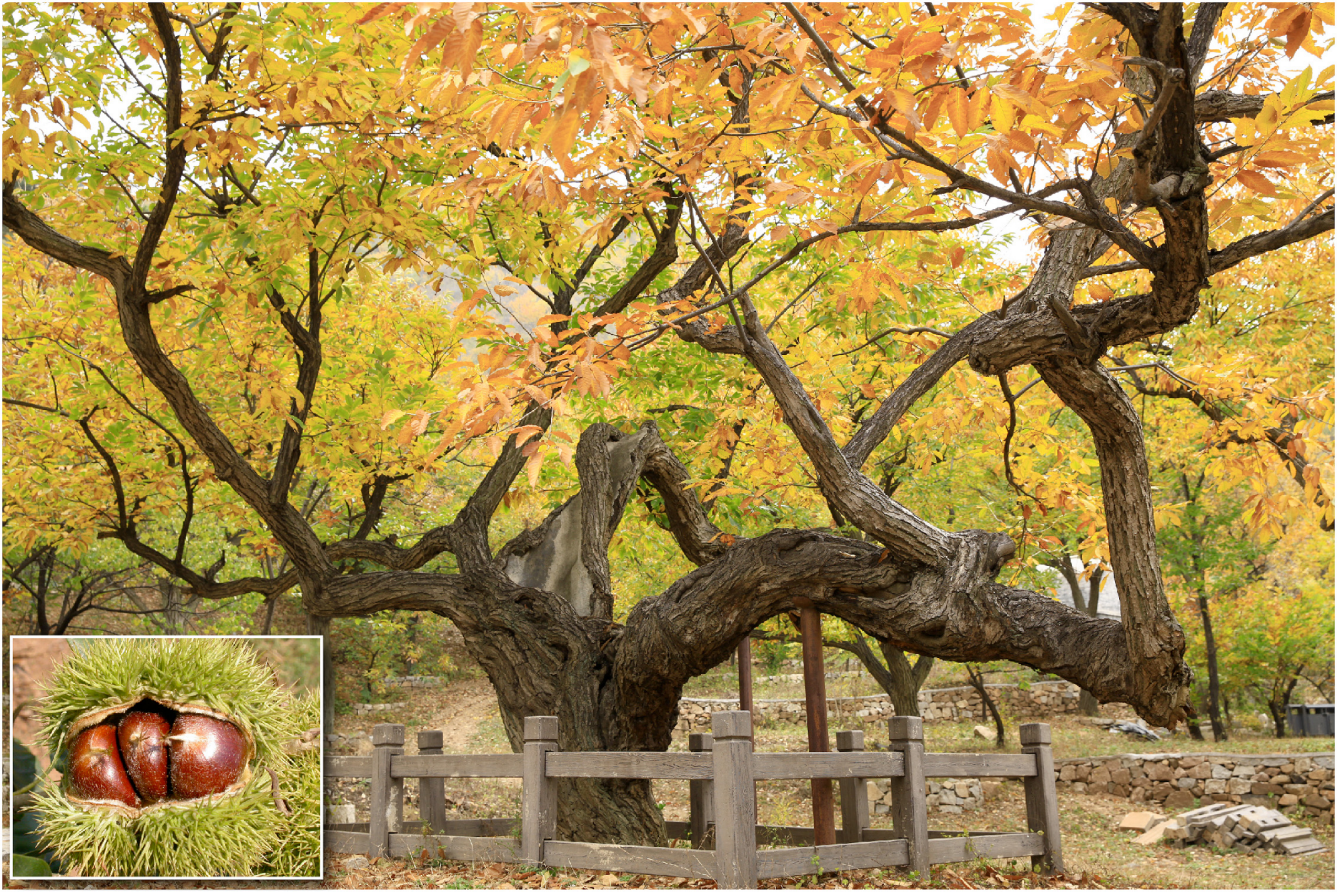
Example of Chinese chestnut tree (*C. mollissima*). Natural habitat of *C. mollissima* (image from the Water Great Wall, Beijing, China) and the nut of *C. mollissima* (image from Ling Qin) are shown.

A mature, healthy tree of wild *C. mollissima* was chosen from the Zhangcunping national forest reserve (31.2803 N, 111.1403 E, 1,261 m altitude) of the city of Yichang in Hubei Province, China. The individual measured ∼12 m in height, and its trunk was ∼10 cm in diameter at breast height. Fresh leaves were collected on 18 June 2017. The samples were immediately frozen in liquid nitrogen and then stored at -80 °C. The genomic DNA of *C. mollissima* was extracted using the DNeasy Plant Mini Kit (Qiagen, Hilden, Germany) and used for sequencing (Fig. 1). The DNA was sheared by a Covaris S2 system (Covaris, USA) for short-insert paired-end (PE) library construction. The shearing conditions were as follows: the number of cycles was 2, and the shearing time was 40 seconds per cycle. Short-insert libraries with a size of 500 bp were constructed according to the instructions in the Illumina Library Preparation Kit (Illumina, San DiegoCA, USA). All libraries were sequenced on an Illumina HiSeq 2500 sequencer with the PE 2 × 150 bp protocol. The raw data were filtered and trimmed. Illumina data quality control settings were as follows: SLIDINGWINDOW: 4: 15 MINLEN: 50 using Trimmomatic software. In total, ∼34 Gb of clean data were generated, yielding a sequencing depth of ∼42.7×. For PacBio library construction, the genomic DNA of *C. mollissima* was sheared to 20 kb, and fragments shorter than 7 kb were filtered using BluePippin (Sage Science, Beverly, MA, USA). The filtered DNA was then used to prepare a proprietary SMRTbell library using the PacBio DNA Template Preparation Kit (Pacific Biosciences, Menlo Park, CA, USA). The PacBio data quality control standard of RQ > 0.75 was used, and the minimum subread length was 500 bp using SMRT Link 6.0 software. In total, ∼69 Gb of quality-filtered data were obtained from PacBio sequencing, with a average read length of 7,170 bp and a sequencing depth of ∼87× (Table S2).

### Genome size and heterozygosity estimation

The distribution of short subsequence (*k*-mer) frequency, also known as the *k*-mer spectrum, is widely used to estimate genome size [20, 21]. A *k*-mer depth distribution was obtained from a Jellyfish [22] analysis, and the peak depth was clearly observed from the distribution data. The genome size was calculated with the following formula: genome size = total_k-mer_num/k-mer_depth (total_k-mer_num is the total number of *k*-mers from all reads, and k-mer_depth is the peak depth). Based on this method, the size of the *C. mollissima* genome was estimated to be ∼772 Mb, and the heterozygosity level of *C. mollissima* was ∼0.87% (Fig. S1). Comparing this estimate with those for beech and oak, we found that our result sample was more similar to European beech (Table S3) [23, 24].

### Genome assembly and annotation

All of the subreads from PacBio sequencing were assembled using SMARTdenovo software with default values for all parameters except for -J, which was set to a value of 4,000 (-J 4000 filters all reads with lengths <4,000 bp) [25]. The assembled sequence was then polished using Quiver (SMRT Analysis version 2.3.0) with the default parameters. To achieve a high-accuracy genome assembly, 6 rounds of iterative error correction were performed using the clean Illumina data. In total, 785.53 Mb of final assembly was obtained after correction using PacBio and Illumina PE read sequences, and the assembly comprised 2,707 contigs (N50 = 944 kb, N90 = 133 kb) (Table 1). Both RepeatModeler and RepeatMasker (RepeatMasker, RRID:SCR_012954) [26] were used for the *de novo* identification and masking of repeats. To ensure the integrity of genes in the subsequent analyses, low-complexity regions or simple repeats were not masked because some of these sequences could be within genes. Finally, 49.69% of the assembled bases were masked (Table S4). Protein-coding region identification and gene prediction were performed through a combination of *ab initio*, homology-based, and transcriptome-based prediction methods. The *ab initio* gene prediction was conducted with Augustus (Augustus, RRID:SCR_008417; version 3.2.2), GeneMark-ET (version 4.29), and SNAP15 to predict coding genes. For the homology-based prediction, homologous proteins from several species (*Vitis vinifera, Prunus persica, Populus trichocarpa, Oryza sativa, Medicago truncatula, Glycine max, Citrus clementina, Theobroma cacao, Pyrus bretschneideri*) were downloaded from NCBI and aligned to the assembled genome. Then, Exonerate (Exonerate, RRID:SCR_016088; version 2.47.3) [27] was used to generate gene structures based on the homology alignments. For the transcriptome-based prediction, transcriptome data were generated from mixed samples of flowers, buds, leaves, nuts, and roots on the Illumina HiSeq 2500 platform (a total of 20.84 Gb raw data) and mapped to the genome assembly using TopHat (TopHat, RRID:SCR_013035; version 2.1.1). Cufflinks (Cufflinks, RRID:SCR_014597; version 2.1.1) [28] was then used to identify spliced transcripts in the gene models. All the gene evidence predicted by the aforementioned 3 approaches was integrated by EVidenceModeler (EVM version 1.1.1). Finally, a total of 36,479 protein-coding gene models were constructed (Table 1).

**Table 1:** Summary of *C. mollissima* genome assembly and gene model

Genome assembly statistics	Value
Total length	785,529,252 bp
No. of contigs	2,707
Largest contig length	6,584,328 bp
N50 length (contigs)	944,461 bp
N90 length (contigs)	133,678 bp
Counts of N50 (contigs)	235
Counts of N90 (contigs)	1,024
**Gene model statistics**	
Gene number	36,479
Gene density (per 100 kb)	4.64
Gene mean length	1,139.63 bp
Exon number per gene	4.41
Exon mean length	258.15 bp
Intron mean length	1,156.91 bp
Genome GC content	36.07%
Exon GC content	43.36%

GC: guanine-cytosine.

The obtained gene set was functionally analyzed using BLASTP (BLASTP, RRID:SCR_001010) with an E-value of 1e^-5^ against the NCBI-NR, Swiss-Prot, and euKaryotic Orthologous Groups (KOG) databases. Protein domains were annotated by mapping genes to the InterPro and Pfam databases using InterProScan (InterProScan, RRID:SCR_005829) [29] and HMMER (Hmmer, RRID:SCR_005305) [30]. Potential gene pathways were derived via gene mapping against the KEGG databases, and Gene Ontology (GO) terms were extracted from the corresponding InterProScan or Pfam results (Fig. S2).

### Quality assessment

To evaluate the completeness and coverage of the assembly, we aligned Illumina DNA and RNA reads to the *C. mollissima* assembly using BWA (BWA, RRID:SCR_010910) [31] and HISAT [32], respectively. The percentages of aligned DNA and RNA reads were 95.46% and 97.41%, respectively. In the core gene estimation using BUSCO (BUSCO, RRID:SCR_015008) [33], 1,392 of the 1,440 core genes (96.7%) were found to be complete in the assembled genome, and 1,412 (complete BUSCOs and fragmented BUSCOs) (98.1%) of the 1,440 core genes had at least partial matches (Table S5). This result indicates that the assembly contains almost all genic regions, which further confirms the high quality of the *C. mollissima* genome assembly.

### Physical map alignment

A total of 19,064 bacterial artificial chromosome (BAC) double-ended sequences from the previously published physical map [34] were aligned with the genome sequenced in the present study. Of these, 17,999 of the sequences were mapped onto our genome, accounting for 94.41% of all BAC double-ended sequences. The reason that 1,065 (5.59%) of the sequences did not map to the genome is most likely due to individual differences. The results also showed that 1,184 of 1,300 contigs from the physical map could be mapped onto our genome (Table S6).

### Gene family expansion and contraction

**Figure 2:**
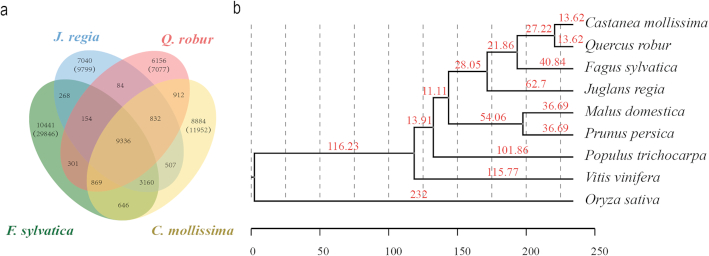
Phylogenetic relationships between Chinese chestnut and other species. A maximum-likelihood tree was obtained with 540 single-copy orthologous genes. (a) The shared and unique gene families in 4 closely related species are shown in the Venn diagram. Each number represents a number of gene families, and the number in parentheses is a number of genes. (b) The estimated divergence times are displayed on the phylogenetic tree.

To understand the relationships of the *C. mollissima* gene families with those of other plants, we performed a systematic comparison of genes among different species. The protein-coding genes of 9 genomes, namely, *O. sativa* [35], *Malus domestica* [36], *P. trichocarpa* [37], *P. persica* [38], *C. mollissima, Quercus robur* [39], *Fagus sylvatica* [24], *Juglans regia* [40], and *V. vinifera* [41], were used for the comparison. Gene loss and gain are among the primary reasons for functional changes. To gain greater insights into the evolutionary dynamics of the genes, we determined the expansion and contraction of the orthologous gene clusters in these 8 species with CAFE software (CAFÉ, RRID:SCR_005983) [42]. In the Chinese chestnut genome, a total of 17,422 gene families were identified, while 27,502 families of homologous genes were detected across the 9 species. Of all the gene families (17,422), 209 were significantly expanded and 89 were contracted (*P* < 0.05) in *C. mollissima* (Fig. S3). The Venn diagram in Fig. 2a shows that 9,336 gene families were shared by the 4 species *C. mollissima, Q. robur, J. regia*, and *F. sylvatica*. In addition, both specific and common gene families were detected in these 4 species. A total of 11,952 genes and 8,884 gene families were found to be specific to Chinese chestnut (Table S7).

### Phylogenetic analysis

To examine the evolutionary relationships of Chinese chestnut with other plants, we applied RAxML software (RAxML, RRID:SCR_006086; version 8.0.0; substitution model PROTGAMMAJTT, bootstrap value 100) [43] to perform a maximum-likelihood genome-wide phylogenetic analysis of 540 single-copy genes from the 9 plant genomes (Fig. 2b). The results support the hypothesis that Chinese chestnut and oak are sister groups. On the basis of the phylogeny and fossil record [5], we estimated the divergence time. The phylogenetic tree indicates that the orders Fagales and Rosales have a close genetic relationship, with a divergence time of 90.75 million years ago (Mya). The estimated divergence time of *C. mollissima* and *Q. robur* in the Fagales clade is ∼13.62 Mya, while that of Chinese chestnut and *J. regia* is 62.7 Mya.

### Long terminal repeat insertion

**Figure 3:**
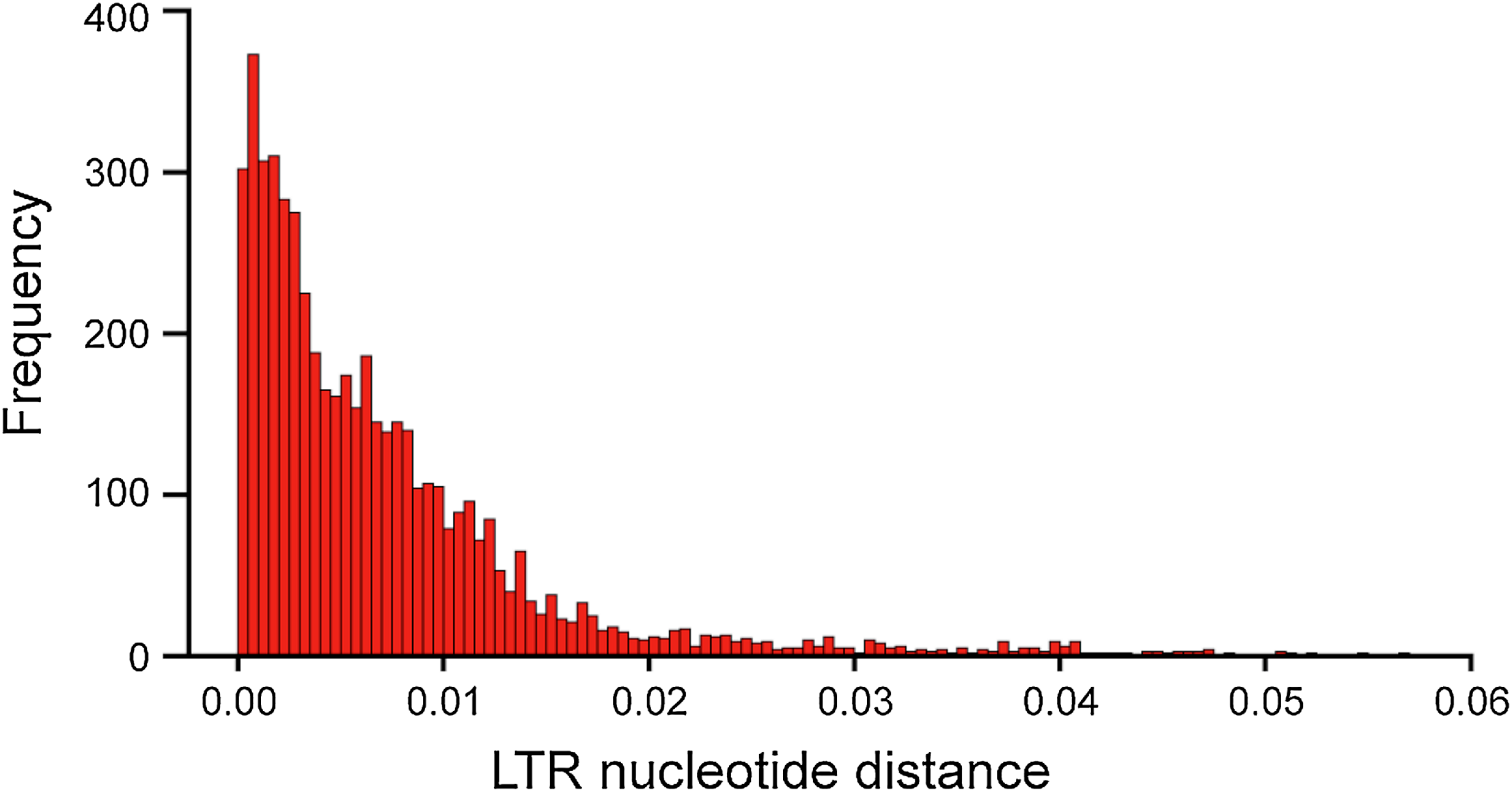
Nucleotide distance distribution of annotated LTR elements in *C. mollissima*.

In the final assembly, ∼390 Mb of repetitive sequence was found, accounting for 49.69% of the genome. Long terminal repeat (LTR) elements, accounting for 19.92% of the genome of *C. mollissima*, are the most abundant transposable elements (Table S4). To estimate the insertion times of the LTR elements, we identified complete LTRs using a combination of *de novo* searches and manual inspection with LTR_Finder (LTR_Finder, RRID:SCR_015247) [44]. Finally, 5,470 complete LTRs were identified. We calculated the nucleotide distance for each of the 5,470 complete LTR elements using the molecular paleontology approach described by SanMiguel et al. [45] (Fig. 3 and Table S8). The mean nucleotide distance of the LTR sequence pairs was 0.007681. When a substitution rate of 2.20 × 10^−9^ mutations per synonymous site per year was used, the insertion time distribution of the detected LTR elements indicated that the largest number of insertions occurred between 0 and 1.74 Mya [46].

### Tandemly arrayed genes

Tandemly arrayed genes (TAGs) are gene clusters created by tandem duplication, and TAGs represent a large proportion of the genes in a genome [47]. To identify TAGs, we applied OrthoMCL with the default parameters to cluster genes into putative gene families. Subsequently, 1,122 TAGs were found by an in-house script; the duplicated genes were separated by <10 spacers (Fig. S4). These gene clusters contain 4,198 tandemly duplicated genes, accounting for 11.5% of the total number of genes in *C. mollissima*, suggesting that a relatively high abundance of TAGs is a major feature of this genome. The TAGs of *C. mollissima* were compared with those of related species: *F. sylvatica* and *Q. robur* in the Fagaceae and *J. regia, M. domestica, P. persica*, and *P. trichocarpa*. The percentage of TAGs in the complete genome of *C. mollissima* was markedly higher than those of *P. trichocarpa* (4.9%) and *M. domestica* (4.2%). The TAG percentage was also high in other Fagaceae species, such as *Q. robur* (19.7%) and *F. sylvatica* (8.0%). However, this trait was not shared with *J. regia*, another species closely related to *C. mollissima*, which has only 5.6% TAGs. Furthermore, TAGs can also be highly abundant in non-Fagales species, such as *P. persica* (13.3%) (Table S9). GO enrichment analysis of genes from the TAGs was performed using OmicShare Tools [48]. The results showed that these genes are enriched in the cell binding and catalytic activity pathways in the cellular component category (Fig. S5 and Table S10).

## Conclusions

In this study, a high-quality annotated genome sequence of *C. mollissima* was obtained, similar to those of other Fagaceae species, and it was found to contain a relatively high proportion of tandemly repeated genes. The Chinese chestnut genome will serve as a reference genome and pave the way for future research involving comparative genomics, and studies of domestication, genetic improvement, and breeding for disease resistance and nut quality in chestnuts.

## Availability of supporting data and materials

Sequencing data are available via the NCBI bioproject PRJNA527178. All other supporting data and materials are available in the *GigaScience* GigaDB database [49].

## Additional files

**Table S1:** Comparison of assembly quality in 2 genomes of *C. mollissima*

**Table S2:** Statistics of clean data of *C. mollissima* for Illumina and PacBio sequencing

**Table S3:** Comparison of genome size and heterozygosity in 3 species of *C. mollissima, Q. robur*, and *F. sylvatica*

**Table S4:** Statistics of repeat elements for *C. mollissima* assembly using both RepeatModeler and RepeatMasker software

**Table S5:** Core gene estimation for *C. mollissima* assembly using BUSCO

**Table S6:** The alignment between the assembled genome and the physical map of *C. mollissima*

**Table S7:** Unique gene families of *C. mollissima* in 4 species

**Table S8:** Complete LTR elements in *C. mollissima*

**Table S9:** Numbers and proportions of TAGs in *C. mollissima* and other species

**Table S10:** Tandemly arrayed genes (TAGs) in *C. mollissima*

**Figure S1:** *k*-mer distribution of *C. mollissima*

**Figure S2:** GO term analysis for genes in *C. mollissima*

**Figure S3:** Analysis of the expanded and contracted gene families in *C. mollissima*

**Figure S4:** Tandemly arrayed gene (TAG) numbers in 1 cluster in *C. mollissima*

**Figure S5:** GO enrichment of genes from the TAGs in *C. mollissima*

giz112_GIGA-D-18-00448_Original_Submission

giz112_GIGA-D-18-00448_Revision_1

giz112_GIGA-D-18-00448_Revision_2

giz112_GIGA-D-18-00448_Revision_3

giz112_Response_to_Reviewer_Comments_Original_Submission

giz112_Response_to_Reviewer_Comments_Revision_1

giz112_Response_to_Reviewer_Comments_Revision_2

giz112_Reviewer_1_Report_Original_Submission Jean-Marc Aury -- 12/14/2018 Reviewed

giz112_Reviewer_1_Report_Revision_1 Jean-Marc Aury -- 4/18/2019 Reviewed

giz112_Reviewer_2_Report_Original_Submission Marco Thines -- 1/14/2019 Reviewed

giz112_Reviewer_2_Report_Revision_1 Marco Thines -- 4/25/2019 Reviewed

giz112_Reviewer_2_Report_Revision_2 Marco Thines -- 7/22/2019 Reviewed

giz112_Supplemental_Figures_and_Tables

## Abbreviations

BAC: bacterial artificial chromosome; BLAST: Basic Local Alignment Search Tool; bp: base pairs; BUSCO: Benchmarking Universal Single-Copy Orthologs; BWA: Burrows-Wheeler Aligner; Gb: gigabase pairs; GO: Gene Ontology; kb: kilobase pairs; KEGG: Kyoto Encyclopedia of Genes and Genomes; KOG: euKaryotic Orthologous Groups; LTR: long terminal repeat; Mb: megabase pairs; Mya: million years ago; NCBI: National Center for Biotechnology Information; PacBio: Pacific Biosciences; PE: paired-end; QTL: quantitative trait locus; RAxML: Randomized Axelerated Maximum Likelihood; TAG: tandemly arrayed genes.

## Competing interests

The authors declare that they have no competing interests.

## Authors’ contributions

Y.X. and L.Q. designed the project; Y.L., X.N., and G.W. collected samples and extracted the DNA samples; Y.X., Q.Z., H.L., Z.Z., and Y.S. worked on sequencing and data analyzing; Y.X. and Y.S. wrote the manuscript; H.H., K.F., and T.B. revised the manuscript; Q.C. and L.Q. read and approved the final version of the manuscript.
