# Supplementary material for: Hybrid de novo genome assembly of Chinese chestnut (Castanea mollissima)
Source: Gigascience. 2019 Sep 12;8(9):giz112. doi: 10.1093/gigascience/giz112 (PMC6741814; doi:10.1093/gigascience/giz112)
Supplement: giz112_GIGA-D-18-00448_Original_Submission [file giz112_giga-d-18-00448_original_submission.pdf]

GigaScience

De novo genome assembly of Chinese chestnut (Castanea mollissima)

--Manuscript Draft--

Manuscript Number:	GIGA-D-18-00448
Full Title:	De novo genome assembly of Chinese chestnut (Castanea mollissima)
Article Type:	Data Note
Funding Information:	National Key Research & Development Program of China (YS2018YFD100028) — Dr LING QIN
	National Natural Science Foundation of China (31672135) — Dr Qingqin Cao
	Project of Construction of Innovative Teams and Teacher Career Development for Universities and Colleges under Beijing Municipality (IDHT20180509) — Dr LING QIN
	Supporting Plan for Cultivating High Level Teachers in Colleges and Universities in Beijing (CIT&TCD20180317) — Dr Qingqin Cao
	National Natural Science Foundation of China (31870671) — Dr LING QIN
Abstract:	Background: Castanea mollissima is widely distributed in China for nut production. This plant also plays an important ecological role in afforestation and ecosystem services. To facilitate and expand the utilization of C. mollissima for breeding and genetic improvement, we report a whole genome sequence of C. mollissima. Findings: We produced a high-quality assembly of the C. mollissima genome using PacBio single-molecule sequencing. The final draft genome is approximately 785.53 Mb long, with a scaffold N50 size of 944 kb, and we further annotated 36,479 protein-coding genes in the genome. Phylogenetic analysis showed that C. mollissima diverged from Quercus robur, a member of the Fagaceae family, approximately 14.19 million years ago. Conclusions: The high-quality whole genome assembly of C. mollissima will be a valuable resource for further genetic improvement and breeding for disease resistance and nut quality.
Corresponding Author:	LING QIN<br><br>CHINA
Corresponding Author Secondary Information:	
Corresponding Author's Institution:	
Corresponding Author's Secondary Institution:	
First Author:	Yu Xing
First Author Secondary Information:	
Order of Authors:	Yu Xing
	Yang Liu
	Qing Zhang
	Xinghua Nie
	Yamin Sun
	Huchen Li
	Kefeng Fang

Powered by Editorial Manager® and ProduXion Manager® from Aries Systems Corporation

	Guangpeng Wang
	Hongwen Huang
	Ton Bisseling
	Qingqin Cao
	LING QIN
**Order of Authors Secondary Information:**	
**Additional Information:**	
**Question**	**Response**
Are you submitting this manuscript to a special series or article collection?	No
**Experimental design and statistics**<br><br>Full details of the experimental design and statistical methods used should be given in the Methods section, as detailed in our Minimum Standards Reporting Checklist. Information essential to interpreting the data presented should be made available in the figure legends.<br><br>Have you included all the information requested in your manuscript?	Yes
**Resources**<br><br>A description of all resources used, including antibodies, cell lines, animals and software tools, with enough information to allow them to be uniquely identified, should be included in the Methods section. Authors are strongly encouraged to cite Research Resource Identifiers (RRIDs) for antibodies, model organisms and tools, where possible.<br><br>Have you included the information requested as detailed in our Minimum Standards Reporting Checklist?	Yes
**Availability of data and materials**<br><br>All datasets and code on which the conclusions of the paper rely must be	Yes

either included in your submission or deposited in publicly available repositories (where available and ethically appropriate), referencing such data using a unique identifier in the references and in the “Availability of Data and Materials” section of your manuscript.<br><br>Have you have met the above requirement as detailed in our Minimum Standards Reporting Checklist?	

Powered by Editorial Manager® and ProduXion Manager® from Aries Systems Corporation

manuscript Click here to download Manuscript manuscript.doc

De novo genome assembly of Chinese chestnut (*Castanea mollissima*)

Yu Xing[1,2†], Yang Liu[2†], Qing Zhang[2†], Xinghua Nie[2], Yamin Sun[3], Huchen Li[1,7], Kefeng Fang[4], Guangpeng Wang[5], Hongwen Huang[6], Ton Bisseling[1,7], Qingqin Cao[1,8*], Ling Qin[1,2*]

[1] Beijing Advanced Innovation Center for Tree Breeding by Molecular Design, Beijing University of Agriculture, Beijing, China

[2] College of Plant Science and Technology, Beijing Key Laboratory for Agricultural Application and New Technique, Beijing University of Agriculture, Beijing, China

[3] Research Center for Functional Genomics and Biochip, Tianjin, China

[4] College of Landscape Architecture, Beijing Collaborative Innovation Center for Eco-Environmental Improvement with Forestry and Fruit Trees, Beijing University of Agriculture, Beijing, China

[5] Changli Institute of Pomology, Hebei Academy of Agriculture and Forestry Sciences, Changli, China

[6] South China Botanical Garden, Chinese Academy of Sciences, Guangzhou, China

[7] Laboratory of Molecular Biology, Department of Plant Sciences, Wageningen University, Wageningen, The Netherlands

[8] College of Biological Science and Engineering, Key Laboratory of Urban Agriculture (North China) Ministry of Agriculture, Beijing University of Agriculture, Beijing, China

[†] These authors contributed equally to this work.

* To whom correspondence should be addressed.

E-mails: caoqingqin@bua.edu.cn (Q.C) and qinlingbac@126.com(L.Q)

Abstract

Background: *Castanea mollissima* is widely distributed in China for nut production. This plant also plays an important ecological role in afforestation and ecosystem services. To facilitate and expand the utilization of *C. mollissima* for breeding and genetic improvement, we report a whole genome sequence of *C. mollissima*. **Findings:** We produced a high-quality assembly of the *C. mollissima* genome using PacBio single-molecule sequencing. The final draft genome is approximately 785.53 Mb long, with a scaffold N50 size of 944 kb, and we further annotated 36,479 protein-coding genes in the genome. Phylogenetic analysis showed that *C. mollissima* diverged from *Quercus robur,* a member of the Fagaceae family, approximately 14.19 million years ago. **Conclusions:** The high-quality whole genome assembly of *C. mollissima* will be a valuable resource for further genetic improvement and breeding for disease resistance and nut quality.

Keywords: *Castanea mollissima*; genome assembly; annotation; evolution

Data Description

Background information

Castanea, a member of the Fagaceae family, naturally occurs throughout the forests of eastern North America, Europe and Asia and is an ecologically and economically important Fagaceae genus. *Castanea* contains seven species. Chinese chestnut (*C. mollissima*), Chinese seguin (*C. sequinii*), Chinese chinkapin (*C. henryi*) and Japanese chestnut (*C. crenata*) occur in East Asia and show high genetic diversity [1]. The American chestnut (*C. dentata*), chinkapin (*C. pumila*) and European chestnut (*C. sativa*) are only distributed in North America and Europe, respectively, and are the predominant tree species in the deciduous forests of eastern North America and Europe [2]. Chestnuts are ubiquitous in most forests as important forest resources, providing people with many wood products and food and playing a vital role in human famine history. Chestnuts are also keystone species due to their ecological roles in afforestation and ecosystem services [3, 4].

In the *Castanea* genus, Chinese chestnut is generally considered a geographically widespread species and is widely cultivated in 26 provinces for commercial nut production in China [5]. China is rich in diverse germplasm resources of Chinese chestnut, and the cultivation of Chinese chestnut has a long history, which spans over 6000 years, according to archaeological discoveries in the Banpo Ruins of Xi'an, China. The annual yield of Chinese chestnut is high worldwide. In 2016, the Chinese chestnut production was 1,879,031 tons, accounting for 83.08 % of the world's total chestnut production that year [6]. Due to its good nut quality, easily peeled pellicle, excellent adaptability to infertile soil, and natural resistance to diseases, Chinese chestnut has been broadly used for breeding materials, especially breeding parents resistant to chestnut blight fungal pathogen (*Cryphonectria parasitica*) in the United States. An accidental introduction of the chestnut blight fungus at the beginning of the 20th century devastatingly destroyed 4 billion American chestnuts, which were a

predominant forest tree species, by 1950 [7]. Chinese chestnut has substantial levels of resistance to chestnut blight and has been utilized as a resistance resource to restore American chestnut over half a century [8, 9, 10, 11].

Despite the considerable economic and ecological importance of Chinese chestnut, the genome information available for this species is limited, which especially hinders molecular studies of main traits in nut quality and disease resistance. Therefore, in this study, we report the high-quality whole genome sequencing of *C. mollissima*. This research allows for a better understanding of the evolution of *Castanea* and produces fundamental information to facilitate and expand comparative genomic studies, domestication, breeding and genetic improvement.

Sampling and sequencing

The samples were collected from Hubei Province, China (31°16’49.25” N, 111°08’25.40” E, 1261 meter height). Genome DNA of *C. mollissima* was extracted from fresh leaves and used for sequencing (Fig. 1). Genome DNA was sheared using a sonication device for short-insert paired-end (PE) library construction. Short-insert libraries with a size of 500 bp were constructed according to the instructions described in the Illumina library preparation kit (Illumina, CA, USA). All libraries were sequenced on an Illumina HiSeq 2500 sequencer. In total, approximately 54 Gb of raw reads were generated (Table S1). For PacBio library construction, genomic DNA of *C. mollissima* was sheared to ~10 kb, and the short fragments below 7 kb were filtered using BluePipin (Sage Science, MA, USA). Filtered DNA was then converted into the proprietary SMRTbell library using the PacBio DNA Template Preparation Kit (Pacific Biosciences, CA, USA). In total, ~80 Gb of quality-filtered data were obtained from PacBio sequencing (Table S1).

Genome size and heterozygosity estimation

The distribution of short sub-sequence (k-mer) frequency, also known as the k-mer spectrum, is widely used to estimate genome size [12,13]. A k-mer depth distribution

was obtained from the Jellyfish [14] analysis, and the peak depth was clearly observed from the distribution data. The genome size was calculated as the following formula: genome size = total_k-mer_num/k-mer_depth (total_k-mer_num is the total number of k-mers from all reads, and k-mer_depth is the peak depth). Based on this method, the size of the *C. mollissima* genome was estimated to be approximately 905 Mb, and the heterozygosity rate of *C. mollissima* was approximately 0.17 % (Fig. S1).

Genome assembly and annotation

All of the subreads from PacBio sequencing were assembled using SMARTdenovo software with default values for all parameters except for –J, which had a value of 4000 (-J 4000 filters all reads with lengths less than 4000 bp) (https://github.com/ruanjue/smartdenovo). The assembly sequence was then polished using Quiver (SMRT Analysis version 2.3.0) with default parameters. To achieve a high-accuracy genome assembly, several rounds of iterative error correction were performed using Illumina clean data. 785.53 Mb of the final assembly was obtained after correction using PacBio and Illumina paired end read sequences, which comprise 2707 contigs (N50 = 944 kb, N90 = 133 kb) (Table 1). Both RepeatModeler and RepeatMasker [15] were used for the *de novo* identification and masking of repeats. To ensure the integrity of genes in the subsequent analyses, low complexity regions or simple repeats were not masked in because some of these sequences could be within genes. Finally, 49.69 % of assembled bases were masked (Table S2). The identification of protein-coding regions and gene prediction were performed through a combination of homology-based prediction, ab initio prediction, and transcriptome-based prediction method. All gene models from the above three methods were integrated by EvidenceModeler (EVM Release version 1.1.1) into a non-redundant gene set. A total of 36,479 protein-coding gene models were constructed (Table 1). The obtained gene set was functionally analyzed using BLASTP with an E-value of $1e^{-5}$ against the NCBI-NR, SwissProt, and KOG databases. Protein domains were annotated by mapping genes to the InterPro and

Pfam databases using InterProScan [16] and HMMER [17]. The potential gene pathways were derived via gene mapping against the KEGG databases. The Gene Ontology (GO) terms were extracted from the corresponding InterProScan or Pfam results (Fig. S2).

Quality assessment

To evaluate the completeness and coverage of the assembly, we aligned Illumina DNA and RNA reads against the *C. mollissima* assembly using BWA [18] and HISAT [19], respectively. The percentages of aligned DNA and RNA reads were 95.46 % and 97.41 %, respectively. In the core gene estimation using BUSCO [20], 1,392 of the 1,440 core genes (96.70 %) were completely found in the assembled genome, and 1,412 (complete BUSCOs and fragmented BUSCOs) (98.10 %) of the 1,440 core genes were at least partial matches (Table S3). This result indicates that the assembly contains mostly genetic regions, which further confirms the high quality of the *C. mollissima* genome assembly.

Alignment with physical map

A total of 19,064 BAC double-end sequences for the physical map [9] were aligned with the sequenced genome in the present study. Of these, 17,999 of the sequences were mapped onto our genome, accounting for 94.41 % of all BAC double-end sequences. The reason that 1,065 (5.59 %) of the sequences did not map to the genome is most likely due to individual differences. Then, the physical map with the above described 17,999 BAC double-end sequences was aligned with our assembled genome. The results showed that 1184 out of 1300 contigs from the physical map could be compared to our genome (Table S4).

Gene family expansion and contraction

To understand the gene family relationships of *C. mollissima* with other plants, we performed a systematic comparison of genes among different species. The full protein-coding genes of eight genomes, namely, *Oryza sativa* [21], *Malus domestica* [22], *Populus trichocarpa* [23], *Prunus persica* [24], *Castanea mollissima*, *Quercus robur* [25], *Juglans regia* [26] and *Vitis vinifera* [27], were used for the comparison. Gene loss and gain are among the primary reasons for functional changes. To gain greater insights into the evolutionary dynamics of the genes, we determined the expansion and contraction of the gene ortholog clusters of these eight species by CAFE software [28]. In the Chinese chestnut genome, a total of 16,163 gene families were identified, while 22,756 families of homologous genes were detected across the eight species. Of all the gene families (16,163), 268 significantly expanded and 60 contracted gene families ($P < 0.05$) in *C. mollissima* were examined (Fig. S3). In addition, specific and common gene families were detected between Chinese chestnut and oak. A total of 956 and 471 gene families were found to be specific to Chinese chestnut and oak, respectively (Table S5 and Table S6), and 871 common gene families were detected in these two species (Table S7). The Venn diagram in Fig. 2a shows that 9,050 gene families were shared by the four species, i.e., Chinese chestnut, apple (*M. domestica*), walnut (*J. regia*) and oak (*Q. robur*).

Phylogenetic analysis

To examine the evolutionary relationships of Chinese chestnut with other plants, we applied RAxML [29], a maximum likelihood method for genome-wide phylogenetic analysis, to 637 single-copy genes from the eight plant genomes (Fig. 2b). The results support the point of view that Chinese chestnut and oak are sister groups. The phylogenetic tree indicates that the Fagales and Rosales orders have close genetic relationships, with a divergence time of 91.09 million years ago (Mya). In the Fagales

clade, the estimated divergence time of *C. mollissima* and *Q. robur* is approximately 14.19 Mya, while that of Chinese chestnut and *J. regia* is 62.1 Mya.

Long terminal repeat (LTR) insertion

In the final assembly, approximately 390 Mb of repetitive sequences were found, accounting for 49.69 % of the genome. LTR elements accounting for 19.92 % of the genome of *C. mollissima* are the most abundant transposable elements (Table S2). To estimate the insertion times of LTR elements, we identified complete LTRs using a combination of *de novo* searches and manual inspection by LTR_Finder [30]. Finally, 5,470 complete LTRs were identified. We calculated the nucleotide distance for each of the 5,470 complete LTR elements using the molecular paleontology approach described by SanMiguel et al. [31] (Fig. 3 and Table S8). The average nucleotide distance of LTR sequence pairs is 0.007681. The insertion time distribution of the detected LTR elements showed that the largest number of insertions is between 0 and 1.74 Mya when a substitution rate of 2.20×10^{-9} mutations per synonymous site per year was used [32].

Tandemly arrayed genes

Tandemly arrayed genes (TAGs) are gene clusters created by tandem duplication, and TAGs represent a large proportion of the genes in a genome. To identify TAGs, we applied OrthoMCL with default parameters to cluster genes in putative gene families. Finally, 1,122 TAGs were found by an in-house script that duplicated genes were separated by less than 10 spacers (Fig. S4). These gene clusters contain 4,198 tandemly duplicated genes, accounting for 11.5 % of the total genes in *C. mollissima*, suggesting that an abundance of TAGs is a major feature of the genome. GO enrichment analysis of genes from the TAGs was performed using OmicShare Tools (https://omictools.com/). The results show that the genes are enriched in cell binding and catalytic activity pathways in the cellular component category (Fig. S5 and Table S9).

Conclusions

In this study, a high quality whole genome assembly of *C. mollissima* was obtained with genome sequencing, assembly, and annotation, and tandem genes were reported. The Chinese chestnut genome will serve as a reference genome and pave the way for future research on comparative genomic investigations, domestication, genetic improvement and breeding for disease resistance and nut quality of chestnut.

Availability of supporting data

Additional files

Table S1: Statistics of clean data for *C.mollissima* for Illumina and PacBio sequencing

Table S2: Statistics of repeat elements for *C.mollissima* assembly using both RepeatModeler and RepeatMasker software

Table S3: Core gene estimation for *C.mollissima* assembly using CEGMA

Table S4: The alignment between the assembled genome and the physical map of *C.mollissima*

Table S5: Unique gene families of *C.mollissima* in eight species

Table S6: Unique gene families of *Q. robur* in eight species

Table S7: Unique gene families of *C.mollissima* and *Q. robur* in four species

Table S8: Complete LTR elements in *C.mollissima*

Table S9: Tandemly arrayed genes (TAGs) in *C.mollissima*

Figure S1: k-mers estimation

Figure S2: GO term analysis for genes in *C. mollissima*

Figure S3: Gene families expanded and contracted analysis in *C. mollissima*

Figure S4: Tandemly arrayed genes (TAGs) numbers in one cluster in *C. mollissima*

Figure S5: GO enrichment of genes from the TAGs in *C. mollissima*

Competing interests

The authors declare that they have no competing interests.

Authors' contributions

YX and LQ designed the project; YL, XN and GW collected samples and extracted the DNA samples; YX, QC, QZ, HL and YS worked on sequencing and data analyzing; YX and YS wrote the manuscript; HH, KF, and TB revised the manuscript; QC and LQ read and approved the final version of the manuscript.

Acknowledgements

This work was supported by grants from the National Key Research & Development Program of China (YS2018YFD100028); the National Natural Science Foundation of China (31870671; 31672135); the Project of Construction of Innovative Teams and Teacher Career Development for Universities and Colleges under Beijing Municipality (IDHT20180509); Supporting Plan for Cultivating High Level Teachers in Colleges and Universities in Beijing (CIT&TCD20180317).

References

1. Lang P, Dane F, Kubisiak TL, et al. Molecular evidence for an Asian origin and a unique westward migration of species in the genus *Castanea* via Europe to North America. Molecular Phylogenetics and Evolution 2007; **43** (1): 49-59. https://doi.org/10.1016/j.ympev.2006.07.022.
2. Staton M, Zhebentyayeva T, Olukolu B, et al. Substantial genome synteny preservation among woody angiosperm species: comparative genomics of Chinese chestnut (*Castanea mollissima*) and plant reference genomes. BMC Genomics 2015; **16** (1): 744-57. https://doi.org/10.1186/s12864-015-1942-1.

3. Martín MA, Herrera MA, and Martín LM. In situ conservation and landscape genetics in forest species. Journal of Natural Resources and Development 2012; **2** (3): 1-5. https://doi.org/10.5027/jnrd.v2i0.01.
4. Zou F, Guo SJ, Xie P, et al. Megasporogenesis and development of female gametophyte in Chinese chestnut (*Castanea mollissima*) cultivar 'yanshanzaofeng'. International Journal of Agriculture and Biology 2014; **16** (5): 1001-1005. https://search.proquest.com/docview/1606411431?accountid=43808.
5. Lang P, Dane F, Kubisiak TL. Phylogeny of *Castanea* (Fagaceae) based on chloroplast *trn*T-L-F sequence data. Tree Genetics & Genomes 2006; **2** (3): 132-139. https://doi.org/10.1007/s11295-006-0036-2.
6. FAO. Food and Agriculture Organization of the United Nations. FAOSTAT Statistics Database 2016. Available from: http://www.fao.org/faostat/en/#home. Accessed 08 Feb 2017.
7. Kremer A, Abbott AG, Carlson JE, et al. Genomics of Fagaceae. Tree Genetics & Genomes 2012; 8 (3): 583-610. https://doi.org/10.1007/s11295-012-0498-3.
8. Kubisiak TL, Nelson CD, Staton ME, et al. A transcriptome-based genetic map of Chinese chestnut (*Castanea mollissima*) and identification of regions of segmental homology with peach (*Prunus persica*). Tree Genetics & Genomes 2013; **9** (2): 557-571. https://doi.org/10.1007/s11295-012-0579-3.
9. Fang GC, Blackmon BP, Staton ME, et al. A physical map of the Chinese chestnut (*Castanea mollissima*) genome and its integration with the genetic map. Tree Genetics & Genomes 2013; **9** (2): 525-537. https://doi.org/10.1007/s11295-012-0576-6.
10. Barakat A, DiLoreto DS, Zhang Y, et al. Comparasion of the transcriptomes of American chestnut (*Castanea dentata*) and Chinese chestnut (*Castanea mollissima*) in response to the chestnut blight infection. BMC Plant Biology 2009; **9** (1): 51-62. https://doi.org/10.1186/1471-2229-9-51.
11. Santos C, Nelson CD, Zhebentyayeva T, et al. First interspecific genetic linkage map for *Castanea sativa* × *Castanea crenata* revealed QTLs for resistance to

Phytophthora cinnamomi. Plos One 2017; **12** (9): e0184381. https://doi.org/10.1371/journal.pone.0184381.

12. Li M, Tian S, Jin L,et al. Genomic analyses identify distinct patterns of selection in domesticated pigs and Tibetan wild boars. Nature genetics 2013; 45 (12): 1431-1438. https://doi.org/10.1038/ng.2811.
13. Zhang T, Hu Y, Jiang W, et al. Sequencing of allotetraploid cotton (*Gossypium hirsutum* L. acc. TM-1) provides a resource for fiber improvement. Nature Biotechnology 2015; 33(5): 531-537. https://doi.org/10.1038/nbt.3207.
14. Marçais G, Kingsford C. A fast, lock-free approach for efficient parallel counting of occurrences of *k*-mers. Bioinformatics 2011; **27** (6): 764-770. https://doi.org/10.1093/bioinformatics/btr011.
15. Tarailo-Graovac M, Chen NS. Using RepeatMasker to identify repetitive elements in genomic sequences. Current Protocols in Bioinformatics 2009; **25**: 4.10.1-4.10.14. https://doi.org/10.1002/0471250953.bi0410s25.
16. Jones P, Binns D, Chang HY, et al. InterProScan 5: genome-scale protein function classification. Bioinformatics 2014; **30** (9): 1236-1240. https://doi.org/10.1093/bioinformatics/btu031.
17. Wheeler TJ, Eddy SR. nhmmer: DNA homology search with profile HMMs. Bioinformatics 2013; **29** (19): 2487-2489. https://doi.org/10.1093/bioinformatics/btt403.
18. Li H, Durbin R. Fast and accurate long-read alignment with Burrows-Wheeler transform. Bioinformatics 2010; **26** (5): 589-595. https://doi.org/10.1093/bioinformatics/btp698.
19. Kim D, Langmead B, Salzberg SL. HISAT: a fast spliced aligner with low memory requirements. Nature Methods 2015; **12** (4): 357-360. https://doi.org/10.1038/nmeth.3317.
20. Waterhouse RM, Seppey M, Simão FA, et al. BUSCO applications from quality assessments to gene prediction and phylogenomics. Molecular Biology and Evolution 2018; **35** (3): 543-548. https://doi.org/10.1093/molbev/msx319.

21. International Rice Genome Sequencing Project. The map-based sequence of the rice genome. Nature 2005; **436** (7052): 793-800. https://doi.org/10.1038/nature03895.
22. Velasco R, Zharkikh A, Affourtit J, et al. The genome of the domesticated apple (*Malus* × *domestica* Borkh.). Nature Genetics 2010; **42** (10): 833-839. https://doi.org/10.1038/ng.654.
23. Tuskan GA, Difazio S, Jansson S, et al. The genome of black cottonwood, *Populus trichocarpa* (Torr. & Gray). Science 2006; **313** (5793): 1596-1604. https://doi.org/10.1126/science.1128691.
24. Verde I, Abbott AG, Scalabrin S, et al. The high-quality draft genome of peach (*Prunus persica*) identifies unique patterns of genetic diversity, domestication and genome evolution. Nature Genetics 2013; **45** (5): 487-494. https://doi.org/10.1038/ng.2586.
25. Plomion C, Aury JM, Amselem J, et al. Oak genome reveals facets of long lifespan. Nature Plants 2018; **4** (7): 440-452. https://doi.org/10.1038/s41477-018-0172-3.
26. Martínez-García PJ, Crepeau MW, Puiu D, et al. The walnut (*Juglans regia*) genome sequence reveals diversity in genes coding for the biosynthesis of non-structural polyphenols. The Plant Journal 2016; **87** (5): 507-532. https://doi.org/10.1111/tpj.13207.
27. The French-Italian Public Consortium for Grapevine Genome Characterization. The grapevine genome sequence suggests ancestral hexaploidization in major angiosperm phyla. Nature 2007; **449** (7161): 463-467. https://doi.org/10.1038/nature06148.
28. De Bie T, Cristianini N, Demuth J, et al. CAFE: a computational tool for the study of gene family evolution. Bioinformatics 2006; **22**(10): 1269-1271. https://doi.org/10.1093/bioinformatics/btl097.
29. Stamatakis A. RAxML version 8: a tool for phylogenetic analysis and post-analysis of large phylogenies. Bioinformatics 2014; **30** (9): 1312-1313. https://doi.org/10.1093/bioinformatics/btu033.

30. Xu Z, Wang H. LTR_FINDER: an efficient tool for the prediction of full-length LTR retrotransposons. Nucleic Acids Research 2007; **35**(Web Server issue): W265-W268. https://doi.org/10.1093/nar/gkm286.

31. SanMiguel P, Gaut BS, Tikhonov A, et al. The paleontology of intergene retrotransposons of maize. Nature Genetics 1998; **20** (1): 43-45. https://doi.org/10.1038/1695.

32. Björn N, Nathaniel RS, Anna W, et al. The Norway spruce genome sequence and conifer genome evolution. Nature 2013, 497 (7451): 579-584. https://doi.org/10.1038/nature12211.

 Click here to download Table Tables.doc

Table：

Table 1 Summary of *C.mollissima* genome assembly and gene model

**Genome assembly statistics**	
Total length	785,529,252 bp
Number of Contigs	2,707
Largest Contig Length	6,584,328 bp
N50 length (Contigs)	944,461 bp
N90 length (Contigs)	133,678 bp
Counts of N50 (Contigs)	235
Counts of N90 (Contigs)	1,024
**Gene model statistics**	
Gene number	36,479
Gene density (per 100kb)	4.64
Gene average length	1,139.63 bp
Exon number per Gene	4.41
Exon average length	258.15 bp
Intron average length	1,156.91 bp
Genome GC percent	36.07 %
Exon GC percent	43.36 %

 Click here to download Figure Figures.doc

Figure:

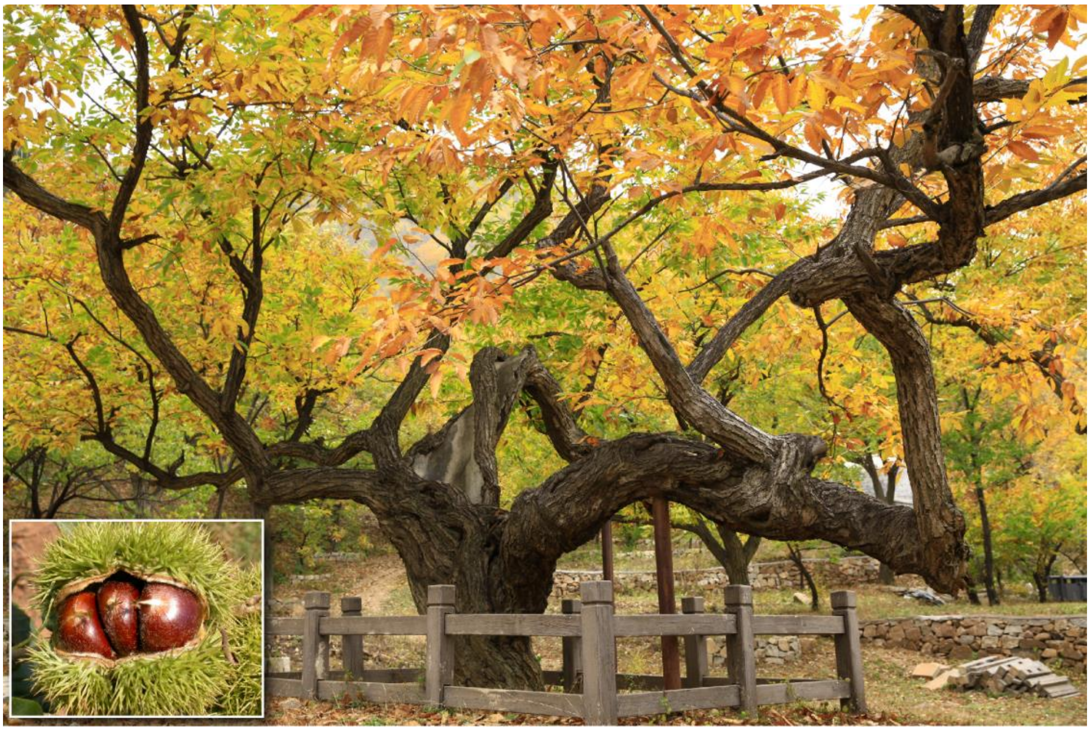

Figure 1 Example of Chinese chestnut tree (*C. mollissima*). Natural habitat of *C. mollissima* (image from the Water Great Wall, Beijing, China) and the nut of *C. mollissima* (image from Ling Qin) are showed.

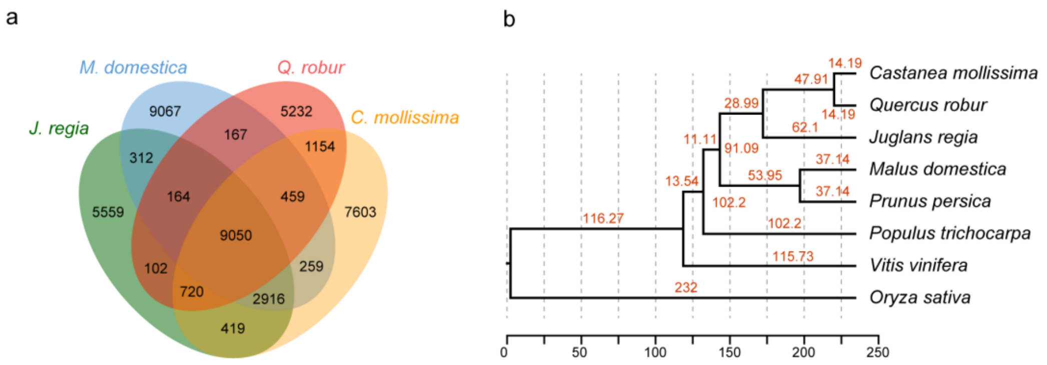

Figure 2 Phylogenetic relationships between Chinese chestnut and other plants. Maximum-likelihood tree was obtained with 637 single copy orthologous genes. a) The shared and unique gene families in four closed species are shown in the Venn diagram. Each number represents a gene family number. b) The divergence times were estimated and are displayed on the phylogenetic tree.

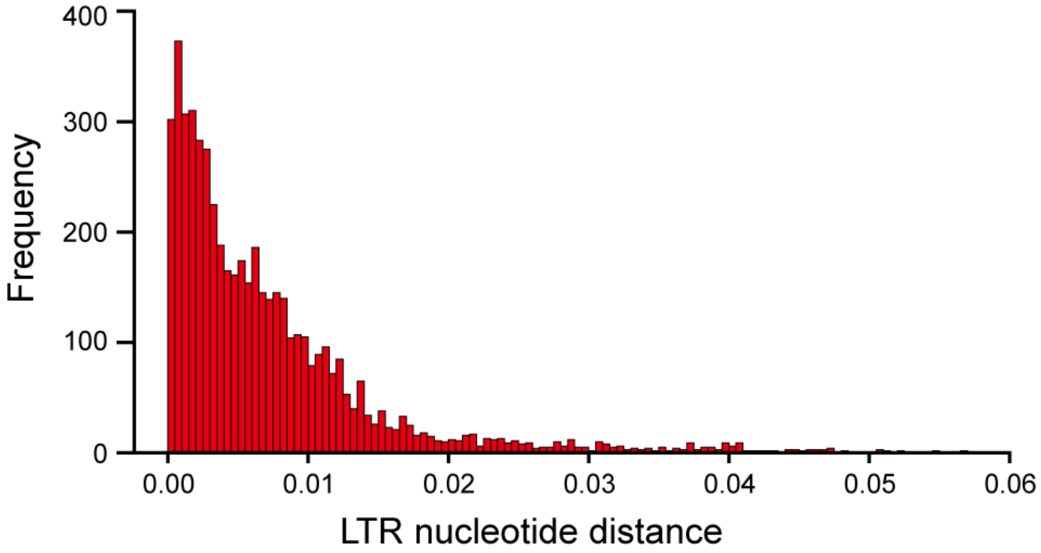

Figure 3 Nucleotide distance distribution of annotated LTR elements in *C.mollissima*.

Figure S1-S5

Click here to access/download
Supplementary Material
Figure (S1-S5).doc

Table S1-S3

Click here to access/download
Supplementary Material
Tables (S1-S3).doc

Table S4

Click here to access/download
Supplementary Material
Table S4.xls

Table S5

Click here to access/download
Supplementary Material
Table S5.xls

Table S6

Click here to access/download
Supplementary Material
Table S6.xls

Table S7

Click here to access/download
Supplementary Material
Table S7.xls

Table S8

Click here to access/download
Supplementary Material
Table S8.xls

Table S9

Click here to access/download
Supplementary Material
Table S9.xls

Click here to access/download
Supplementary Material
protocols.doc
