# Supplementary material for: Hybrid de novo genome assembly of Chinese chestnut (Castanea mollissima)
Source: Gigascience. 2019 Sep 12;8(9):giz112. doi: 10.1093/gigascience/giz112 (PMC6741814; doi:10.1093/gigascience/giz112)
Supplement: giz112_GIGA-D-18-00448_Revision_1 [file giz112_giga-d-18-00448_revision_1.pdf]

GigaScience

Hybrid de novo genome assembly of Chinese chestnut (Castanea mollissima)

--Manuscript Draft--

**Manuscript Number:**	GIGA-D-18-00448R1
**Full Title:**	Hybrid de novo genome assembly of Chinese chestnut (Castanea mollissima)
**Article Type:**	Data Note
**Funding Information:**	National Key Research & Development Program of China (2018YFD1000605) — Dr LING QIN<br>National Natural Science Foundation of China (31870671) — Dr LING QIN<br>National Natural Science Foundation of China (31672135) — Dr Qingqin Cao<br>Project of Construction of Innovative Teams and Teacher Career Development for Universities and Colleges under Beijing Municipality (IDHT20180509) — Dr LING QIN<br>Supporting Plan for Cultivating High Level Teachers in Colleges and Universities in Beijing (CIT&TCD20180317) — Dr Qingqin Cao
**Abstract:**	Background: Castanea mollissima is widely cultivated in China for nut production. This plant also plays an important ecological role in afforestation and ecosystem services. To facilitate and expand the utilization of C. mollissima for breeding and its genetic improvement, we report here the whole genome sequence of C. mollissima. Findings: We produced a high-quality assembly of the C. mollissima genome using PacBio single-molecule sequencing. The final draft genome is approximately 785.53 Mb long, with a contig N50 size of 944 kb, and we further annotated 36,479 protein-coding genes in the genome. Phylogenetic analysis showed that C. mollissima diverged from Quercus robur, a member of the Fagaceae family, approximately 13.62 million years ago. Conclusions: The high-quality whole genome assembly of C. mollissima will be a valuable resource for further genetic improvement and breeding for disease resistance and nut quality.<br>Keywords: Castanea mollissima; genome assembly; annotation; evolution
**Corresponding Author:**	LING QIN<br><br>CHINA
**Corresponding Author Secondary Information:**	
**Corresponding Author's Institution:**	
**Corresponding Author's Secondary Institution:**	
**First Author:**	Yu Xing
**First Author Secondary Information:**	
**Order of Authors:**	Yu Xing<br>Yang Liu<br>Qing Zhang<br>Xinghua Nie<br>Yamin Sun<br>Zhiyong Zhang

Powered by Editorial Manager® and ProduXion Manager® from Aries Systems Corporation

	Huchen Li
	Kefeng Fang
	Guangpeng Wang
	Hongwen Huang
	Ton Bisseling
	Qingqin Cao
	LING QIN
**Order of Authors Secondary Information:**	
**Response to Reviewers:**	We have also uploaded our response to reviewers' comments in the attach files named 'Reviewers_comments' because there are some figures and tables in the file of response to reviewers' comments. Please see the detailed response in the attached files. We also listed the response to the two reviewers' comments without figures and tables here.<br><br>Reviewer 1:<br><br>The article describes the sequencing, assembly and annotation of the Chinese chestnut tree (Castanea mollisima) as well as some analysis based on gene families. My main concern about the manuscript is related to the availability of the sequencing data. Indeed the genomic and transcriptomic data are not available through SRA portal. On the other hand, the comparative analyses are not of equal quality. For example, the functional enrichment of specific gene families has not been inspected when compared to the TAGs. In addition, a few points need to be addressed.<br><br>A: For the reviewers’ convenience, we have already uploaded our raw data to the NCBI SRA database; the BioProject # is PRJNA527178 (SRA # of transcriptome: SRR8731962, SRA # of PacBio reads: SRR8755082-SRR8755097, and SRA # of Illumina reads: SRR8731963), and we have also confirmed that all the data are available now.<br><br>Sampling and Sequencing:<br>- Line 31: Genome DNA instead of Genomic DNA.<br><br>A: Yes. We have revised it in the line 82 on page 4 of the revised manuscript.<br><br>- Line 39: approximately 54 Gb, where Table S1 reports 39 Gb.<br><br>A: The data in Table S1 are correct, and we have double-checked and verified the data in the revised manuscript and tables and corrected the typos in the revised manuscript.<br><br>- Line 49: In total 80 Gb, where Table S1 reports 69 Gb.<br><br>A: The data in Table S1 are correct, and we have double-checked and verified the data in the revised manuscript and tables and corrected the typos in the revised manuscript.<br><br>Genome size and heterozygosity estimation:<br>- The authors report a heterozygosity rate of approximately 0.17%, I think it is of interest to compare this rate with other trees, like beech or oak.<br><br>A: The genome size and heterozygosity rate were evaluated before we started the genome sequencing project, and the heterozygosity rate that we obtained was 0.17%. Based on our common sense and a comparison with data from other trees, we thought the results were unreasonable. Therefore, we double-checked the raw data and found some bacterial contamination in the Illumina raw data, which caused low coverage depth and resulted in inaccurate genome size and heterozygosity rate estimations. Therefore, we resequenced the samples without bacterial contamination using the Illumina HiSeq 2500 sequencer and then used the new data to perform the same analyses (genome size and heterozygosity estimation, genome assembly and error correction). However, the initial genome size and heterozygosity estimates (0.17%)

Powered by Editorial Manager® and ProduXion Manager® from Aries Systems Corporation

from the previous report using the contaminated data were mistakenly used in the manuscript submitted for review. We have corrected these estimates in the revised manuscript, and the correct heterozygosity rate should be 0.87%. Comparing this estimate with those for beech (heterozygosity rate 0.19%) and oak (heterozygosity rate ~1%), we found that our result was more similar to oak (Figure S1).

Genome assembly and annotation:
- The authors describe the polishing step made using Quiver, but it is unclear how the several (how many?) rounds of iterative error correction, with short reads, were performed.

A：We performed six rounds of interactive error correction with the Illumina data and have added this information to line 111-113 on page 5 of the revised manuscript.

- The annotation process is not sufficiently described. The authors should describe which databases and which tools were used, and they should describe the transcriptomic dataset.

A: In the revised manuscript, we describe the annotation process in detail. In addition, we describe a transcriptomic dataset, which has been uploaded to the SRA database. We validated the exon/intron boundaries using transcripts and the GU-AG rule. We identified 36,479 protein-coding genes in the C. mollissima genome. In detail, 47,666 genes were predicted through the de novo method, 26,647 genes were annotated based on RNA transcripts or raw RNA reads, and 14,695 genes were supported by homology. We also listed the gene numbers that could be annotated in the different databases. For your convenience, we listed the gene numbers that we used and integrated to predict our protein-coding genes in the following table.

Quality assessment:
- line 28: genetic regions instead of genic regions.

A: Yes. We have revised it in the line 152 on page 7 of the revised manuscript.

Gene family expansion and contraction
- A total of 956 and 471 gene families were found to be specific to Chinese chestnut and oak. However, on Figure 2a that refers to gene families, the numbers are different. The authors should clarify the text and/or the legend of Figure 2.

A: We have corrected these numbers in the revised manuscript. In addition, in accordance with the reviewers' suggestions, we adjusted the text and the legend of Figure 2a to increase clarity, and we recreated the Venn diagram using C. mollissima, Q. robur, J. regia and F. sylvatica. Please see the "Gene family expansion and contraction" section (lines 163-178, pages 7-8) of the revised manuscript (Figure 2).

Tandemly arrayed genes
- The authors claim that "TAGs are gene clusters created by tandem duplication, and TAGs represent a large proportion of the genes in a genome". Authors should add citations to support this sentence.

A: We have cited a reference to support this sentence.
Pan D, Zhang LQ. Tandemly arrayed genes in vertebrate genomes. Comparative and functional genomics 2008; 2008: 1-11. https://doi.org/10.1155/2008/545269.

- The authors suggest that an abundance of TAGs is a major feature if the genome, but they should compare to other species, such as the oak genome which contains a high proportion of TAGs (as shown in Plomion et al, 2018).

A: According to this suggestion, we compared the number and proportion of TAGs in different species and added them to the revised manuscript as supplementary Table S7. Please see the "Tandemly arrayed genes" section (lines 204-222, page 9) of the revised manuscript (Table S7).

Reviewer2: First of all, I congratulate the authors on there effort and the valuable results obtained. The genome seems mostly well assembled, but there are some

Powered by Editorial Manager® and ProduXion Manager® from Aries Systems Corporation

issues as outlined below that should be addressed.

The English is of some concern. There are only few grammar and spelling mistakes, but there are many sentences that are phrased in an odd manner, sometimes there is also highly unusual word usage. The authors are thus strongly encouraged to have their manuscript proofread by a native English speaker.

A: The revised manuscript has been polished by a senior editor at American Journal Experts (https://secure.aje.com, contract #6G58F9VV) and proofread by Professor Rene Geurts, from Department of Molecular Biology, Wageningen University and Professor Jocelyn Rose, from School of Integrative Plant Science, Cornell University.

Title. The title should mention that a hybrid approach was used.

A: Yes. We have revised the title as follows: "Hybrid de novo genome assembly of Chinese chestnut (Castanea mollissima)".

Page 5
Line 24. Castanea sativa has dominant role as forest tree only in few areas of Europe (e.g. some parts of northern Italy and southern France).
Lines 42-44. A citation should be given for this claim.
Lines 51-57. A citation should be given for this claim.

A: Yes. We have revised these passages and cited the references in the manuscript in lines 50-51, 55-58, and 60-64 on page 3 of the revised manuscript.

Page 6
Line 9. The available resources should be cited here, e.g. the genome published previously.

A: Yes. We have cited the references in the manuscript in lines 69-72 on page 4 of the revised manuscript.

Line 28. The sequenced indivual(s) should be described in more detail and the sampling date should be given.

A: Yes. We have described the sampling information in more detail in lines 77-83 on page 4 of the revised manuscript.

Genome assembly. As SMARTdenovo does not have a correction step, all data should have been corrected before assembly. At 100x, it might have been useful doing self-correction rather than Illumina-based filtering (first).

A: We assembled the genome using three software packages. Interestingly, the results indicated that the genome assembly performed with SMARTdenovo displayed higher continuity than those from the other two software packages. To achieve a high-accuracy genome assembly, the assembled sequence was then polished using Quiver (SMRT Analysis v2.3.0) with the default parameters, and six rounds of iterative error correction were performed using the Illumina clean reads.

Page 7
Lines 46-49. More detail needs to be provided here. From the information given, it is unclear how gene predictions were actually done.
A: Yes. We have added a detailed description to the "Genome assembly and annotation" section (lines 107-143, pages 5-6) of the revised manuscript.
Line 51. The output from the evidence modeler could be checked against transcripts to vailidate exon/intron boundaries.

A: We validated the exon/intron boundaries using transcripts and the GU-AG rule. We identified 36,479 protein-coding genes in the C. mollissima genome. In detail, 47,666 genes were predicted through the de novo method, 26,647 genes were annotated by RNA transcripts or raw RNA reads, and 14,695 genes were supported by homology. We also listed the numbers of genes that could be annotated with the different databases. For your convenience, we listed the gene numbers that we used and

	integrated to predict the protein-coding genes in the following table.<br><br>Page 8<br>Line 28. Most?<br><br>A: We revised the manuscript to improve clarity in line 152 on page 7.<br><br>Page 9<br>Gene family expansion and contraction/phylogenetic analysis. For both of these analyses, the genome of Fagus sylvatica should be included as well as an important reference point in Fagaceae. In the Venn diagram, it could replace Malus domestica, which is largely unrelated to chestnut and oak.<br><br>A: Yes. We have corrected this issue in the revised manuscript (lines 163-178, pages 7-8). We recreated the Venn diagram using C. mollissima, Q. robur, J. regia and F. sylvatica. In addition, in accordance with the reviewers' suggestions, we adjusted the text and the legend of Figure 2a to improve clarity (Figure 2).<br><br>Line 48. RAxML is a program not a method. It should be mentioned, which version and which substitution model were used, as well as the amount of bootstrap replicated done.<br><br>A: Yes. We have revised this text in lines 181-182 on page 8 of the revised manuscript.<br><br>Line 57. It should be mentioned where these divergence times are coming from. the authors could also do their own estimations using a dating software.<br><br>A: We estimated these divergence times using r8s. We selected three time scales from the time tree (http://www.timetree.org/). We also revised the manuscript to improve clarity.<br><br>Page 10<br>Tandemly arranged genes. This should be specifically compared to Quercus, Fagus and a species outside Fagaceae, such as walnut and poplar, to investigate, how special this trait is.<br><br>A: The tandemly arranged genes of C. mollissima were compared with those of closely related species in the genomes of F. sylvatica and Q. robur in the Fagaceae and the additional genomes of J. regia, M. domestica, P. persica and P. trichocarpa. The results are indicated in the "Tandemly arrayed genes" section (lines 204-222, page 9) of the manuscript and have also been added to the supplementary information as supplementary Table S7.
**Additional Information:**	
**Question**	**Response**
Are you submitting this manuscript to a special series or article collection?	No
**Experimental design and statistics**<br><br>Full details of the experimental design and statistical methods used should be given in the Methods section, as detailed in our Minimum Standards Reporting Checklist. Information essential to interpreting the data presented should be made available in the figure legends.	Yes

Have you included all the information requested in your manuscript?	
**Resources**<br><br>A description of all resources used, including antibodies, cell lines, animals and software tools, with enough information to allow them to be uniquely identified, should be included in the Methods section. Authors are strongly encouraged to cite Research Resource Identifiers (RRIDs) for antibodies, model organisms and tools, where possible.<br><br>Have you included the information requested as detailed in our Minimum Standards Reporting Checklist?	Yes
**Availability of data and materials**<br><br>All datasets and code on which the conclusions of the paper rely must be either included in your submission or deposited in publicly available repositories (where available and ethically appropriate), referencing such data using a unique identifier in the references and in the “Availability of Data and Materials” section of your manuscript.<br><br>Have you have met the above requirement as detailed in our Minimum Standards Reporting Checklist?	Yes

Revised Manuscript
Click here to view linked References
Click here to access/download;Manuscript;Revised manuscript.doc

Hybrid *de novo* genome assembly of Chinese chestnut (*Castanea mollissima*)

Yu Xing[1,2†], Yang Liu[2†], Qing Zhang[2†], Xinghua Nie[2], Yamin Sun[3], Zhiyong Zhang[1,2], Huchen Li[1,7], Kefeng Fang[4], Guangpeng Wang[5], Hongwen Huang[6], Ton Bisseling[1,7], Qingqin Cao[1,8*], Ling Qin[1,2*]

[1] Beijing Advanced Innovation Center for Tree Breeding by Molecular Design, Beijing University of Agriculture, Beijing, 102206, China

[2] College of Plant Science and Technology, Beijing Key Laboratory for Agricultural Application and New Technique, Beijing University of Agriculture, Beijing, 102206, China

[3] Research Center for Functional Genomics and Biochip, Tianjin, 300457, China

[4] College of Landscape Architecture, Beijing Collaborative Innovation Center for Eco-Environmental Improvement with Forestry and Fruit Trees, Beijing University of Agriculture, Beijing, 102206, China

[5] Changli Institute of Pomology, Hebei Academy of Agriculture and Forestry Sciences, Changli, 066600, China

[6] South China Botanical Garden, Chinese Academy of Sciences, Guangzhou, 510650, China

[7] Laboratory of Molecular Biology, Department of Plant Sciences, Wageningen University, Wageningen, 6708 PB, The Netherlands

[8] College of Biological Science and Engineering, Key Laboratory of Urban Agriculture (North China) Ministry of Agriculture, Beijing University of Agriculture, Beijing, 102206, China

[†] These authors contributed equally to this work.

* To whom correspondence should be addressed.

E-mails: caoqingqin@bua.edu.cn (Q.C) and qinlingbac@126.com(L.Q)

Abstract

Background: *Castanea mollissima* is widely cultivated in China for nut production. This plant also plays an important ecological role in afforestation and ecosystem services. To facilitate and expand the utilization of *C. mollissima* for breeding and its genetic improvement, we report here the whole genome sequence of *C. mollissima*. **Findings:** We produced a high-quality assembly of the *C. mollissima* genome using PacBio single-molecule sequencing. The final draft genome is approximately 785.53 Mb long, with a contig N50 size of 944 kb, and we further annotated 36,479 protein-coding genes in the genome. Phylogenetic analysis showed that *C. mollissima* diverged from *Quercus robur*, a member of the Fagaceae family, approximately 13.62 million years ago. **Conclusions:** The high-quality whole genome assembly of *C. mollissima* will be a valuable resource for further genetic improvement and breeding for disease resistance and nut quality.

Keywords: *Castanea mollissima*; genome assembly; annotation; evolution

Data Description

Background information

Castanea, a genus of the Fagaceae family, occurs naturally throughout the forests of eastern North America, Europe and Asia, where it is ecologically and economically important. *Castanea* contains seven species. Chinese chestnut (*C. mollissima*), Chinese seguin (*C. seguinii*), Chinese chinkapin (*C. henryi*) and Japanese chestnut (*C. crenata*) occur in East Asia and show high genetic diversity [1]. The American chestnut (*C. dentata*) and chinkapin (*C. pumila*) occur only in North America, while the European chestnut (*C. sativa*) distributes in Europe, and they are the predominant tree species in the deciduous forests of eastern North America and some parts of northern Italy and southern France [2]. Chestnuts are important forest resources that provide wood products and food, and they are also keystone species due to their ecological roles in afforestation and ecosystem services [3].

Chinese chestnut is geographically widespread and is cultivated in 26 Chinese provinces for commercial nut production [4]. China is rich in diverse germplasm resources of Chinese chestnut, and the cultivation of Chinese chestnut has a long history, which spans over 6000 years, according to archeological discoveries in the Banpo Ruins of Xi'an, China [5]. The annual nut yield of Chinese chestnut is high. In 2017, Chinese chestnut production was 1,939,719 tonnes, accounting for 83.34 % of the world's total chestnut production that year [6]. Due to its high nut quality, easily peeled pellicle, excellent adaptability to infertile soil, and natural resistance to diseases, Chinese chestnut has been broadly used in breeding programs, especially to introduce resistance to the chestnut blight fungal pathogen (*Cryphonectria parasitica*) in the United States [7, 8]. An accidental introduction of the chestnut blight fungus at the beginning of the 20th century destroyed 4 billion American chestnuts, which were a predominant forest tree species, by 1950 [9, 10, 11]. Chinese chestnut has substantial levels of resistance to chestnut blight and has been utilized as a resistance resource to restore American chestnut [7].

Despite the considerable economic and ecological importance of Chinese chestnut, the genome information available for this species is limited, which has hindered molecular studies of the major traits involved in nut quality and disease resistance [12, 13, 14]. In this study, we report a high-quality whole genome sequence of *C. mollissima*. This research allows for a better understanding of the evolution of *Castanea* and produces fundamental information to facilitate and expand comparative genomic studies, domestication, breeding and genetic improvement.

Sampling and sequencing

A mature, healthy tree of wild *C. mollissima* was chosen from the Zhangcunping national forest reserve (31°16’49.25” N, 111°08’25.40” E, 1261 meter altitude) of the city of Yichang in Hubei Province, China. The individual measured ~ 12 m in height, and its trunk was ~10 cm in diameter (at breast height). Fresh leaves were collected on 18 June 2017. The samples were immediately frozen in liquid nitrogen and then stored at -80℃. The genomic DNA of *C. mollissima* was extracted using the DNeasy Plant Mini Kit (Qiagen, Hilden, Germany) and used for sequencing (Fig. 1). Then, the DNA was sheared using a sonication device for short-insert paired-end (PE) library construction. Short-insert libraries with a size of 500 bp were constructed according to the instructions described in the Illumina library preparation kit (Illumina, CA, USA). All libraries were sequenced on an Illumina HiSeq 2500 sequencer with the PE 2×150 bp protocol. In total, approximately 34 Gb of clean data were generated, yielding a sequencing depth of ~42.7 X (Table S1). For PacBio library construction, the genomic DNA of *C. mollissima* was sheared to 20 kb, and fragments shorter than 7 kb were filtered using BluePippin (Sage Science, MA, USA). The filtered DNA was then used to prepare a proprietary SMRTbell library using the PacBio DNA Template Preparation Kit (Pacific Biosciences, CA, USA). In total, ~69 Gb of quality-filtered data were obtained from PacBio sequencing with an average read length of 7,170 bp and a sequencing depth of ~87 X (Table S1).

Genome size and heterozygosity estimation

The distribution of short subsequence (k-mer) frequency, also known as the k-mer spectrum, is widely used to estimate genome size [15, 16]. A k-mer depth distribution was obtained from a Jellyfish [17] analysis, and the peak depth was clearly observed from the distribution data. The genome size was calculated with the following formula: genome size = total_k-mer_num/k-mer_depth (total_k-mer_num is the total number of k-mers from all reads, and k-mer_depth is the peak depth). Based on this method, the size of the *C. mollissima* genome was estimated to be approximately 772 Mb, and the heterozygosity level of *C. mollissima* was approximately 0.87 % (Fig. S1).

Genome assembly and annotation

All of the subreads from PacBio sequencing were assembled using SMARTdenovo software with default values for all parameters except for -J, which was set to a value of 4000 (-J 4000 filters all reads with lengths less than 4,000 bp) (https://github.com/ruanjue/smartdenovo). The assembled sequence was then polished using Quiver (SMRT Analysis version 2.3.0) with the default parameters. To achieve a high-accuracy genome assembly, six rounds of iterative error correction were performed using the clean Illumina data. In total, 785.53 Mb of final assembly was obtained after correction using PacBio and Illumina PE read sequences, and the assembly comprised 2,707 contigs (N50 = 944 kb, N90 = 133 kb) (Table 1). Both RepeatModeler and RepeatMasker [18] were used for the *de novo* identification and masking of repeats. To ensure the integrity of genes in the subsequent analyses, low-complexity regions or simple repeats were not masked because some of these sequences could be within genes. Finally, 49.69 % of the assembled bases were masked (Table S2). Protein-coding region identification and gene prediction were performed through a combination of ab initio prediction, homology-based prediction and transcriptome-based prediction methods. The ab initio gene prediction was

conducted with Augustus (version 3.2.2), GeneMark-ET (version 4.29) and SNAP15 to predict coding genes. For the homology-based prediction, homologous proteins from several species (*Vitis vinifera*, *Prunus persica*, *Populus trichocarpa*, *Oryza sativa*, *Medicago truncatula*, *Glycine max*, *Citrus clementina*, *Theobroma cacao*, *Pyrus bretschneideri*) were downloaded from NCBI and aligned to the assembled genome. Then, Exonerate (version 2.47.3) [19] was used to generate gene structures based on the homology alignments. For the transcriptome-based prediction, transcriptome data were generated from mixed samples of flowers, buds, leaves, nuts and roots on the Illumina HiSeq 2500 platform (a total of 20.84 Gb raw data) and mapped to the genome assembly using TopHat (version 2.1.1). Cufflinks (version 2.1.1) (http://cufflinks.cbcb.umd.edu/) was then used to identify spliced transcripts in the gene models. All the gene evidence predicted by the above mentioned three approaches was integrated by EVidenceModeler (EVM version 1.1.1). Finally, a total of 36,479 protein-coding gene models were constructed (Table 1).

The obtained gene set was functionally analyzed using BLASTP with an E-value of $1e^{-5}$ against the NCBI-NR, Swiss-Prot, and euKaryotic Orthologous Groups (KOG) databases. Protein domains were annotated by mapping genes to the InterPro and Pfam databases using InterProScan [20] and HMMER [21]. Potential gene pathways were derived via gene mapping against the Kyoto Encyclopedia of Genes and Genomes (KEGG) databases, and Gene Ontology (GO) terms were extracted from the corresponding InterProScan or Pfam results (Fig. S2).

Quality assessment

To evaluate the completeness and coverage of the assembly, we aligned Illumina DNA and RNA reads to the *C. mollissima* assembly using BWA [22] and HISAT [23], respectively. The percentages of aligned DNA and RNA reads were 95.46 % and 97.41 %, respectively. In the core gene estimation using Benchmarking Universal Single-Copy Orthologs (BUSCO) [24], 1,392 of the 1,440 core genes (96.70 %) were found to be complete in the assembled genome, and 1,412 (complete BUSCOs and

fragmented BUSCOs) (98.10 %) of the 1,440 core genes had at least partial matches (Table S3). This result indicates that the assembly contains almost all genic regions, which further confirms the high quality of the *C. mollissima* genome assembly.

Physical map alignment

A total of 19,064 bacterial artificial chromosome (BAC) double-ended sequences from the previously published physical map [25] were aligned with the genome sequenced in the present study. Of these, 17,999 of the sequences were mapped onto our genome, accounting for 94.41 % of all BAC double-ended sequences. The reason that 1,065 (5.59 %) of the sequences did not map to the genome is most likely due to individual differences. The results also showed that 1,184 out of 1,300 contigs from the physical map could be mapped onto our genome (Table S4).

Gene family expansion and contraction

To understand the relationships of *C. mollissima* gene families to those of other plants, we performed a systematic comparison of genes among different species. The protein-coding genes of nine genomes, namely, *O. sativa* [26], *Malus domestica* [27], *P. trichocarpa* [28], *P. persica* [29], *C. mollissima*, *Q. robur* [30], *Fagus sylvatica* [31], *Juglans regia* [32] and *V. vinifera* [33], were used for the comparison. Gene loss and gain are among the primary reasons for functional changes. To gain greater insights into the evolutionary dynamics of the genes, we determined the expansion and contraction of the orthologous gene clusters in these eight species with CAFE software [34]. In the Chinese chestnut genome, a total of 17,422 gene families were identified, while 27,502 families of homologous genes were detected across the nine species. Of all the gene families (17,422), 209 were significantly expanded and 89 were contracted ($P < 0.05$) in *C. mollissima* (Fig. S3). The Venn diagram in Fig. 2a shows that 9,336 gene families were shared by the four species *C. mollissima*, *Q. robur*, *J. regia* and *F. sylvatica.* In addition, both specific and common gene families

were detected in these four species. A total of 11,952 genes and 8,884 gene families were found to be specific to Chinese chestnut (Table S5).

Phylogenetic analysis

To examine the evolutionary relationships of Chinese chestnut with other plants, we applied RAxML software (version 8.0.0; substitution model PROTGAMMAJTT, bootstrap value 100) [35] to perform a maximum likelihood genome-wide phylogenetic analysis of 540 single-copy genes from the nine plant genomes (Fig. 2b). The results support the hypothesis that Chinese chestnut and oak are sister groups. Based on the phylogeny and fossil record [5], we estimated the divergence time. The phylogenetic tree indicates that the orders Fagales and Rosales have a close genetic relationship, with a divergence time of 90.75 million years ago (Mya). The estimated divergence time of *C. mollissima* and *Q. robur* in the Fagales clade is approximately 13.62 Mya, while that of Chinese chestnut and *J. regia* is 62.7 Mya.

Long terminal repeat (LTR) insertion

In the final assembly, approximately 390 Mb of repetitive sequence was found, accounting for 49.69 % of the genome. LTR elements, accounting for 19.92 % of the genome of *C. mollissima*, are the most abundant transposable elements (Table S2). To estimate the insertion times of the LTR elements, we identified complete LTRs using a combination of *de novo* searches and manual inspection with LTR_Finder [36]. Finally, 5,470 complete LTRs were identified. We calculated the nucleotide distance for each of the 5,470 complete LTR elements using the molecular paleontology approach described by SanMiguel et al. [37] (Fig. 3 and Table S6). The average nucleotide distance of the LTR sequence pairs was 0.007681. When a substitution rate of 2.20×10^{-9} mutations per synonymous site per year was used, the insertion time distribution of the detected LTR elements indicated that the largest number of insertions occurred between 0 and 1.74 Mya [38].

Tandemly arrayed genes

Tandemly arrayed genes (TAGs) are gene clusters created by tandem duplication, and TAGs represent a large proportion of the genes in a genome [39]. To identify TAGs, we applied OrthoMCL with the default parameters to cluster genes into putative gene families. Subsequently, 1,122 TAGs were found by an in-house script; the duplicated genes were separated by less than 10 spacers (Fig. S4). These gene clusters contain 4,198 tandemly duplicated genes, accounting for 11.5 % of the total number of genes in *C. mollissima*, suggesting that a relatively high abundance of TAGs is a major feature of this genome. The TAGs of *C. mollissima* were compared with those of related species: *F. sylvatica* and *Q. robur* in the Fagaceae and *J. regia*, *M. domestica*, *P. persica* and *P. trichocarpa*. The percentage of TAGs in the complete genome of *C. mollissima* was markedly higher than those of *P. trichocarpa* (4.9 %) and *M. domestica* (4.2 %). The TAG percentage was also high in other Fagaceae species, such as *Q. robur* (19.7 %) and *F. sylvatica* (8.0 %). However, this trait was not shared with *J. regia*, another species closely related to *C. mollissima*, which has only 5.6 % TAGs. Furthermore, TAGs can also be highly abundant in non-Fagales species, such as *P. persica* (13.3 %) (Table S7). GO enrichment analysis of genes from the TAGs was performed using OmicShare Tools (https://omictools.com/). The results showed that these genes are enriched in the cell binding and catalytic activity pathways in the cellular component category (Fig. S5 and Table S8).

Conclusions

In this study, a high-quality annotated genome sequence of *C. mollissima* was obtained, similar to those of other Fagaceae species, and it was found to contain a relatively high proportion of tandemly repeated genes. The Chinese chestnut genome will serve as a reference genome and pave the way for future research involving comparative genomics, and studies on domestication, genetic improvement and breeding for disease resistance and nut quality in chestnuts.

Availability of supporting data

Additional files

Table S1: Statistics of clean data of *C.mollissima* for Illumina and PacBio sequencing

Table S2: Statistics of repeat elements for *C.mollissima* assembly using both RepeatModeler and RepeatMasker software

Table S3: Core gene estimation for *C.mollissima* assembly using BUSCO

Table S4: The alignment between the assembled genome and the physical map of *C.mollissima*

Table S5: Unique gene families of *C.mollissima* in four species

Table S6: Complete LTR elements in *C.mollissima*

Table S7: Numbers and proportions of TAGs in *C. mollissima* and other species

Table S8: Tandemly arrayed genes (TAGs) in *C.mollissima*

Figure S1: K-mer distribution of *C. mollissima*

Figure S2: GO term analysis for genes in *C. mollissima*

Figure S3: Analysis of the expanded and contracted gene families in *C. mollissima*.

Figure S4: Tandemly arrayed genes (TAGs) numbers in one cluster in *C. mollissima*

Figure S5: GO enrichment of genes from the TAGs in *C. mollissima*

Competing interests

The authors declare that they have no competing interests.

Authors' contributions

YX and LQ designed the project; YL, XN and GW collected samples and extracted the DNA samples; YX, QC, QZ, HL, ZZ and YS worked on sequencing and data analyzing; YX and YS wrote the manuscript; HH, KF, and TB revised the manuscript; QC and LQ read and approved the final version of the manuscript.

Acknowledgements

This work was supported by grants from the National Key Research & Development Program of China (2018YFD1000605); the National Natural Science Foundation of China (31870671; 31672135); the Project of Construction of Innovative Teams and Teacher Career Development for Universities and Colleges under Beijing Municipality (IDHT20180509); Supporting Plan for Cultivating High Level Teachers in Colleges and Universities in Beijing (CIT&TCD20180317).

References

1. Jaynes R. Chestnut. In: Moore, J. (Ed.) Advances in Fruit Breeding. Purdue University Press, West Lafayette, USA; 1975, pp 490-503.
2. Lang P, Dane F, Kubisiak TL, et al. Molecular evidence for an Asian origin and a unique westward migration of species in the genus *Castanea* via Europe to North America. Molecular Phylogenetics and Evolution 2007; **43** (1): 49-59. https://doi.org/10.1016/j.ympev.2006.07.022.
3. Martín MA, Herrera MA, and Martín LM. In situ conservation and landscape genetics in forest species. Journal of Natural Resources and Development 2012; **2** (3): 1-5. https://doi.org/10.5027/jnrd.v2i0.01.
4. Zhang YH, Liu L, Liang WJ, Zhang YM. China fruit monograph: Chinese chestnut and Chinese hazelnut volume. China Forestry Press, Beijing, China; 2005.
5. Hao FW, Zhang FR. Textual research on the cultivition histroy of *Castanea mollissima* in China. Ancient and Modern Agriculture 2014; **3**: 40-48.
6. FAO. Food and Agriculture Organization of the United Nations. FAOSTAT Statistics Database 2017. Available from: http://www.fao.org/faostat/en/#home. Accessed 01 Apr 2019.
7. Jacobs DF, Dalgleish HJ, Nelson CD. A conceptual framework for restoration of threatened plants: the effective model of American chestnut (*Castanea dentata*)

reintroduction. New Phytologist 2013; **197** (2): 378-393. https://doi:10.1111/nph.12020.

8. Hebard FV. The backcross breeding program of the American chestnut foundation. Journal of the American Chestnut Foundation 2006; **19**: 55-77.
9. Kremer A, Abbott AG, Carlson JE, et al. Genomics of Fagaceae. Tree Genetics & Genomes 2012; **8** (3): 583-610. https://doi.org/10.1007/s11295-012-0498-3.
10. Popkin G. Can a transgenic chestnut restore a forest icon?. Science 2018; **361** (6405): 830-831. https://doi:10.1126/science.361.6405.830.
11. Roane MK, Griffin GJ, Elkins JR. Chestnut blight, other *Endothia* diseases, and the genus *Endothia*. American Phytopathol Society Monograph Series, St. Paul, Minnesota, USA; 1986.
12. Barakat A, Staton M, Cheng CH, et al. Chestnut resistance to the blight disease: insights from transcriptome analysis. BMC Plant Biology 2012; **12** (1): 38. https://doi.org/10.1186/1471-2229-12-38.
13. Ji FY, Wei W, Liu Y, et al. Construction of a SNP-based high-density genetic map using genotyping by sequencing (GBS) and QTL analysis of nut traits in Chinese chestnut (*Castanea mollissima* Blume). Frontiers in Plant Science 2018; **9**: 816. https://doi.org/10.3389/fpls.2018.00816.
14. Zhang L, Lin Q, Feng YZ, et al. Transcriptomic identification and expression of starch and sucrose metabolism genes in the seeds of Chinese chestnut (*Castanea mollissima*). Journal of Agricultural and Food Chemistry 2015; **63** (3): 929-942. https://doi.org/10.1021/jf505247d.
15. Li M, Tian S, Jin L, et al. Genomic analyses identify distinct patterns of selection in domesticated pigs and Tibetan wild boars. Nature Genetics 2013; **45** (12): 1431-1438. https://doi.org/10.1038/ng.2811.
16. Zhang T, Hu Y, Jiang W, et al. Sequencing of allotetraploid cotton (*Gossypium hirsutum* L. acc. TM-1) provides a resource for fiber improvement. Nature Biotechnology 2015; **33** (5): 531-537. https://doi.org/10.1038/nbt.3207.

17. Marçais G, Kingsford C. A fast, lock-free approach for efficient parallel counting of occurrences of *k*-mers. Bioinformatics 2011; **27** (6): 764-770. https://doi.org/10.1093/bioinformatics/btr011.

18. Tarailo-Graovac M, Chen NS. Using RepeatMasker to identify repetitive elements in genomic sequences. Current Protocols in Bioinformatics 2009; **25**: 4.10.1-4.10.14. https://doi.org/10.1002/0471250953.bi0410s25.

19. Slater GSC and Birney E. Automated generation of heuristics for biological sequence comparison. BMC Bioinformatics 2005; **6**: 31. https://doi:10.1186/1471-2105-6-31.

20. Jones P, Binns D, Chang HY, et al. InterProScan 5: genome-scale protein function classification. Bioinformatics 2014; **30** (9): 1236-1240. https://doi.org/10.1093/bioinformatics/btu031.

21. Wheeler TJ, Eddy SR. nhmmer: DNA homology search with profile HMMs. Bioinformatics 2013; **29** (19): 2487-2489. https://doi.org/10.1093/bioinformatics/btt403.

22. Li H, Durbin R. Fast and accurate long-read alignment with Burrows-Wheeler transform. Bioinformatics 2010; **26** (5): 589-595. https://doi.org/10.1093/bioinformatics/btp698.

23. Kim D, Langmead B, Salzberg SL. HISAT: a fast spliced aligner with low memory requirements. Nature Methods 2015; **12** (4): 357-360. https://doi.org/10.1038/nmeth.3317.

24. Waterhouse RM, Seppey M, Simão FA, et al. BUSCO applications from quality assessments to gene prediction and phylogenomics. Molecular Biology and Evolution 2018; **35** (3): 543-548. https://doi.org/10.1093/molbev/msx319.

25. Fang GC, Blackmon BP, Staton ME, et al. A physical map of the Chinese chestnut (*Castanea mollissima*) genome and its integration with the genetic map. Tree Genetics & Genomes 2013; **9** (2): 525-537. https://doi.org/10.1007/s11295-012-0576-6.

26. International Rice Genome Sequencing Project. The map-based sequence of the rice genome. Nature 2005; **436** (7052): 793-800. https://doi.org/10.1038/nature03895.

27. Velasco R, Zharkikh A, Affourtit J, et al. The genome of the domesticated apple (*Malus* × *domestica* Borkh.). Nature Genetics 2010; **42** (10): 833-839. https://doi.org/10.1038/ng.654.

28. Tuskan GA, Difazio S, Jansson S, et al. The genome of black cottonwood, *Populus trichocarpa* (Torr. & Gray). Science 2006; **313** (5793): 1596-1604. https://doi.org/10.1126/science.1128691.

29. Verde I, Abbott AG, Scalabrin S, et al. The high-quality draft genome of peach (*Prunus persica*) identifies unique patterns of genetic diversity, domestication and genome evolution. Nature Genetics 2013; **45** (5): 487-494. https://doi.org/10.1038/ng.2586.

30. Plomion C, Aury JM, Amselem J, et al. Oak genome reveals facets of long lifespan. Nature Plants 2018; **4** (7): 440-452. https://doi.org/10.1038/s41477-018-0172-3.

31. Mishra B, Gupta DK, Pfenninger M, et al. A reference genome of the European beech (*Fagus sylvatica* L.). GigaScience 2018; **7** (6): 1-8. https://doi.org/10.1093/gigascience/giy063.

32. Martínez-García PJ, Crepeau MW, Puiu D, et al. The walnut (*Juglans regia*) genome sequence reveals diversity in genes coding for the biosynthesis of non-structural polyphenols. The Plant Journal 2016; **87** (5): 507-532. https://doi.org/10.1111/tpj.13207.

33. The French-Italian Public Consortium for Grapevine Genome Characterization. The grapevine genome sequence suggests ancestral hexaploidization in major angiosperm phyla. Nature 2007; **449** (7161): 463-467. https://doi.org/10.1038/nature06148.

34. De Bie T, Cristianini N, Demuth J, et al. CAFE: a computational tool for the study of gene family evolution. Bioinformatics 2006; **22** (10): 1269-1271. https://doi.org/10.1093/bioinformatics/btl097.

35. Stamatakis A. RAxML version 8: a tool for phylogenetic analysis and post-analysis of large phylogenies. Bioinformatics 2014; **30** (9): 1312-1313. https://doi.org/10.1093/bioinformatics/btu033.

36. Xu Z, Wang H. LTR_FINDER: an efficient tool for the prediction of full-length LTR retrotransposons. Nucleic Acids Research 2007; **35** (Web Server issue): W265-W268. https://doi.org/10.1093/nar/gkm286.

37. SanMiguel P, Gaut BS, Tikhonov A, et al. The paleontology of intergene retrotransposons of maize. Nature Genetics 1998; **20** (1): 43-45. https://doi.org/10.1038/1695.

38. Björn N, Nathaniel RS, Anna W, et al. The Norway spruce genome sequence and conifer genome evolution. Nature 2013, **497** (7451): 579-584. https://doi.org/10.1038/nature12211.

39. Pan D, Zhang LQ. Tandemly arrayed genes in vertebrate genomes. Comparative and Functional Genomics 2008; **2008**: 1-11. https://doi.org/10.1155/2008/545269.

 Click here to access/download;Table;Tables.doc

Table：

Table 1 Summary of *C.mollissima* genome assembly and gene model

**Genome assembly statistics**	
Total length	785,529,252 bp
Number of Contigs	2,707
Largest Contig Length	6,584,328 bp
N50 length (Contigs)	944,461 bp
N90 length (Contigs)	133,678 bp
Counts of N50 (Contigs)	235
Counts of N90 (Contigs)	1,024
**Gene model statistics**	
Gene number	36,479
Gene density (per 100 kb)	4.64
Gene average length	1,139.63 bp
Exon number per Gene	4.41
Exon average length	258.15 bp
Intron average length	1,156.91 bp
Genome GC percent	36.07 %
Exon GC percent	43.36 %

Figure:

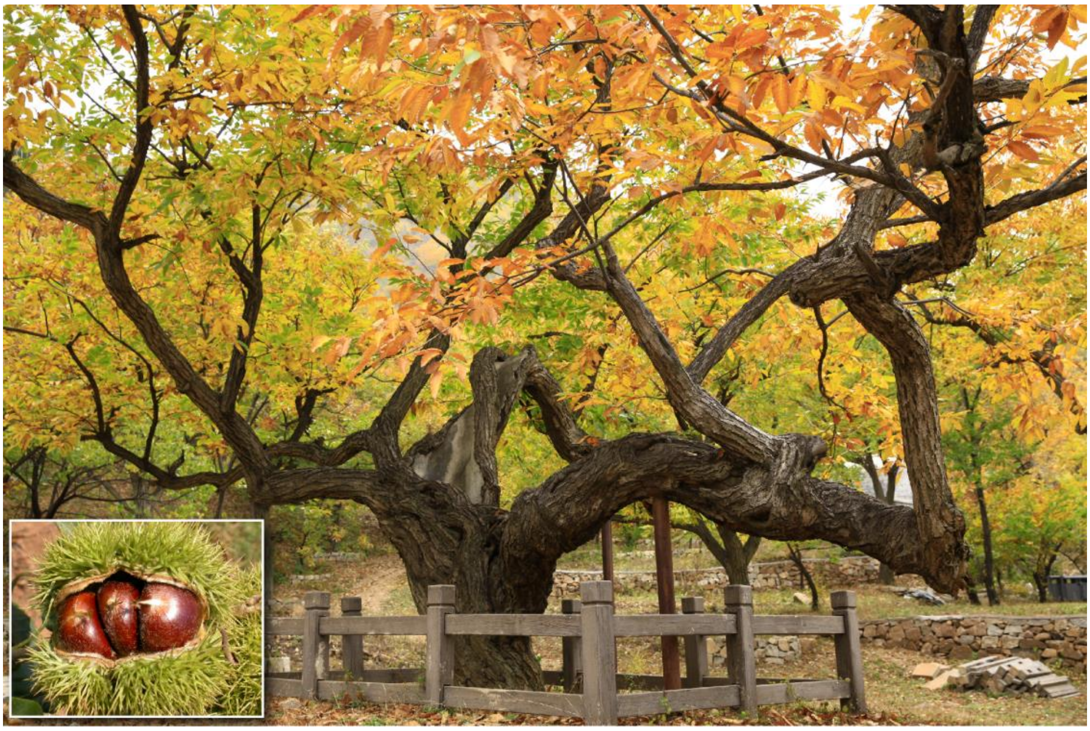

Figure 1 Example of Chinese chestnut tree (*C. mollissima*). Natural habitat of *C. mollissima* (image from the Water Great Wall, Beijing, China) and the nut of *C. mollissima* (image from Ling Qin) are showed.

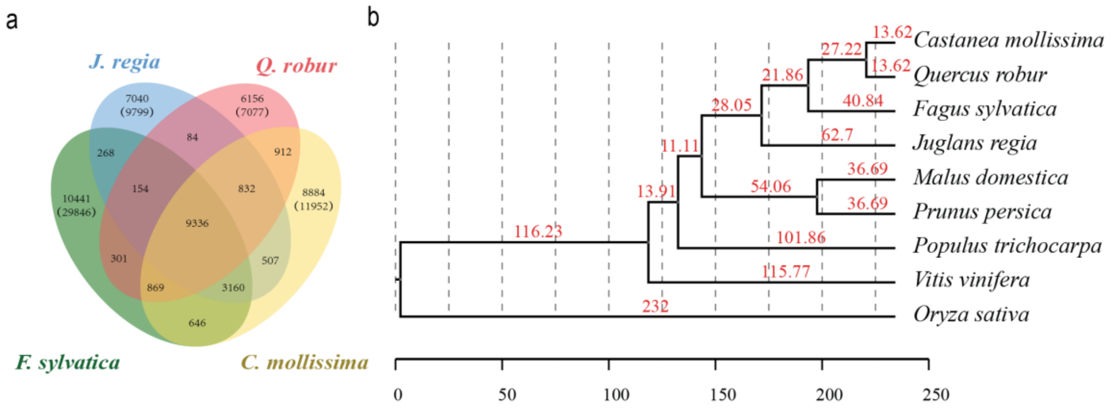

Figure 2 Phylogenetic relationships between Chinese chestnut and other species. A maximum-likelihood tree was obtained with 540 single copy orthologous genes. a) The shared and unique gene families in four closely related species are shown in the Venn diagram. Each number represents a number of gene families, and the number in brackets is a number of genes. b) The divergence times were estimated and are displayed on the phylogenetic tree.

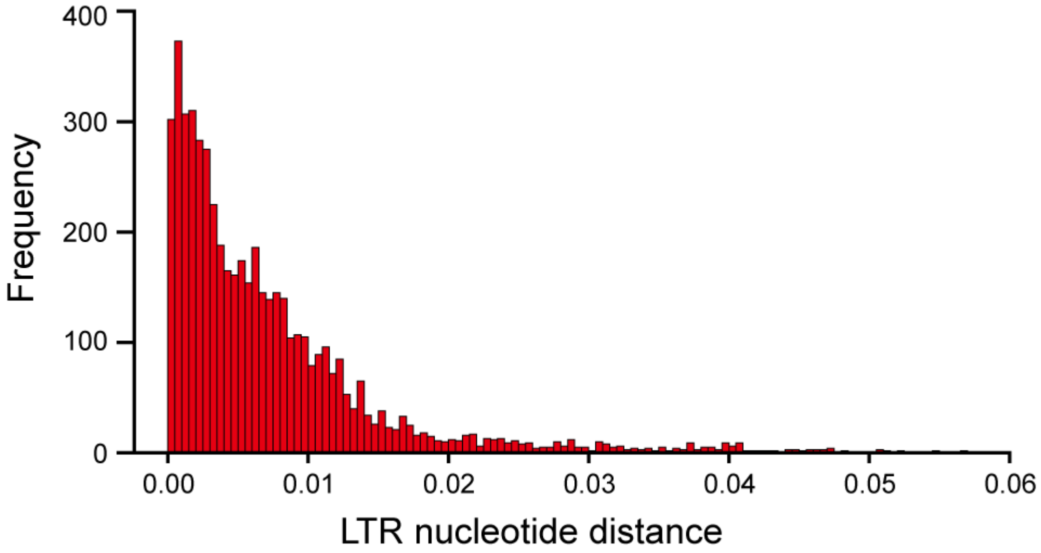

Figure 3 Nucleotide distance distribution of annotated LTR elements in *C.mollissima*.

Supplementary Figure S1-S5

Click here to access/download
Supplementary Material
Figure (S1-S5).doc

Supplementary Table S1-S3 and S7

Click here to access/download
Supplementary Material
Tables (S1-S3 and S7).doc

Supplementary Table S4

Click here to access/download
Supplementary Material
Table S4.xls

Supplementary Table S5

Click here to access/download
Supplementary Material
Table S5.xls

Supplementary Table S6

Click here to access/download
Supplementary Material
Table S6.xls

Supplementary Table S8

Click here to access/download
Supplementary Material
Table S8.xls

detailed protocols

Click here to access/download
Supplementary Material
Protocols.doc

Dear editors:

Thank you very much for your letter and for the reviewers' comments concerning our manuscript entitled "Hybrid *de novo* genome assembly of Chinese chestnut (*Castanea mollissima*)" (GIGA-D-18-00448).

The comments were all valuable and very helpful for revising and improving our paper, as well as providing guidance for our future research. We have studied the comments carefully and have made corrections that we hope will meet with approval. Attached please find the revised version, which we would like to submit for your kind consideration.

For the reviewers' convenience, we have already uploaded our raw data to the NCBI SRA database under BioProject # PRJNA527178 (SRA # of transcriptome: SRR8731962, SRA # of PacBio reads: SRR8755082-SRR8755097, and SRA # of Illumina reads: SRR8731963), and we have also confirmed that all the data are available now.

We would like to express our appreciation to you and the reviewers for your comments on our paper. We look forward to hearing from you.

Thank you and best regards.

Yours sincerely,
Ling Qin

Ling Qin, Prof.
Beijing University of Agriculture, Beijing, 102206, China
E-mail: qinlingbac@126.com

Response to Reviewers Click here to access/download;Personal Cover;Reviewers__comments.doc

Reviewer reports:

Reviewer 1:

The article describes the sequencing, assembly and annotation of the Chinese chestnut tree (*Castanea mollisima*) as well as some analysis based on gene families. My main concern about the manuscript is related to the availability of the sequencing data. Indeed the genomic and transcriptomic data are not available through SRA portal. On the other hand, the comparative analyses are not of equal quality. For example, the functional enrichment of specific gene families has not been inspected when compared to the TAGs. In addition, a few points need to be addressed.

A: For the reviewers' convenience, we have already uploaded our raw data to the NCBI SRA database; the BioProject # is PRJNA527178 (SRA # of transcriptome: SRR8731962, SRA # of PacBio reads: SRR8755082-SRR8755097, and SRA # of Illumina reads: SRR8731963), and we have also confirmed that all the data are available now.

Sampling and Sequencing:
- Line 31: Genome DNA instead of Genomic DNA.

A: Yes. We have revised it in the line 82 on page 4 of the revised manuscript.

- Line 39: approximately 54 Gb, where Table S1 reports 39 Gb.

A: The data in Table S1 are correct, and we have double-checked and verified the data in the revised manuscript and tables and corrected the typos in the revised manuscript.

- Line 49: In total 80 Gb, where Table S1 reports 69 Gb.

A: The data in Table S1 are correct, and we have double-checked and verified the data in the revised manuscript and tables and corrected the typos in the revised manuscript.

Genome size and heterozygosity estimation:
- The authors report a heterozygosity rate of approximately 0.17%, I think it is of interest to compare this rate with other trees, like beech or oak.

A: The genome size and heterozygosity rate were evaluated before we started the genome sequencing project, and the heterozygosity rate that we obtained was 0.17%. Based on our common sense and a comparison with data from other trees, we thought the results were unreasonable. Therefore, we double-checked the raw data and found some bacterial contamination in the Illumina raw data, which caused low coverage depth and resulted in inaccurate genome size and heterozygosity rate estimations. Therefore, we resequenced the samples without bacterial contamination using the Illumina HiSeq 2500 sequencer and then used the new data to perform the same analyses (genome size and heterozygosity estimation, genome assembly and error correction). However, the initial genome size and heterozygosity estimates (0.17%) from the previous report using the contaminated data were mistakenly used in the manuscript submitted for review. We have corrected these estimates in the revised manuscript, and the correct heterozygosity rate should be 0.87%. Comparing this estimate with those for beech (heterozygosity rate 0.19%) and oak (heterozygosity rate ~1%), we found that our result was more similar to oak.

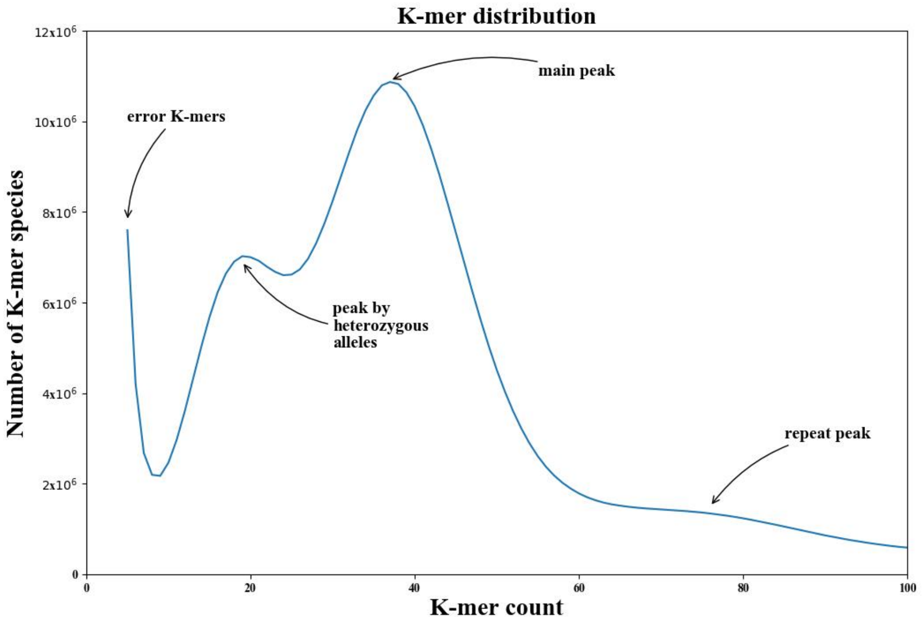

Figure S1 K-mer distribution of *C. mollissima*.

The frequency of each k-mer in raw sequencing reads was calculated for k=17.

Genome assembly and annotation:

- The authors describe the polishing step made using Quiver, but it is unclear how the several (how many?) rounds of iterative error correction, with short reads, were performed.

A : We performed six rounds of interactive error correction with the Illumina data and have added this information to line 111-113 on page 5 of the revised manuscript.

- The annotation process is not sufficiently described. The authors should describe which databases and which tools were used, and they should describe the transcriptomic dataset.

A: In the revised manuscript, we describe the annotation process in detail. In addition, we describe a transcriptomic dataset, which has been uploaded to the SRA database. We validated the exon/intron boundaries using transcripts and the GU-AG rule. We identified 36,479 protein-coding genes in the *C. mollissima* genome. In detail, 47,666 genes were predicted through the *de novo* method, 26,647 genes were annotated based on RNA transcripts or raw RNA reads, and 14,695 genes were supported by homology. We also listed the gene numbers that could be annotated in the different databases. For your convenience, we listed the gene numbers that we used and integrated to predict our protein-coding genes in the following table.

Table Gene prediction and annotation

	RNA-Seq data-based	Ab initio	Homology -based	Integration	Annotation				
					NR	GO	Swiss-Prot	KEGG	KOG
Number of predicted genes	26,647	47,666	14,695	36,479	36,232	23,672	26,665	27,584	35,375
Tools	Cufflinks	Braker	Exonerate	EVM	Blast				

Quality assessment:

- line 28: genetic regions instead of genic regions.

A: Yes. We have revised it in the line 152 on page 7 of the revised manuscript.

Gene family expansion and contraction
- A total of 956 and 471 gene families were found to be specific to Chinese chestnut and oak. However, on Figure 2a that refers to gene families, the numbers are different. The authors should clarify the text and/or the legend of Figure 2.

A: We have corrected these numbers in the revised manuscript. In addition, in accordance with the reviewers' suggestions, we adjusted the text and the legend of Figure 2a to increase clarity, and we recreated the Venn diagram using *C. mollissima*, *Q. robur*, *J. regia* and *F. sylvatica*. Please see the "Gene family expansion and contraction" section (lines 163-178, pages 7-8) of the revised manuscript.

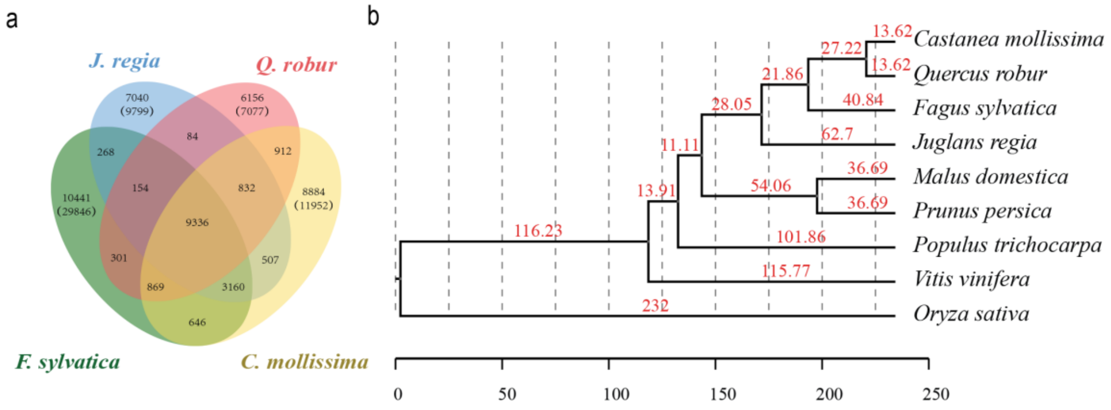

Figure 2 Phylogenetic relationships between Chinese chestnut and other species. A maximum-likelihood tree was obtained with 540 single copy orthologous genes. a) The shared and unique gene families in four closely related species are shown in the Venn diagram. Each number represents a number of gene families, and the number in brackets is a number of genes. b) The divergence times were estimated and are displayed on the phylogenetic tree.

Tandemly arrayed genes
- The authors claim that "TAGs are gene clusters created by tandem duplication, and TAGs represent a large proportion of the genes in a genome". Authors should add citations to support this sentence.

A: We have cited a reference to support this sentence.
Pan D, Zhang LQ. Tandemly arrayed genes in vertebrate genomes. Comparative and functional genomics 2008; **2008**: 1-11. https://doi.org/10.1155/2008/545269.

- The authors suggest that an abundance of TAGs is a major feature if the genome, but they should compare to other species, such as the oak genome which contains a high proportion of TAGs (as shown in Plomion et al, 2018).

A: According to this suggestion, we compared the number and proportion of TAGs in different species and added them to the revised manuscript as supplementary Table S7. Please see the "Tandemly arrayed genes" section (lines 204-222, page 9) of the revised manuscript.

Table S7 Numbers and proportions of TAGs in *C. mollissima* and other species

	C. mollissima	*F. sylvatica*	*Q. robur*	*P. trichocarpa*	*J. regia*	*M. domestica*	*P. persica*
Gene cluster number	1,112	1,304	1,467	636	696	673	906
Gene number	4,198	4,989	5,099	2,263	2,054	2,008	3,027
Percentage (%)	11.5	8.0	19.7	4.9	5.6	4.2	13.3

Reviewer2: First of all, I congratulate the authors on there effort and the valuable results obtained. The genome seems mostly well assembled, but there are some issues as outlined below that should be addressed.

The English is of some concern. There are only few grammar and spelling mistakes, but there are many sentences that are phrased in an odd manner, sometimes there is also highly unusual word usage. The authors are thus strongly encouraged to have their manuscript proofread by a native English speaker.

A: The revised manuscript has been polished by a senior editor at American Journal Experts (https://secure.aje.com, contract #6G58F9VV) and proofread by Professor Rene Geurts, from Department of Molecular Biology, Wageningen University and Professor Jocelyn Rose, from School of Integrative Plant Science, Cornell University.

Title. The title should mention that a hybrid approach was used.

A: Yes. We have revised the title as follows: “Hybrid *de novo* genome assembly of Chinese chestnut (*Castanea mollissima*)”.

Page 5
Line 24. *Castanea sativa* has dominant role as forest tree only in few areas of Europe (e.g. some parts of northern Italy and southern France).
Lines 42-44. A citation should be given for this claim.
Lines 51-57. A citation should be given for this claim.

A: Yes. We have revised these passages and cited the references in the manuscript in lines 50-51, 55-58, and 60-64 on page 3 of the revised manuscript.

Page 6
Line 9. The available resources should be cited here, e.g. the genome published previously.

A: Yes. We have cited the references in the manuscript in lines 69-72 on page 4 of the revised manuscript.

Line 28. The sequenced indivual(s) should be described in more detail and the sampling date should be given.

A: Yes. We have described the sampling information in more detail in lines 77-83 on page 4 of the revised manuscript.

Genome assembly. As SMARTdenovo does not have a correction step, all data should have been corrected before assembly. At 100x, it might have been useful doing self-correction rather than Illumina-based filtering (first).

A: We assembled the genome using three software packages. Interestingly, the results indicated that the genome assembly performed with SMARTdenovo displayed higher continuity than those from the other two software packages. To achieve a high-accuracy genome assembly, the assembled sequence was then polished using Quiver (SMRT Analysis v2.3.0) with the default parameters, and six rounds of iterative error correction were performed using the Illumina clean reads.

**Species**	**Assembly Method**	**Length of Scaffolds (bp)**	**Number of Scaffolds**	**N50 (bp)**	**N90 (bp)**	**GC Content (%)**	**N Content (%)**
C. mollissima	SMARTdenovo	785,529,252	2,707	944,461	133,678	35.20	0.0
C. mollissima	Wtdbg	717,241,349	5,544	414,170	70,843	35.15	0.0
C. mollissima	Falcon	752,555,967	8,334	196,059	36,016	35.19	0.0

Page 7
Lines 46-49. More detail needs to be provided here. From the information given, it is unclear how gene predictions were actually done.

A: Yes. We have added a detailed description to the “Genome assembly and annotation” section (lines 107-143, pages 5-6) of the revised manuscript.

Line 51. The output from the evidence modeler could be checked against transcripts to vailidate exon/intron boundaries.

A: We validated the exon/intron boundaries using transcripts and the GU-AG rule. We identified 36,479 protein-coding genes in the *C. mollissima* genome. In detail, 47,666 genes were predicted through the *de novo* method, 26,647 genes were annotated by RNA transcripts or raw RNA reads, and 14,695 genes were supported by homology. We also listed the numbers of genes that could be annotated with the different databases. For your convenience, we listed the gene numbers that we used and integrated to predict the protein-coding genes in the following table.

Table Gene prediction and annotation

	RNA-Seq data-based	Ab initio	Homology -based	Integration	Annotation				
					NR	GO	Swiss-Prot	KEGG	KOG
Number of predicted genes	26,647	47,666	14,695	36,479	36,232	23,672	26,665	27,584	35,375
Tools	Cufflinks	Braker	Exonerate	EVM	Blast				

Page 8
Line 28. Most?

A: We revised the manuscript to improve clarity in line 152 on page 7.

Page 9
Gene family expansion and contraction/phylogenetic analysis. For both of these analyses, the genome of Fagus sylvatica should be included as well as an important reference point in Fagaceae. In the Venn diagram, it could replace Malus domestica, which is largely unrelated to chestnut and oak.

A: Yes. We have corrected this issue in the revised manuscript (lines 163-178, pages 7-8). We recreated the Venn diagram using *C. mollissima*, *Q. robur*, *J. regia* and *F. sylvatica*. In addition, in accordance with the reviewers’ suggestions, we adjusted the text and the legend of Figure 2a to improve clarity.

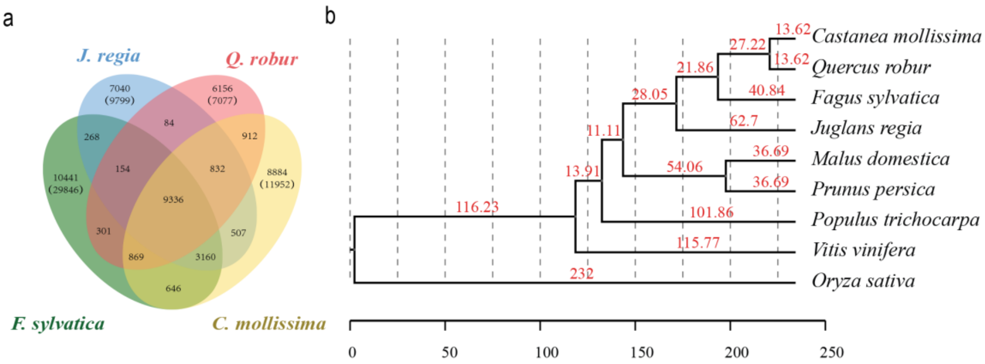

Figure 2 Phylogenetic relationships between Chinese chestnut and other plants. A maximum-likelihood tree was obtained with 540 single copy orthologous genes. a) The shared and unique gene families in four closely related species are shown in the Venn diagram. Each number represents a number of gene families, and the number in brackets is a number of genes. b) The divergence times were estimated and are displayed on the phylogenetic tree.

Line 48. RAxML is a program not a method. It should be mentioned, which version and which substitution model were used, as well as the amount of bootstrap replicated done.

A: Yes. We have revised this text in lines 181-182 on page 8 of the revised manuscript.

Line 57. It should be mentioned where these divergence times are coming from. the authors could also do their own estimations using a dating software.

A: We estimated these divergence times using r8s. We selected three time scales from the time tree (http://www.timetree.org/). We also revised the manuscript to improve clarity.

Page 10
Tandemly arranged genes. This should be specifically compared to Quercus, Fagus and a species outside Fagaceae, such as walnut and poplar, to investigate, how special this trait is.

A: The tandemly arranged genes of *C. mollissima* were compared with those of closely related species in the genomes of *F. sylvatica* and *Q. robur* in the Fagaceae and the additional genomes of *J. regia*, *M. domestica*, *P. persica* and *P. trichocarpa*. The results are indicated in the "Tandemly arrayed genes" section (lines 204-222, page 9) of the manuscript and have also been added to the supplementary information as supplementary Table S7.

Table S7 Numbers and proportions of TAGs in *C. mollissima* and other species

	C. mollissima	*F. sylvatica*	*Q. robur*	*P. trichocarpa*	*J. regia*	*M. domestica*	*P. persica*
Gene cluster number	1,112	1,304	1,467	636	696	673	906
Gene number	4,198	4,989	5,099	2,263	2,054	2,008	3,027
Percentage (%)	11.5	8.0	19.7	4.9	5.6	4.2	13.3
