# Supplementary material for: Hybrid de novo genome assembly of Chinese chestnut (Castanea mollissima)
Source: Gigascience. 2019 Sep 12;8(9):giz112. doi: 10.1093/gigascience/giz112 (PMC6741814; doi:10.1093/gigascience/giz112)
Supplement: giz112_GIGA-D-18-00448_Revision_2 [file giz112_giga-d-18-00448_revision_2.pdf]

GigaScience

Hybrid de novo genome assembly of Chinese chestnut (Castanea mollissima)

--Manuscript Draft--

Manuscript Number:	GIGA-D-18-00448R2
Full Title:	Hybrid de novo genome assembly of Chinese chestnut (Castanea mollissima)
Article Type:	Data Note
Funding Information:	National Key Research & Development Program of China (2018YFD1000605) — Dr LING QIN<br>National Natural Science Foundation of China (31870671) — Dr LING QIN<br>National Natural Science Foundation of China (31672135) — Dr Qingqin Cao<br>Project of Construction of Innovative Teams and Teacher Career Development for Universities and Colleges under Beijing Municipality (IDHT20180509) — Dr LING QIN<br>Supporting Plan for Cultivating High Level Teachers in Colleges and Universities in Beijing (CIT&TCD20180317) — Dr Qingqin Cao
Abstract:	Background: Castanea mollissima is widely cultivated in China for nut production. This plant also plays an important ecological role in afforestation and ecosystem services. To facilitate and expand the utilization of C. mollissima for breeding and its genetic improvement, we report here the whole genome sequence of C. mollissima. Findings: We produced a high-quality assembly of the C. mollissima genome using PacBio single-molecule sequencing. The final draft genome is approximately 785.53 Mb long, with a contig N50 size of 944 kb, and we further annotated 36,479 protein-coding genes in the genome. Phylogenetic analysis showed that C. mollissima diverged from Quercus robur, a member of the Fagaceae family, approximately 13.62 million years ago. Conclusions: The high-quality whole genome assembly of C. mollissima will be a valuable resource for further genetic improvement and breeding for disease resistance and nut quality.<br>Keywords: Castanea mollissima; genome assembly; annotation; evolution
Corresponding Author:	LING QIN<br><br>CHINA
Corresponding Author Secondary Information:	
Corresponding Author's Institution:	
Corresponding Author's Secondary Institution:	
First Author:	Yu Xing
First Author Secondary Information:	
Order of Authors:	Yu Xing<br>Yang Liu<br>Qing Zhang<br>Xinghua Nie<br>Yamin Sun<br>Zhiyong Zhang

Powered by Editorial Manager® and ProduXion Manager® from Aries Systems Corporation

	Huchen Li
	Kefeng Fang
	Guangpeng Wang
	Hongwen Huang
	Ton Bisseling
	Qingqin Cao
	LING QIN
**Order of Authors Secondary Information:**	
**Response to Reviewers:**	Dear editor,<br>After reading carefully the reviewers’ comments, we tried our best to answer all the questions point-by-point. As you know, the Chinese chestnut genome has been preprinted on bioRxiv. We compared the assembly quality of our genome with theirs, as shown in Table S1, and all the data support our genome assembly quality is better than them. We also added those comparison results in the part of “background information”. Because only Assembly V1.1 of Staton et al. 2019 is released on the website of hardwood genomics (https://www.hardwoodgenomics.org/), so we also downloaded and compared the genome assembly quality with the Assembly V1.1 of Staton et al. 2019, individually. The results were listed in the Table S1, Table S11 and Table S12 below. As your requirement, we have also uploaded all the gff files, custom scripts and other intermediate and processed resources on the ftp.<br><br>Reviewer reports:<br><br>Reviewer #1: The authors present an improved manuscript "Hybrid de novo genome assembly of Chinese chestnut (Castanea mollissima) " and my major compulsory revisions were taken into account. The authors compare the heterozygosity rate of C. mollissima with other Fagaceae species, and I think they should add these results to a supplementary table and how they were obtained in the main manuscript (section Genome size and heterozygosity estimation).<br><br>Answer: The size of the C. mollissima genome was estimated to be approximately 772 Mb, and the heterozygosity level of C. mollissima was approximately 0.87 % (Table S2). Comparing this estimate with those for beech and oak, we found that our result was more similar to European beech. The heterozygosity rate of oak is from Ramos AM et al., 2018 [22]. In the genome literature of European beech [36], the heterozygosity rate is not mentioned clearly. In order to compare the heterozygosity rate in three species, we download the PRJEB24056 (ERX2326485 and EXR2326486) from NBCI database and estimated the heterozygosity rate is approximately 0.63% based on k-mers method.<br><br>Reviewer #2: The manuscript has been significantly improved over the previous version, even though I feel that literature on the subject is still not fully covered. For example, Staton et al. 2015 https://doi.org/10.1186/s12864-015-1942-1 is not mentioned, and no attempts seem to have been made to compare the results of this study to those generated by other authors on the same tree species over the past 5 years.<br><br>Answer: Thank you for the suggestion and we have addressed the relevant published literatures of chestnut over the past five years and also added some comparison with our results in the “Background information” and “Results” sections.<br><br>line 86 - The sonication device used should be mentioned, as well as the parameters used for fragmentation.<br><br>Answer: The DNA was sheared by Covaris S2 system (Covaris, USA) for short insert paired-end (PE) library construction. The shearing conditions were as the following: the number of cycles is 2 and shearing time is 40 seconds per cycle.<br><br>lines 109-115 - I am still not fully convinced by this strategy. A data cleanup before the

Powered by Editorial Manager® and ProduXion Manager® from Aries Systems Corporation

	assembly might have been better. In any case, it should be mentioned, how sequence data of potentially contaminant organisms were searched for and the results of the searches should be presented.<br><br>Answer: Thank you for the reviewer’s suggestion. Actually, we did data cleanup before the assembly. The raw data have been filtered and trimmed. Illumina data quality control settings are as follows: SLIDINGWINDOW: 4: 15 MINLEN: 50 using software as Trimmomatic. Pacbio data quality control standard is RQ>0.75 and the minimum subreads length is 500 bp using software SMRT Link 6.0. The Table S2 is added to show the comparison between Polymerase reads and Subreads from PacBio sequencing. The detailed method for data quality control is added in the protocol.<br>To avoid potential contaminant, we carried out the contaminant check on the raw data of PacBio sequencing. The detailed method is as follows: 1000 subreads sequences are randomly selected to search against the NT database using BLAST (threshold settings -F F -e 1e-5). If a subread shows the highest alignment with sequences from non-plant species, it is identified as contaminant. In our results, 216 in 1000 subreads are blasted against the NT database and all the 216 subreads are belong to Viridiplantae. No contaminant read is detected in randomly selected 1000 subreads. We also blasted all the 36,479 protein-coding genes in our genome against the NT database. 35,544 genes are blasted and 35,353 genes are classified into Viridiplantae. The results have been uploaded as intermediate files to the ftp.<br><br><br>lines 136-138 - Details regarding how the gene boundaries from the Evidence Modeler output were verified are missing.<br><br>Answer: The In-house script was used to scan and count the initial codon, termination codon, exon and intron boundaries of each gene. The results and scripts were provided in the intermediate files. We have uploaded the intermediate files to the ftp.<br><br>line 210 - The script needs to be made accessable in order to make the results reproducible.<br><br>Answer: All the scripts were provided in the intermediate files and uploaded to the ftp.
**Additional Information:**	
**Question**	**Response**
Are you submitting this manuscript to a special series or article collection?	No
**Experimental design and statistics**<br><br>Full details of the experimental design and statistical methods used should be given in the Methods section, as detailed in our Minimum Standards Reporting Checklist. Information essential to interpreting the data presented should be made available in the figure legends.<br><br>Have you included all the information requested in your manuscript?	Yes
**Resources**	Yes

A description of all resources used, including antibodies, cell lines, animals and software tools, with enough information to allow them to be uniquely identified, should be included in the Methods section. Authors are strongly encouraged to cite Research Resource Identifiers (RRIDs) for antibodies, model organisms and tools, where possible.<br><br>Have you included the information requested as detailed in our Minimum Standards Reporting Checklist?	
**Availability of data and materials**<br><br>All datasets and code on which the conclusions of the paper rely must be either included in your submission or deposited in publicly available repositories (where available and ethically appropriate), referencing such data using a unique identifier in the references and in the "Availability of Data and Materials" section of your manuscript.<br><br>Have you have met the above requirement as detailed in our Minimum Standards Reporting Checklist?	Yes

Powered by Editorial Manager® and ProduXion Manager® from Aries Systems Corporation

Revised Manuscript

Click here to view linked References

Click here to access/download;Manuscript;Revised manuscript.doc

Hybrid *de novo* genome assembly of Chinese chestnut (*Castanea mollissima*)

Yu Xing[1,2†], Yang Liu[2†], Qing Zhang[2†], Xinghua Nie[2], Yamin Sun[3], Zhiyong Zhang[1,2], Huchen Li[1,7], Kefeng Fang[4], Guangpeng Wang[5], Hongwen Huang[6], Ton Bisseling[1,7], Qingqin Cao[1,8*], Ling Qin[1,2*]

[1] Beijing Advanced Innovation Center for Tree Breeding by Molecular Design, Beijing University of Agriculture, Beijing, 102206, China

[2] College of Plant Science and Technology, Beijing Key Laboratory for Agricultural Application and New Technique, Beijing University of Agriculture, Beijing, 102206, China

[3] Research Center for Functional Genomics and Biochip, Tianjin, 300457, China

[4] College of Landscape Architecture, Beijing Collaborative Innovation Center for Eco-Environmental Improvement with Forestry and Fruit Trees, Beijing University of Agriculture, Beijing, 102206, China

[5] Changli Institute of Pomology, Hebei Academy of Agriculture and Forestry Sciences, Changli, 066600, China

[6] South China Botanical Garden, Chinese Academy of Sciences, Guangzhou, 510650, China

[7] Laboratory of Molecular Biology, Department of Plant Sciences, Wageningen University, Wageningen, 6708 PB, The Netherlands

[8] College of Biological Science and Engineering, Key Laboratory of Urban Agriculture (North China) Ministry of Agriculture, Beijing University of Agriculture, Beijing, 102206, China

[†] These authors contributed equally to this work.

* To whom correspondence should be addressed.

E-mails: caoqingqin@bua.edu.cn (Q.C) and qinlingbac@126.com(L.Q)

Abstract

Background: *Castanea mollissima* is widely cultivated in China for nut production. This plant also plays an important ecological role in afforestation and ecosystem services. To facilitate and expand the utilization of *C. mollissima* for breeding and its genetic improvement, we report here the whole genome sequence of *C. mollissima*. **Findings:** We produced a high-quality assembly of the *C. mollissima* genome using PacBio single-molecule sequencing. The final draft genome is approximately 785.53 Mb long, with a contig N50 size of 944 kb, and we further annotated 36,479 protein-coding genes in the genome. Phylogenetic analysis showed that *C. mollissima* diverged from *Quercus robur*, a member of the Fagaceae family, approximately 13.62 million years ago. **Conclusions:** The high-quality whole genome assembly of *C. mollissima* will be a valuable resource for further genetic improvement and breeding for disease resistance and nut quality.

Keywords: *Castanea mollissima*; genome assembly; annotation; evolution

Data Description

Background information

Castanea, a genus of the Fagaceae family, occurs naturally throughout the forests of eastern North America, Europe and Asia, where it is ecologically and economically important. *Castanea* contains seven species. Chinese chestnut (*C. mollissima*), Chinese seguin (*C. seguinii*), Chinese chinkapin (*C. henryi*) and Japanese chestnut (*C. crenata*) occur in East Asia and show high genetic diversity [1]. The American chestnut (*C. dentata*) and chinkapin (*C. pumila*) occur only in North America, while the European chestnut (*C. sativa*) distributes in Europe, and they are the predominant tree species in the deciduous forests of eastern North America and some parts of northern Italy and southern France [2]. Chestnuts are important forest resources that provide wood products and food, and they are also keystone species due to their ecological roles in afforestation and ecosystem services [3].

Chinese chestnut is geographically widespread and is cultivated in 26 Chinese provinces for commercial nut production [4]. China is rich in diverse germplasm resources of Chinese chestnut, and the cultivation of Chinese chestnut has a long history, which spans over 6000 years, according to archeological discoveries in the Banpo Ruins of Xi'an, China [5]. The annual nut yield of Chinese chestnut is high. In 2017, Chinese chestnut production was 1,939,719 tonnes, accounting for 83.34 % of the world's total chestnut production that year [6]. Due to its high nut quality, easily peeled pellicle, excellent adaptability to infertile soil, and natural resistance to diseases, Chinese chestnut has been broadly used in breeding programs, especially to introduce resistance to the chestnut blight fungal pathogen (*Cryphonectria parasitica*) in the United States [7, 8]. An accidental introduction of the chestnut blight fungus at the beginning of the 20th century destroyed 4 billion American chestnuts, which were a predominant forest tree species, by 1950 [9, 10, 11]. Three quantitative trait locis (QTLs) of blight disease were verified in the F_2 mapping population of interspecies of *C. mollissima* × *C. dentata* and two of them shared synteny with two QTLs of

powdery mildew resistance in peach [12, 13]. Recently, two QTLs were also identified for link disease (*Phytophthora cinnamomi*) resistance and the QTL located in linkage group E is consistent with a previous preliminary study developed in the populations of *C. mollissima* × *C. dentata* [14]. All those evidences suggesting that Chinese chestnut has substantial levels of resistance to chestnut blight and has been utilized as a resistance resource to restore American chestnut [7].

Due to the considerable economic and ecological importance of Chinese chestnut in the *Castanea* genus, a Project of NSF Fagaceae Genomic Tools has been launched and a genome sequence data derived from Roche 454 platform and Sanger sequence data (V1.1) was produced in 2013 and released in 2014 at the Hardwood Genomics website. Recently, a Chinese chestnut genome was preprinted online on bioRxiv [15]. The assembly quality of two genomes were compared in Table S1. A high-quality whole genome information available for this species is urgent needed, which plays a key role on molecular studies of the major traits involved in nut quality and disease resistance [16, 17, 18]. In this study, we report a high-quality whole genome sequence of *C. mollissima*. This research allows for a better understanding of the evolution of *Castanea* and produces fundamental information to facilitate and expand comparative genomic studies, domestication, breeding and genetic improvement.

Sampling and sequencing

A mature, healthy tree of wild *C. mollissima* was chosen from the Zhangcunping national forest reserve (31°16’49.25” N, 111°08’25.40” E, 1261 m altitude) of the city of Yichang in Hubei Province, China. The individual measured ~ 12 m in height, and its trunk was ~10 cm in diameter (at breast height). Fresh leaves were collected on 18 June 2017. The samples were immediately frozen in liquid nitrogen and then stored at -80 ℃. The genomic DNA of *C. mollissima* was extracted using the DNeasy Plant Mini Kit (Qiagen, Hilden, Germany) and used for sequencing (Fig. 1). The DNA was sheared by Covaris S2 system (Covaris, USA) for short-insert paired-end (PE) library construction. The shearing conditions were as the following: the number of cycles is 2

and shearing time is 40 seconds per cycle. Short-insert libraries with a size of 500 bp were constructed according to the instructions described in the Illumina library preparation kit (Illumina, CA, USA). All libraries were sequenced on an Illumina HiSeq 2500 sequencer with the PE 2×150 bp protocol. The raw data have been filtered and trimmed. Illumina data quality control settings are as follows: SLIDINGWINDOW: 4: 15 MINLEN: 50 using software as Trimmomatic. In total of approximately 34 Gb of clean data were generated, yielding a sequencing depth of ~42.7 X. For PacBio library construction, the genomic DNA of *C. mollissima* was sheared to 20 kb, and fragments shorter than 7 kb were filtered using BluePippin (Sage Science, MA, USA). The filtered DNA was then used to prepare a proprietary SMRTbell library using the PacBio DNA Template Preparation Kit (Pacific Biosciences, CA, USA). Pacbio data quality control standard is RQ>0.75 and the minimum subreads length is 500 bp using software SMRT Link 6.0. In total, ~69 Gb of quality-filtered data were obtained from PacBio sequencing with an average read length of 7,170 bp and a sequencing depth of ~87 X (Table S2).

Genome size and heterozygosity estimation

The distribution of short subsequence (k-mer) frequency, also known as the k-mer spectrum, is widely used to estimate genome size [19, 20]. A k-mer depth distribution was obtained from a Jellyfish [21] analysis, and the peak depth was clearly observed from the distribution data. The genome size was calculated with the following formula: genome size = total_k-mer_num/k-mer_depth (total_k-mer_num is the total number of k-mers from all reads, and k-mer_depth is the peak depth). Based on this method, the size of the *C. mollissima* genome was estimated to be approximately 772 Mb, and the heterozygosity level of *C. mollissima* was approximately 0.87 % (Fig. S1). Comparing this estimate with those of beech and oak, we found that our result was more similar to European beech (Table S3) [22].

Genome assembly and annotation

All of the subreads from PacBio sequencing were assembled using SMARTdenovo software with default values for all parameters except for -J, which was set to a value of 4000 (-J 4000 filters all reads with lengths less than 4,000 bp) (https://github.com/ruanjue/smartdenovo). The assembled sequence was then polished using Quiver (SMRT Analysis version 2.3.0) with the default parameters. To achieve a high-accuracy genome assembly, six rounds of iterative error correction were performed using the clean Illumina data. In total, 785.53 Mb of final assembly was obtained after correction using PacBio and Illumina PE read sequences, and the assembly comprised 2,707 contigs (N50 = 944 kb, N90 = 133 kb) (Table 1). Both RepeatModeler and RepeatMasker [23] were used for the *de novo* identification and masking of repeats. To ensure the integrity of genes in the subsequent analyses, low-complexity regions or simple repeats were not masked because some of these sequences could be within genes. Finally, 49.69 % of the assembled bases were masked (Table S4). Protein-coding region identification and gene prediction were performed through a combination of ab initio prediction, homology-based prediction and transcriptome-based prediction methods. The ab initio gene prediction was conducted with Augustus (version 3.2.2), GeneMark-ET (version 4.29) and SNAP15 to predict coding genes. For the homology-based prediction, homologous proteins from several species (*Vitis vinifera*, *Prunus persica*, *Populus trichocarpa*, *Oryza sativa*, *Medicago truncatula*, *Glycine max*, *Citrus clementina*, *Theobroma cacao*, *Pyrus bretschneideri*) were downloaded from NCBI and aligned to the assembled genome. Then, Exonerate (version 2.47.3) [24] was used to generate gene structures based on the homology alignments. For the transcriptome-based prediction, transcriptome data were generated from mixed samples of flowers, buds, leaves, nuts and roots on the Illumina HiSeq 2500 platform (a total of 20.84 Gb raw data) and mapped to the genome assembly using TopHat (version 2.1.1). Cufflinks (version 2.1.1) (http://cufflinks.cbcb.umd.edu/) was then used to identify spliced transcripts in the gene models. All the gene evidence predicted by the above mentioned three

approaches was integrated by EVidenceModeler (EVM version 1.1.1). Finally, a total of 36,479 protein-coding gene models were constructed (Table 1).

The obtained gene set was functionally analyzed using BLASTP with an E-value of 1e^{-5} against the NCBI-NR, Swiss-Prot, and euKaryotic Orthologous Groups (KOG) databases. Protein domains were annotated by mapping genes to the InterPro and Pfam databases using InterProScan [25] and HMMER [26]. Potential gene pathways were derived via gene mapping against the Kyoto Encyclopedia of Genes and Genomes (KEGG) databases, and Gene Ontology (GO) terms were extracted from the corresponding InterProScan or Pfam results (Fig. S2).

Quality assessment

To evaluate the completeness and coverage of the assembly, we aligned Illumina DNA and RNA reads to the *C. mollissima* assembly using BWA [27] and HISAT [28], respectively. The percentages of aligned DNA and RNA reads were 95.46 % and 97.41 %, respectively. In the core gene estimation using Benchmarking Universal Single-Copy Orthologs (BUSCO) [29], 1,392 of the 1,440 core genes (96.70 %) were found to be complete in the assembled genome, and 1,412 (complete BUSCOs and fragmented BUSCOs) (98.10 %) of the 1,440 core genes had at least partial matches (Table S5). This result indicates that the assembly contains almost all genic regions, which further confirms the high quality of the *C. mollissima* genome assembly.

Physical map alignment

A total of 19,064 bacterial artificial chromosome (BAC) double-ended sequences from the previously published physical map [30] were aligned with the genome sequenced in the present study. Of these, 17,999 of the sequences were mapped onto our genome, accounting for 94.41 % of all BAC double-ended sequences. The reason that 1,065 (5.59 %) of the sequences did not map to the genome is most likely due to individual differences. The results also showed that 1,184 out of 1,300 contigs from the physical map could be mapped onto our genome (Table S6).

Gene family expansion and contraction

To understand the relationships of *C. mollissima* gene families to those of other plants, we performed a systematic comparison of genes among different species. The protein-coding genes of nine genomes, namely, *O. sativa* [31], *Malus domestica* [32], *P. trichocarpa* [33], *P. persica* [34], *C. mollissima*, *Q. robur* [35], *Fagus sylvatica* [36], *Juglans regia* [37] and *V. vinifera* [38], were used for the comparison. Gene loss and gain are among the primary reasons for functional changes. To gain greater insights into the evolutionary dynamics of the genes, we determined the expansion and contraction of the orthologous gene clusters in these eight species with CAFE software [39]. In the Chinese chestnut genome, a total of 17,422 gene families were identified, while 27,502 families of homologous genes were detected across the nine species. Of all the gene families (17,422), 209 were significantly expanded and 89 were contracted ($P < 0.05$) in *C. mollissima* (Fig. S3). The Venn diagram in Fig. 2a shows that 9,336 gene families were shared by the four species *C. mollissima*, *Q. robur*, *J. regia* and *F. sylvatica.* In addition, both specific and common gene families were detected in these four species. A total of 11,952 genes and 8,884 gene families were found to be specific to Chinese chestnut (Table S7).

Phylogenetic analysis

To examine the evolutionary relationships of Chinese chestnut with other plants, we applied RAxML software (version 8.0.0; substitution model PROTGAMMAJTT, bootstrap value 100) [40] to perform a maximum likelihood genome-wide phylogenetic analysis of 540 single-copy genes from the nine plant genomes (Fig. 2b). The results support the hypothesis that Chinese chestnut and oak are sister groups. Based on the phylogeny and fossil record [5], we estimated the divergence time. The phylogenetic tree indicates that the orders Fagales and Rosales have a close genetic relationship, with a divergence time of 90.75 million years ago (Mya). The estimated

divergence time of *C. mollissima* and *Q. robur* in the Fagales clade is approximately 13.62 Mya, while that of Chinese chestnut and *J. regia* is 62.7 Mya.

Long terminal repeat (LTR) insertion

In the final assembly, approximately 390 Mb of repetitive sequence was found, accounting for 49.69 % of the genome. LTR elements, accounting for 19.92 % of the genome of *C. mollissima*, are the most abundant transposable elements (Table S4). To estimate the insertion times of the LTR elements, we identified complete LTRs using a combination of *de novo* searches and manual inspection with LTR_Finder [41]. Finally, 5,470 complete LTRs were identified. We calculated the nucleotide distance for each of the 5,470 complete LTR elements using the molecular paleontology approach described by SanMiguel et al. [42] (Fig. 3 and Table S8). The average nucleotide distance of the LTR sequence pairs was 0.007681. When a substitution rate of 2.20×10^{-9} mutations per synonymous site per year was used, the insertion time distribution of the detected LTR elements indicated that the largest number of insertions occurred between 0 and 1.74 Mya [43].

Tandemly arrayed genes

Tandemly arrayed genes (TAGs) are gene clusters created by tandem duplication, and TAGs represent a large proportion of the genes in a genome [44]. To identify TAGs, we applied OrthoMCL with the default parameters to cluster genes into putative gene families. Subsequently, 1,122 TAGs were found by an in-house script; the duplicated genes were separated by less than 10 spacers (Fig. S4). These gene clusters contain 4,198 tandemly duplicated genes, accounting for 11.5 % of the total number of genes in *C. mollissima*, suggesting that a relatively high abundance of TAGs is a major feature of this genome. The TAGs of *C. mollissima* were compared with those of related species: *F. sylvatica* and *Q. robur* in the Fagaceae and *J. regia*, *M. domestica*, *P. persica* and *P. trichocarpa*. The percentage of TAGs in the complete genome of *C.*

mollissima was markedly higher than those of *P. trichocarpa* (4.9 %) and *M. domestica* (4.2 %). The TAG percentage was also high in other Fagaceae species, such as *Q. robur* (19.7 %) and *F. sylvatica* (8.0 %). However, this trait was not shared with *J. regia*, another species closely related to *C. mollissima*, which has only 5.6 % TAGs. Furthermore, TAGs can also be highly abundant in non-Fagales species, such as *P. persica* (13.3 %) (Table S9). GO enrichment analysis of genes from the TAGs was performed using OmicShare Tools (https://omictools.com/). The results showed that these genes are enriched in the cell binding and catalytic activity pathways in the cellular component category (Fig. S5 and Table S10).

Conclusions

In this study, a high-quality annotated genome sequence of *C. mollissima* was obtained, similar to those of other Fagaceae species, and it was found to contain a relatively high proportion of tandemly repeated genes. The Chinese chestnut genome will serve as a reference genome and pave the way for future research involving comparative genomics, and studies on domestication, genetic improvement and breeding for disease resistance and nut quality in chestnuts.

Availability of supporting data

Additional files

Table S1: Comparison of assembly quality in two genomes of *C. mollissima*

Table S2: Statistics of clean data of *C.mollissima* for Illumina and PacBio sequencing

Table S3: Comparison of genome size and heterozygosity in three species of *C. mollissima*, *Q. robur* and *F. sylvatica*.

Table S4: Statistics of repeat elements for *C. mollissima* assembly using both RepeatModeler and RepeatMasker software

Table S5: Core gene estimation for *C. mollissima* assembly using BUSCO

Table S6: The alignment between the assembled genome and the physical map of *C. mollissima*

Table S7: Unique gene families of *C. mollissima* in four species

Table S8: Complete LTR elements in *C. mollissima*

Table S9: Numbers and proportions of TAGs in *C. mollissima* and other species

Table S10: Tandemly arrayed genes (TAGs) in *C. mollissima*

Figure S1: K-mer distribution of *C. mollissima*

Figure S2: GO term analysis for genes in *C. mollissima*

Figure S3: Analysis of the expanded and contracted gene families in *C. mollissima*.

Figure S4: Tandemly arrayed genes (TAGs) numbers in one cluster in *C. mollissima*

Figure S5: GO enrichment of genes from the TAGs in *C. mollissima*

Competing interests

The authors declare that they have no competing interests.

Authors' contributions

YX and LQ designed the project; YL, XN and GW collected samples and extracted the DNA samples; YX, QC, QZ, HL, ZZ and YS worked on sequencing and data analyzing; YX and YS wrote the manuscript; HH, KF, and TB revised the manuscript; QC and LQ read and approved the final version of the manuscript.

Acknowledgements

This work was supported by grants from the National Key Research & Development Program of China (2018YFD1000605); the National Natural Science Foundation of China (31870671; 31672135); the Project of Construction of Innovative Teams and Teacher Career Development for Universities and Colleges under Beijing Municipality (IDHT20180509); Supporting Plan for Cultivating High Level Teachers in Colleges and Universities in Beijing (CIT&TCD20180317).

References

1. Jaynes R. Chestnut. In: Moore, J. (Ed.) Advances in Fruit Breeding. Purdue University Press, West Lafayette, USA; 1975. pp 490-503.
2. Lang P, Dane F, Kubisiak TL, et al. Molecular evidence for an Asian origin and a unique westward migration of species in the genus *Castanea* via Europe to North America. Molecular Phylogenetics and Evolution 2007; **43** (1): 49-59. https://doi.org/10.1016/j.ympev.2006.07.022.
3. Martín MA, Herrera MA, and Martín LM. In situ conservation and landscape genetics in forest species. Journal of Natural Resources and Development 2012; **2** (3): 1-5. https://doi.org/10.5027/jnrd.v2i0.01.
4. Zhang YH, Liu L, Liang WJ, Zhang YM. China fruit monograph: Chinese chestnut and Chinese hazelnut volume. China Forestry Press, Beijing, China; 2005.
5. Hao FW, Zhang FR. Textual research on the cultivition histroy of *Castanea mollissima* in China. Ancient and Modern Agriculture 2014; **3**: 40-48.
6. FAO. Food and Agriculture Organization of the United Nations. FAOSTAT Statistics Database 2017. Available from: http://www.fao.org/faostat/en/#home. Accessed 01 Apr 2019.
7. Jacobs DF, Dalgleish HJ, Nelson CD. A conceptual framework for restoration of threatened plants: the effective model of American chestnut (*Castanea dentata*) reintroduction. New Phytologist 2013; **197** (2): 378-393. https://doi:10.1111/nph.12020.
8. Hebard FV. The backcross breeding program of the American chestnut foundation. Journal of the American Chestnut Foundation 2006; **19**: 55-77.
9. Kremer A, Abbott AG, Carlson JE, et al. Genomics of Fagaceae. Tree Genetics & Genomes 2012; **8** (3): 583-610. https://doi.org/10.1007/s11295-012-0498-3.
10. Popkin G. Can a transgenic chestnut restore a forest icon?. Science 2018; **361** (6405): 830-831. https://doi:10.1126/science.361.6405.830.

11. Roane MK, Griffin GJ, Elkins JR. Chestnut blight, other *Endothia* diseases, and the genus *Endothia*. American Phytopathol Society Monograph Series, St. Paul, Minnesota, USA; 1986.
12. Kubisiak TL, Nelson CD, Staton ME, et al. A transcriptome-based genetic map of Chinese chestnut (*Castanea mollissima*) and identification of regions of segmental homology with peach (*Prunus persica*). Tree Genetics & Genomes 2013; **9** (2): 557-571. https://doi.org/10.1007/s11295-012-0579-3.
13. Staton M, Zhebentyayeva T, Olukolu B, et al. Substantial genome synteny preservation among woody angiosperm species: comparative genomics of Chinese chestnut (*Castanea mollissima*) and plant reference genomes. BMC Genomics 2015; **16** (1) 744. https://doi.org/10.1186/s12864-015-1942-1.
14. Santos C, Nelson CD, Zhebentyayeva T, et al. First interspecific genetic linkage map for *Castanea sativa* × *Castanea crenata* revealed QTLs for resistance to *Phytophthora cinnamomi*. Plos One 2017; **12** (9): e0184381. https://doi.org/10.1371/journal.pone.0184381.
15. Staton M, Addo-Quaye C, Cannon N, et al. The Chinese chestnut genome: a reference for species restoration. bioRxiv, Cold Spring Harbor Labs Journals; 2019.
16. Barakat A, Staton M, Cheng CH, et al. Chestnut resistance to the blight disease: insights from transcriptome analysis. BMC Plant Biology 2012; **12** (1): 38. https://doi.org/10.1186/1471-2229-12-38.
17. Ji FY, Wei W, Liu Y, et al. Construction of a SNP-based high-density genetic map using genotyping by sequencing (GBS) and QTL analysis of nut traits in Chinese chestnut (*Castanea mollissima* Blume). Frontiers in Plant Science 2018; **9**: 816. https://doi.org/10.3389/fpls.2018.00816.
18. Zhang L, Lin Q, Feng YZ, et al. Transcriptomic identification and expression of starch and sucrose metabolism genes in the seeds of Chinese chestnut (*Castanea mollissima*). Journal of Agricultural and Food Chemistry 2015; **63** (3): 929-942. https://doi.org/10.1021/jf505247d.

19. Li M, Tian S, Jin L, et al. Genomic analyses identify distinct patterns of selection in domesticated pigs and Tibetan wild boars. Nature Genetics 2013; **45** (12): 1431-1438. https://doi.org/10.1038/ng.2811.

20. Zhang T, Hu Y, Jiang W, et al. Sequencing of allotetraploid cotton (*Gossypium hirsutum* L. acc. TM-1) provides a resource for fiber improvement. Nature Biotechnology 2015; **33** (5): 531-537. https://doi.org/10.1038/nbt.3207.

21. Marçais G, Kingsford C. A fast, lock-free approach for efficient parallel counting of occurrences of *k*-mers. Bioinformatics 2011; **27** (6): 764-770. https://doi.org/10.1093/bioinformatics/btr011.

22. Ramos AM, Usié A, Barbosa P, et al. Data Descriptor: The draft genome sequence of cork oak. Scientific Data 2018; **5**: 180069. https://doi.org/10.1038/sdata.2018.69.

23. Tarailo-Graovac M, Chen NS. Using RepeatMasker to identify repetitive elements in genomic sequences. Current Protocols in Bioinformatics 2009; **25**: 4.10.1-4.10.14. https://doi.org/10.1002/0471250953.bi0410s25.

24. Slater GSC and Birney E. Automated generation of heuristics for biological sequence comparison. BMC Bioinformatics 2005; **6**: 31. https://doi:10.1186/1471-2105-6-31.

25. Jones P, Binns D, Chang HY, et al. InterProScan 5: genome-scale protein function classification. Bioinformatics 2014; **30** (9): 1236-1240. https://doi.org/10.1093/bioinformatics/btu031.

26. Wheeler TJ, Eddy SR. nhmmer: DNA homology search with profile HMMs. Bioinformatics 2013; **29** (19): 2487-2489. https://doi.org/10.1093/bioinformatics/btt403.

27. Li H, Durbin R. Fast and accurate long-read alignment with Burrows-Wheeler transform. Bioinformatics 2010; **26** (5): 589-595. https://doi.org/10.1093/bioinformatics/btp698.

28. Kim D, Langmead B, Salzberg SL. HISAT: a fast spliced aligner with low memory requirements. Nature Methods 2015; **12** (4): 357-360. https://doi.org/10.1038/nmeth.3317.

29. Waterhouse RM, Seppey M, Simão FA, et al. BUSCO applications from quality assessments to gene prediction and phylogenomics. Molecular Biology and Evolution 2018; **35** (3): 543-548. https://doi.org/10.1093/molbev/msx319.
30. Fang GC, Blackmon BP, Staton ME, et al. A physical map of the Chinese chestnut (*Castanea mollissima*) genome and its integration with the genetic map. Tree Genetics & Genomes 2013; **9** (2): 525-537. https://doi.org/10.1007/s11295-012-0576-6.
31. International Rice Genome Sequencing Project. The map-based sequence of the rice genome. Nature 2005; **436** (7052): 793-800. https://doi.org/10.1038/nature03895.
32. Velasco R, Zharkikh A, Affourtit J, et al. The genome of the domesticated apple (*Malus* × *domestica* Borkh.). Nature Genetics 2010; **42** (10): 833-839. https://doi.org/10.1038/ng.654.
33. Tuskan GA, Difazio S, Jansson S, et al. The genome of black cottonwood, *Populus trichocarpa* (Torr. & Gray). Science 2006; **313** (5793): 1596-1604. https://doi.org/10.1126/science.1128691.
34. Verde I, Abbott AG, Scalabrin S, et al. The high-quality draft genome of peach (*Prunus persica*) identifies unique patterns of genetic diversity, domestication and genome evolution. Nature Genetics 2013; **45** (5): 487-494. https://doi.org/10.1038/ng.2586.
35. Plomion C, Aury JM, Amselem J, et al. Oak genome reveals facets of long lifespan. Nature Plants 2018; **4** (7): 440-452. https://doi.org/10.1038/s41477-018-0172-3.
36. Mishra B, Gupta DK, Pfenninger M, et al. A reference genome of the European beech (*Fagus sylvatica* L.). GigaScience 2018; **7** (6): 1-8. https://doi.org/10.1093/gigascience/giy063.
37. Martínez-García PJ, Crepeau MW, Puiu D, et al. The walnut (*Juglans regia*) genome sequence reveals diversity in genes coding for the biosynthesis of non-structural polyphenols. The Plant Journal 2016; **87** (5): 507-532. https://doi.org/10.1111/tpj.13207.

38. The French-Italian Public Consortium for Grapevine Genome Characterization. The grapevine genome sequence suggests ancestral hexaploidization in major angiosperm phyla. Nature 2007; **449** (7161): 463-467. https://doi.org/10.1038/nature06148.

39. De Bie T, Cristianini N, Demuth J, et al. CAFE: a computational tool for the study of gene family evolution. Bioinformatics 2006; **22** (10): 1269-1271. https://doi.org/10.1093/bioinformatics/btl097.

40. Stamatakis A. RAxML version 8: a tool for phylogenetic analysis and post-analysis of large phylogenies. Bioinformatics 2014; **30** (9): 1312-1313. https://doi.org/10.1093/bioinformatics/btu033.

41. Xu Z, Wang H. LTR_FINDER: an efficient tool for the prediction of full-length LTR retrotransposons. Nucleic Acids Research 2007; **35** (Web Server issue): W265-W268. https://doi.org/10.1093/nar/gkm286.

42. SanMiguel P, Gaut BS, Tikhonov A, et al. The paleontology of intergene retrotransposons of maize. Nature Genetics 1998; **20** (1): 43-45. https://doi.org/10.1038/1695.

43. Björn N, Nathaniel RS, Anna W, et al. The Norway spruce genome sequence and conifer genome evolution. Nature 2013, **497** (7451): 579-584. https://doi.org/10.1038/nature12211.

44. Pan D, Zhang LQ. Tandemly arrayed genes in vertebrate genomes. Comparative and Functional Genomics 2008; **2008**: 1-11. https://doi.org/10.1155/2008/545269.

Table：

Table 1 Summary of *C. mollissima* genome assembly and gene model

**Genome assembly statistics**	
Total length	785,529,252 bp
Number of Contigs	2,707
Largest Contig Length	6,584,328 bp
N50 length (Contigs)	944,461 bp
N90 length (Contigs)	133,678 bp
Counts of N50 (Contigs)	235
Counts of N90 (Contigs)	1,024
**Gene model statistics**	
Gene number	36,479
Gene density (per 100 kb)	4.64
Gene average length	1,139.63 bp
Exon number per Gene	4.41
Exon average length	258.15 bp
Intron average length	1,156.91 bp
Genome GC percent	36.07 %
Exon GC percent	43.36 %

Figure:

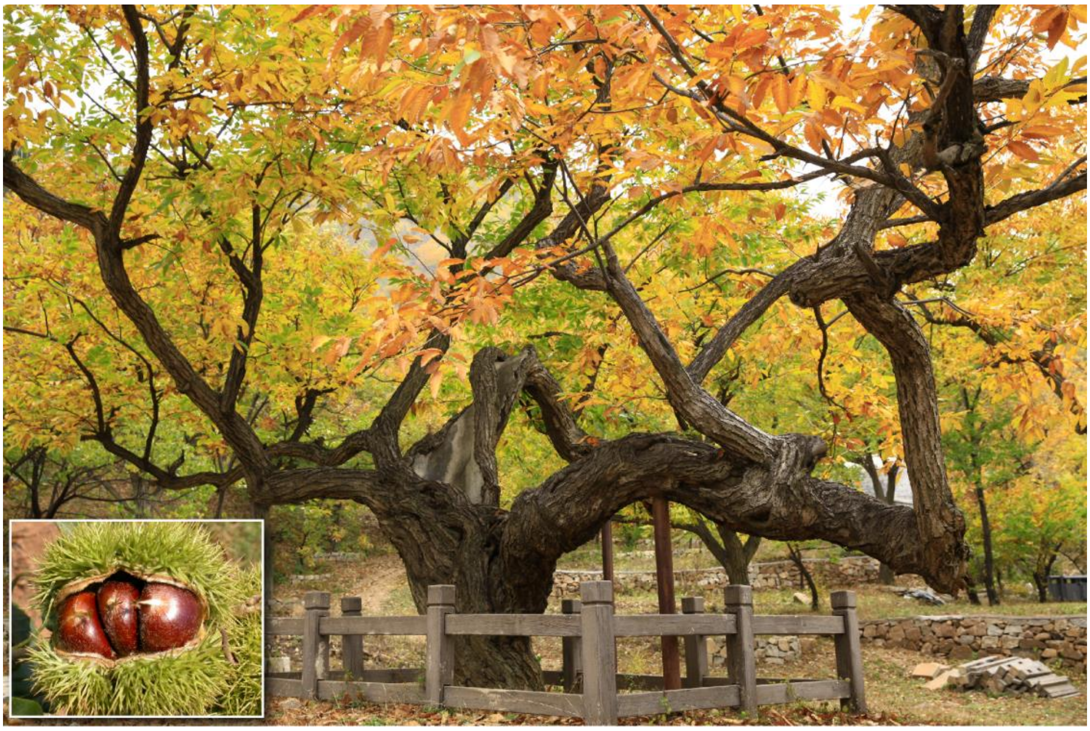

Figure 1 Example of Chinese chestnut tree (*C. mollissima*). Natural habitat of *C. mollissima* (image from the Water Great Wall, Beijing, China) and the nut of *C. mollissima* (image from Ling Qin) are showed.

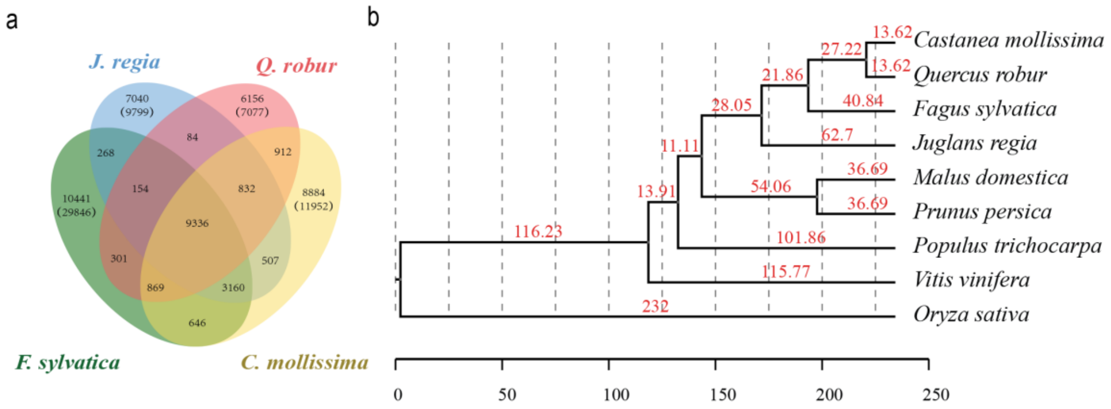

Figure 2 Phylogenetic relationships between Chinese chestnut and other species. A maximum-likelihood tree was obtained with 540 single copy orthologous genes. a) The shared and unique gene families in four closely related species are shown in the Venn diagram. Each number represents a number of gene families, and the number in brackets is a number of genes. b) The divergence times were estimated and are displayed on the phylogenetic tree.

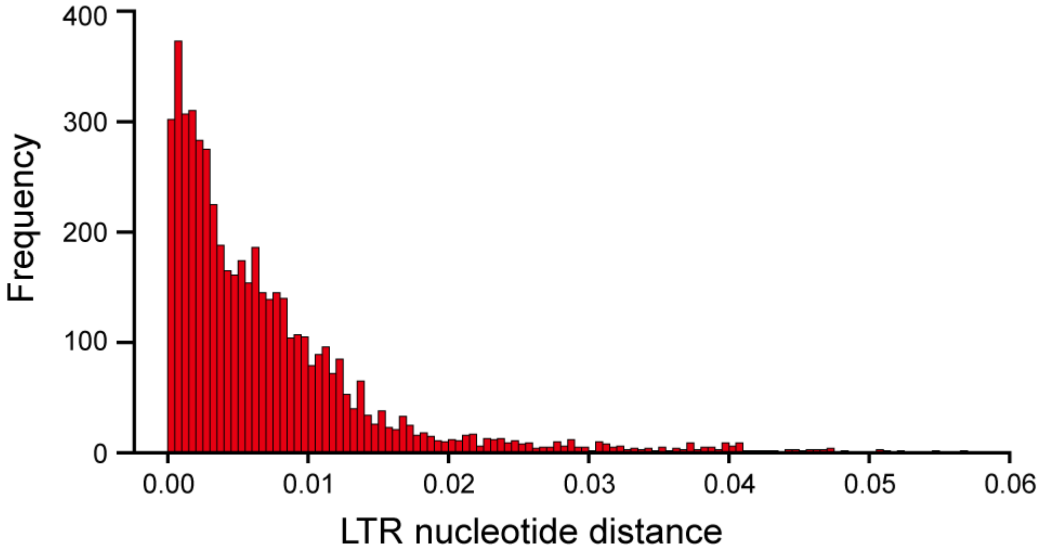

Figure 3 Nucleotide distance distribution of annotated LTR elements in *C. mollissima*.

Click here to access/download
Supplementary Material
Tables (S1-S5, S9).doc

Supplementary Tables S6

Click here to access/download
Supplementary Material
Table S6.xls

Supplementary Tables S7

Click here to access/download
Supplementary Material
Table S7.xls

Supplementary Tables S8

Click here to access/download
Supplementary Material
Table S8.xls

Supplementary Tables S10

Click here to access/download
Supplementary Material
Table S10.xls

Supplementary Figures(S1-S5)

Click here to access/download
Supplementary Material
Figure (S1-S5).doc

Click here to access/download
Supplementary Material
Revised Protocols.doc

GIGA-D-18-00448R1
Hybrid de novo genome assembly of Chinese chestnut (*Castanea mollissima*)
Yu Xing; Yang Liu; Qing Zhang; Xinghua Nie; Yamin Sun; Zhiyong Zhang; Huchen Li; Kefeng Fang; Guangpeng Wang; Hongwen Huang; Ton Bisseling; Qingqin Cao; LING QIN
GigaScience

Dear Dr Qin,
Your manuscript "Hybrid de novo genome assembly of Chinese chestnut (*Castanea mollissima*)" (GIGA-D-18-00448R1) has been re-reviewed by our reviewers. Although it is of interest, we are still unable to consider it for publication without some additional work. The reviewers have raised a number of points which we believe would further improve the manuscript and would then allow a revised version to be published in GigaScience. In particular validation/comparisons with other related genomes is still required. And we need all the gff files, custom scripts and other intermediate and processed resources.

Their reports, together with any other comments, are below. Please also take a moment to check our website at https://www.editorialmanager.com/giga/ for any additional comments that were saved as attachments.

If you are able to fully address these points, we would encourage you to submit a revised manuscript to GigaScience. Once you have made the necessary corrections, please submit online at:

https://www.editorialmanager.com/giga/

If you have forgotten your username or password please use the "Send Login Details" link to get your login information. For security reasons, your password will be reset.

Please include a point-by-point within the 'Response to Reviewers' box in the submission system. Please ensure you describe additional experiments that were carried out and include a detailed rebuttal of any criticisms or requested revisions that you disagreed with. Please also ensure that your revised manuscript conforms to the journal style, which can be found in the Instructions for Authors on the journal homepage.

The due date for submitting the revised version of your article is 28 Jul 2019.

I look forward to receiving your revised manuscript soon.

Best wishes,
Hongling Zhou
GigaScience
www.gigasciencejournal.com

Dear editor,

After reading carefully the reviewers' comments, we tried our best to answer all the questions point-by-point. As you know, the Chinese chestnut genome has been preprinted on bioRxiv. We compared the assembly quality of our genome with theirs, as shown in Table S1, and all the data support our genome assembly quality is better than them. We also added those comparison results in the part of "background information". Because only Assembly V1.1 of Staton et al. 2019 is released on the website of hardwood genomics (https://www.hardwoodgenomics.org/), so we also downloaded and compared the genome assembly quality with the Assembly V1.1 of Staton et al. 2019, individually. The results were listed in the Table S1, Table S11 and Table S12 below. As your requirement, we have also uploaded all the gff files, custom scripts and other intermediate and processed resources on the ftp.

Table S1. Comparison of assembly quality in two genomes of *C. mollissima*

**Species**	***C.mollissima* (in this study)**	***C. mollissima*（Staton et al, 2019）**			
		**Assembly 1.1**	**Assembly 2.0**	**Assembly 3.2**	**Assembly 4.0**
Total length of contigs sequences	785.53 Mb	724.4 Mb	760 Mb	783.4 Mb	783.4 Mb
Maximum length of contigs	6.6 Mb	2.8 Kb	3.58 Kb	1.1 Mb	1.1 Mb
Counts of contigs sequences	2,707	71,043	60,546	12,684	12,684
Counts of scaffolds sequences	2,707	41,260	14,358	/	/
Contigs length range	1 Kb to 6.6 Mb	/	2 Kb - 3.58 Kb	2Kb - 1.1 Mb	2Kb - 1.1 Mb
Scaffolds length range	1 Kb to 6.6 Mb	2 Kb - 429 Kb	2K - 5.5 M	/	/
Counts of N50	235	5,021	/	/	/
Scaffold N50	944,461 bp	39,561 bp	2.75 Mb	/	/

Table S11 Comparison based on scaffold in two genomes of *C. mollissima*

**Species**	***C.mollissima*** **(Staton et al. V1.1)**	***C.mollissima*** **(in this study)**
Counts of scaffold sequences	41,260	2,707
Length of scaffold sequences	724,001,627 bp	785,529,252 bp
Largest scaffold length	429,344 bp	6,584,328 bp
Scaffold N50	**39,561 bp**	**944,461 bp**
Counts of N50	5,021	235
Scaffold N90	6,866 bp	133,678 bp
Counts of N90	21,603	1,024
GC content (%)	34.47%	35.21%

Table S12 Comparison based on contigs in two genomes of *C. mollissima*

**Species**	***C. mollissima*** **(Staton et al. V1.1)**	***C. mollissima*** **(in this study)**
Counts of contigs	71,043	2,707
Maximum length of contigs	280,842 bp	6,584,328 bp
contig N50	**22,008 bp**	**944,461 bp**
Counts of contig N50	8,663	235
contig N90	4,049 bp	133,678 bp
Counts of contig N90	38,234	1,024

Reviewer reports:

Reviewer #1: The authors present an improved manuscript "Hybrid de novo genome assembly of Chinese chestnut (*Castanea mollissima*) " and my major compulsory revisions were taken into account. The authors compare the heterozygosity rate of *C. mollissima* with other Fagaceae species, and I think they should add these results to a supplementary table and how they were obtained in the main manuscript (section Genome size and heterozygosity estimation).

Answer: The size of the *C. mollissima* genome was estimated to be approximately 772 Mb, and the heterozygosity level of *C. mollissima* was approximately 0.87 % (Table S2). Comparing this estimate with those for beech and oak, we found that our result was more similar to European beech. The heterozygosity rate of oak is from Ramos AM et al., 2018 [22]. In the genome literature of European beech [36], the heterozygosity rate is not mentioned clearly. In order to compare the heterozygosity rate in three species, we download the PRJEB24056 (ERX2326485 and EXR2326486) from NBCI database and estimated the heterozygosity rate is approximately 0.63% based on k-mers method.

Reviewer #2: The manuscript has been significantly improved over the previous version, even though I feel that literature on the subject is still not fully covered. For example, Staton et al. 2015 https://doi.org/10.1186/s12864-015-1942-1 is not mentioned, and no attempts seem to have been made to compare the results of this study to those generated by other authors on the same tree species over the past 5 years.

Answer: Thank you for the suggestion and we have addressed the relevant published literatures of chestnut over the past five years and also added some comparison with our results in the "Background information" and "Results" sections.

line 86 - The sonication device used should be mentioned, as well as the parameters used for fragmentation.

Answer: The DNA was sheared by Covaris S2 system (Covaris, USA) for short insert paired-end (PE) library construction. The shearing conditions were as the following: the number of cycles is 2 and shearing time is 40 seconds per cycle.

lines 109-115 - I am still not fully convinced by this strategy. A data cleanup before the assembly might have been better. In any case, it should be mentioned, how sequence data of potentially contaminant organisms were searched for and the results of the searches should be presented.

Answer: Thank you for the reviewer's suggestion. Actually, we did data cleanup before the assembly. The raw data have been filtered and trimmed. Illumina data quality control settings are as follows: SLIDINGWINDOW: 4: 15 MINLEN: 50 using software

as Trimmomatic. Pacbio data quality control standard is RQ>0.75 and the minimum subreads length is 500 bp using software SMRT Link 6.0. The Table S2 is added to show the comparison between Polymerase reads and Subreads from PacBio sequencing. The detailed method for data quality control is added in the protocol.

To avoid potential contaminant, we carried out the contaminant check on the raw data of PacBio sequencing. The detailed method is as follows: 1000 subreads sequences are randomly selected to search against the NT database using BLAST (threshold settings -F F -e 1e-5). If a subread shows the highest alignment with sequences from non-plant species, it is identified as contaminant. In our results, 216 in 1000 subreads are blasted against the NT database and all the 216 subreads are belong to Viridiplantae. No contaminant read is detected in randomly selected 1000 subreads. We also blasted all the 36,479 protein-coding genes in our genome against the NT database. 35,544 genes are blasted and 35,353 genes are classified into Viridiplantae. The results have been uploaded as intermediate files to the ftp.

Table S2. Statistics of clean data of *C.mollissima* for Illumina and PacBio sequencing.

**Clean Reads**	**Illumina Sequencing**		**PacBio Sequencing**	
	**Raw reads**	**Clean reads**	**Polymerase reads**	**Subreads (RQ>0.75 length>500)**
Size of library	500 bp	500 bp	20 kb	20 kb
Number of reads	118,123,542*2	113,618,248*2	4,961,145	9,581,541
Average length of reads	150 bp	147 bp	13,971 bp	7,170 bp
Total bases	35,437,062,600 bp	33,580,328,092 bp	69,312,850,332 bp	68,707,578,456 bp
Sequencing depth	~45.1 X	~42.7 X	~639 X	~87 X

lines 136-138 - Details regarding how the gene boundaries from the Evidence Modeler output were verified are missing.

Answer: The In-house script was used to scan and count the initial codon, termination codon, exon and intron boundaries of each gene. The results and scripts were provided in the intermediate files. We have uploaded the intermediate files to the ftp.

line 210 - The script needs to be made accessable in order to make the results reproducible.

Answer: All the scripts were provided in the intermediate files and uploaded to the ftp.

 Click here to access/download;Personal Cover;Cover letter.doc

Dear editor,

Thank you very much for your kindly reminder letter and for the reviewers' comments concerning our manuscript entitled "Hybrid *de novo* genome assembly of Chinese chestnut (*Castanea mollissima*)" (GIGA-D-18-00448).

The comments were all valuable and very helpful for us to improve our manuscript. We have studied the comments carefully and made corrections accordingly. Attached please find the revised version, which we would like to submit for your kind consideration.

For your convenience, we have already uploaded all the gff files, custom scripts and other intermediate and processed resources to the ftp.

After reading carefully the reviewers' comments, we tried our best to answer all the questions point-by-point. As you know, the Chinese chestnut genome has been preprinted on bioRxiv. We compared the assembly quality of our genome with theirs, as shown in Table S1, and all the data support our genome assembly quality is better than them. We also added those comparison in the part of "background information". We hope this situation of competing interests will speed up the publication of our manuscript in a timely manner.

We would like to express our appreciation to you and the reviewers for your comments on our paper. We look forward to hearing from you.

Thank you and best regards.

Yours sincerely,

Ling Qin

Ling Qin, Prof.
Beijing University of Agriculture, Beijing, 102206, China
E-mail: qinlingbac@126.com
