# Supplementary material for: Hybrid de novo genome assembly of Chinese chestnut (Castanea mollissima)
Source: Gigascience. 2019 Sep 12;8(9):giz112. doi: 10.1093/gigascience/giz112 (PMC6741814; doi:10.1093/gigascience/giz112)
Supplement: giz112_Response_to_Reviewer_Comments_Original_Submission [file giz112_response_to_reviewer_comments_original_submission.pdf]

Author's Response To Reviewer Comments

Close

We have also uploaded our response to reviewers' comments in the attach files named 'Reviewers_comments' because there are some figures and tables in the file of response to reviewers' comments. Please see the detailed response in the attached files. We also listed the response to the two reviewers' comments without figures and tables here.

Reviewer 1:

The article describes the sequencing, assembly and annotation of the Chinese chestnut tree (Castanea mollisima) as well as some analysis based on gene families. My main concern about the manuscript is related to the availability of the sequencing data. Indeed the genomic and transcriptomic data are not available through SRA portal. On the other hand, the comparative analyses are not of equal quality. For example, the functional enrichment of specific gene families has not been inspected when compared to the TAGs. In addition, a few points need to be addressed.

A: For the reviewers' convenience, we have already uploaded our raw data to the NCBI SRA database; the BioProject # is PRJNA527178 (SRA # of transcriptome: SRR8731962, SRA # of PacBio reads: SRR8755082-SRR8755097, and SRA # of Illumina reads: SRR8731963), and we have also confirmed that all the data are available now.

Sampling and Sequencing:
- Line 31: Genome DNA instead of Genomic DNA.

A: Yes. We have revised it in the line 82 on page 4 of the revised manuscript.

- Line 39: approximately 54 Gb, where Table S1 reports 39 Gb.

A: The data in Table S1 are correct, and we have double-checked and verified the data in the revised manuscript and tables and corrected the typos in the revised manuscript.

- Line 49: In total 80 Gb, where Table S1 reports 69 Gb.

A: The data in Table S1 are correct, and we have double-checked and verified the data in the revised manuscript and tables and corrected the typos in the revised manuscript.

Genome size and heterozygosity estimation:
- The authors report a heterozygosity rate of approximately 0.17%, I think it is of interest to compare this rate with other trees, like beech or oak.

A: The genome size and heterozygosity rate were evaluated before we started the genome sequencing project, and the heterozygosity rate that we obtained was 0.17%. Based on our common sense and a comparison with data from other trees, we thought the results were unreasonable. Therefore, we double-checked the raw data and found some bacterial contamination in the Illumina raw data, which caused low coverage depth and resulted in inaccurate genome size and heterozygosity rate estimations. Therefore, we resequenced the samples without bacterial contamination using the Illumina HiSeq 2500 sequencer and then used the new data to perform the same analyses (genome size and heterozygosity estimation, genome assembly and error correction). However, the initial genome size and heterozygosity estimates (0.17%) from the previous report using the contaminated data were mistakenly used in the manuscript submitted for review. We have corrected these estimates in the revised manuscript, and the correct heterozygosity rate should be 0.87%. Comparing this estimate with those for beech (heterozygosity rate 0.19%) and oak (heterozygosity rate ~1%), we found that our result was more similar to oak (Figure S1).

Genome assembly and annotation:

- The authors describe the polishing step made using Quiver, but it is unclear how the several (how many?) rounds of iterative error correction, with short reads, were performed.

A：We performed six rounds of interactive error correction with the Illumina data and have added this information to line 111-113 on page 5 of the revised manuscript.

- The annotation process is not sufficiently described. The authors should describe which databases and which tools were used, and they should describe the transcriptomic dataset.

A: In the revised manuscript, we describe the annotation process in detail. In addition, we describe a transcriptomic dataset, which has been uploaded to the SRA database. We validated the exon/intron boundaries using transcripts and the GU-AG rule. We identified 36,479 protein-coding genes in the C. mollissima genome. In detail, 47,666 genes were predicted through the de novo method, 26,647 genes were annotated based on RNA transcripts or raw RNA reads, and 14,695 genes were supported by homology. We also listed the gene numbers that could be annotated in the different databases. For your convenience, we listed the gene numbers that we used and integrated to predict our protein-coding genes in the following table.

Quality assessment:
- line 28: genetic regions instead of genic regions.

A: Yes. We have revised it in the line 152 on page 7 of the revised manuscript.

Gene family expansion and contraction
- A total of 956 and 471 gene families were found to be specific to Chinese chestnut and oak. However, on Figure 2a that refers to gene families, the numbers are different. The authors should clarify the text and/or the legend of Figure 2.

A: We have corrected these numbers in the revised manuscript. In addition, in accordance with the reviewers' suggestions, we adjusted the text and the legend of Figure 2a to increase clarity, and we recreated the Venn diagram using C. mollissima, Q. robur, J. regia and F. sylvatica. Please see the "Gene family expansion and contraction" section (lines 163-178, pages 7-8) of the revised manuscript (Figure 2).

Tandemly arrayed genes
- The authors claim that "TAGs are gene clusters created by tandem duplication, and TAGs represent a large proportion of the genes in a genome". Authors should add citations to support this sentence.

A: We have cited a reference to support this sentence.
Pan D, Zhang LQ. Tandemly arrayed genes in vertebrate genomes. Comparative and functional genomics 2008; 2008: 1-11. https://doi.org/10.1155/2008/545269.

- The authors suggest that an abundance of TAGs is a major feature if the genome, but they should compare to other species, such as the oak genome which contains a high proportion of TAGs (as shown in Plomion et al, 2018).

A: According to this suggestion, we compared the number and proportion of TAGs in different species and added them to the revised manuscript as supplementary Table S7. Please see the "Tandemly arrayed genes" section (lines 204-222, page 9) of the revised manuscript (Table S7).

Reviewer2: First of all, I congratulate the authors on there effort and the valuable results obtained. The genome seems mostly well assembled, but there are some issues as outlined below that should be addressed.

The English is of some concern. There are only few grammar and spelling mistakes, but there are many sentences that are phrased in an odd manner, sometimes there is also highly unusual word usage. The authors are thus strongly encouraged to have their manuscript proofread by a native English speaker.

A: The revised manuscript has been polished by a senior editor at American Journal Experts (https://secure.aje.com, contract #6G58F9VV) and proofread by Professor Rene Geurts, from Department of Molecular Biology, Wageningen University and Professor Jocelyn Rose, from School of Integrative Plant Science, Cornell University.

Title. The title should mention that a hybrid approach was used.

A: Yes. We have revised the title as follows: “Hybrid de novo genome assembly of Chinese chestnut (Castanea mollissima)”.

Page 5
Line 24. Castanea sativa has dominant role as forest tree only in few areas of Europe (e.g. some parts of northern Italy and southern France).
Lines 42-44. A citation should be given for this claim.
Lines 51-57. A citation should be given for this claim.

A: Yes. We have revised these passages and cited the references in the manuscript in lines 50-51, 55-58, and 60-64 on page 3 of the revised manuscript.

Page 6
Line 9. The available resources should be cited here, e.g. the genome published previously.

A: Yes. We have cited the references in the manuscript in lines 69-72 on page 4 of the revised manuscript.

Line 28. The sequenced indivual(s) should be described in more detail and the sampling date should be given.

A: Yes. We have described the sampling information in more detail in lines 77-83 on page 4 of the revised manuscript.

Genome assembly. As SMARTdenovo does not have a correction step, all data should have been corrected before assembly. At 100x, it might have been useful doing self-correction rather than Illumina-based filtering (first).

A: We assembled the genome using three software packages. Interestingly, the results indicated that the genome assembly performed with SMARTdenovo displayed higher continuity than those from the other two software packages. To achieve a high-accuracy genome assembly, the assembled sequence was then polished using Quiver (SMRT Analysis v2.3.0) with the default parameters, and six rounds of iterative error correction were performed using the Illumina clean reads.

Page 7
Lines 46-49. More detail needs to be provided here. From the information given, it is unclear how gene predictions were actually done.
A: Yes. We have added a detailed description to the “Genome assembly and annotation” section (lines 107-143, pages 5-6) of the revised manuscript.
Line 51. The output from the evidence modeler could be checked against transcripts to vailidate exon/intron boundaries.

A: We validated the exon/intron boundaries using transcripts and the GU-AG rule. We identified 36,479 protein-coding genes in the C. mollissima genome. In detail, 47,666 genes were predicted through the de novo method, 26,647 genes were annotated by RNA transcripts or raw RNA reads, and 14,695 genes were supported by homology. We also listed the numbers of genes that could be annotated with the different databases. For your convenience, we listed the gene numbers that we used and integrated to predict the protein-coding genes in the following table.

Page 8
Line 28. Most?

A: We revised the manuscript to improve clarity in line 152 on page 7.

Page 9
Gene family expansion and contraction/phylogenetic analysis. For both of these analyses, the genome of Fagus sylvatica should be included as well as an important reference point in Fagaceae. In the Venn diagram, it could replace Malus domestica, which is largely unrelated to chestnut and oak.

A: Yes. We have corrected this issue in the revised manuscript (lines 163-178, pages 7-8). We recreated the Venn diagram using C. mollissima, Q. robur, J. regia and F. sylvatica. In addition, in accordance with the reviewers' suggestions, we adjusted the text and the legend of Figure 2a to improve clarity (Figure 2).

Line 48. RAxML is a program not a method. It should be mentioned, which version and which substitution model were used, as well as the amount of bootstrap replicated done.

A: Yes. We have revised this text in lines 181-182 on page 8 of the revised manuscript.

Line 57. It should be mentioned where these divergence times are coming from. the authors could also do their own estimations using a dating software.

A: We estimated these divergence times using r8s. We selected three time scales from the time tree (http://www.timetree.org/). We also revised the manuscript to improve clarity.

Page 10
Tandemly arranged genes. This should be specifically compared to Quercus, Fagus and a species outside Fagaceae, such as walnut and poplar, to investigate, how special this trait is.

A: The tandemly arranged genes of C. mollissima were compared with those of closely related species in the genomes of F. sylvatica and Q. robur in the Fagaceae and the additional genomes of J. regia, M. domestica, P. persica and P. trichocarpa. The results are indicated in the "Tandemly arrayed genes" section (lines 204-222, page 9) of the manuscript and have also been added to the supplementary information as supplementary Table S7.

Close
