# Supplementary material for: Hybrid de novo genome assembly of Chinese chestnut (Castanea mollissima)
Source: Gigascience. 2019 Sep 12;8(9):giz112. doi: 10.1093/gigascience/giz112 (PMC6741814; doi:10.1093/gigascience/giz112)
Supplement: giz112_Response_to_Reviewer_Comments_Revision_1 [file giz112_response_to_reviewer_comments_revision_1.pdf]

Author's Response To Reviewer Comments

Close

Dear editor,
After reading carefully the reviewers' comments, we tried our best to answer all the questions point-by-point. As you know, the Chinese chestnut genome has been preprinted on bioRxiv. We compared the assembly quality of our genome with theirs, as shown in Table S1, and all the data support our genome assembly quality is better than them. We also added those comparison results in the part of "background information". Because only Assembly V1.1 of Staton et al. 2019 is released on the website of hardwood genomics (https://www.hardwoodgenomics.org/), so we also downloaded and compared the genome assembly quality with the Assembly V1.1 of Staton et al. 2019, individually. The results were listed in the Table S1, Table S11 and Table S12 below. As your requirement, we have also uploaded all the gff files, custom scripts and other intermediate and processed resources on the ftp.

Reviewer reports:

Reviewer #1: The authors present an improved manuscript "Hybrid de novo genome assembly of Chinese chestnut (Castanea mollissima) " and my major compulsory revisions were taken into account. The authors compare the heterozygosity rate of C. mollissima with other Fagaceae species, and I think they should add these results to a supplementary table and how they were obtained in the main manuscript (section Genome size and heterozygosity estimation).

Answer: The size of the C. mollissima genome was estimated to be approximately 772 Mb, and the heterozygosity level of C. mollissima was approximately 0.87 % (Table S2). Comparing this estimate with those for beech and oak, we found that our result was more similar to European beech. The heterozygosity rate of oak is from Ramos AM et al., 2018 [22]. In the genome literature of European beech [36], the heterozygosity rate is not mentioned clearly. In order to compare the heterozygosity rate in three species, we download the PRJEB24056 (ERX2326485 and EXR2326486) from NBCI database and estimated the heterozygosity rate is approximately 0.63% based on k-mers method.

Reviewer #2: The manuscript has been significantly improved over the previous version, even though I feel that literature on the subject is still not fully covered. For example, Staton et al. 2015 https://doi.org/10.1186/s12864-015-1942-1 is not mentioned, and no attempts seem to have been made to compare the results of this study to those generated by other authors on the same tree species over the past 5 years.

Answer: Thank you for the suggestion and we have addressed the relevant published literatures of chestnut over the past five years and also added some comparison with our results in the "Background information" and "Results" sections.

line 86 - The sonication device used should be mentioned, as well as the parameters used for fragmentation.

Answer: The DNA was sheared by Covaris S2 system (Covaris, USA) for short insert paired-end (PE) library construction. The shearing conditions were as the following: the number of cycles is 2 and shearing time is 40 seconds per cycle.

lines 109-115 - I am still not fully convinced by this strategy. A data cleanup before the assembly might have been better. In any case, it should be mentioned, how sequence data of potentially contaminant organisms were searched for and the results of the searches should be presented.

Answer: Thank you for the reviewer's suggestion. Actually, we did data cleanup before the assembly. The raw data have been filtered and trimmed. Illumina data quality control settings are as follows: SLIDINGWINDOW: 4: 15 MINLEN: 50 using software as Trimmomatic. Pacbio data quality control standard is RQ>0.75 and the minimum subreads length is 500 bp using software SMRT Link 6.0. The Table S2 is added to show the comparison between Polymerase reads and Subreads from PacBio

sequencing. The detailed method for data quality control is added in the protocol.
To avoid potential contaminant, we carried out the contaminant check on the raw data of PacBio sequencing. The detailed method is as follows: 1000 subreads sequences are randomly selected to search against the NT database using BLAST (threshold settings -F F -e 1e-5). If a subread shows the highest alignment with sequences from non-plant species, it is identified as contaminant. In our results, 216 in 1000 subreads are blasted against the NT database and all the 216 subreads are belong to Viridiplantae. No contaminant read is detected in randomly selected 1000 subreads. We also blasted all the 36,479 protein-coding genes in our genome against the NT database. 35,544 genes are blasted and 35,353 genes are classified into Viridiplantae. The results have been uploaded as intermediate files to the ftp.

lines 136-138 - Details regarding how the gene boundaries from the Evidence Modeler output were verified are missing.

Answer: The In-house script was used to scan and count the initial codon, termination codon, exon and intron boundaries of each gene. The results and scripts were provided in the intermediate files. We have uploaded the intermediate files to the ftp.

line 210 - The script needs to be made accessable in order to make the results reproducible.

Answer: All the scripts were provided in the intermediate files and uploaded to the ftp.

Close
