# Supplementary material for: Hybrid de novo genome assembly of Chinese chestnut (Castanea mollissima)
Source: Gigascience. 2019 Sep 12;8(9):giz112. doi: 10.1093/gigascience/giz112 (PMC6741814; doi:10.1093/gigascience/giz112)
Supplement: giz112_Response_to_Reviewer_Comments_Revision_2 [file giz112_response_to_reviewer_comments_revision_2.pdf]

Author's Response To Reviewer Comments

Close

Dear editor,
Thank you very much for your kindly reminder letter and for the reviewers' comments concerning our manuscript entitled "Hybrid de novo genome assembly of Chinese chestnut (Castanea mollissima)" (GIGA-D-18-00448).
The comments were all valuable and very helpful for us to improve our manuscript. We have studied the comments carefully and made corrections accordingly. Attached please find the revised version, which we would like to submit for your kind consideration.
As the suggestion, we have revised the manuscript carefully and several native speakers also polished and proofread the manuscript again. We have also carefully checked and corrected all values and units in the manuscript again.
We would like to express our appreciation to you and the reviewers for your comments on our paper. We look forward to hearing from you.

Thank you and best regards.

Yours sincerely,

Ling Qin

Ling Qin, Prof.
Beijing University of Agriculture, Beijing, 102206, China
E-mail: qinlingbac@126.com

Response to the Reviewer:

Reviewer #2: While with the availability of all scripts and important intermediate files the concerns with respecit to reproducibility have been adequately addressed, language is still a problem. The authors are encouraged to have their manuscript proofread by a native English speaker.

Answer: Thank you for the suggestion. The manuscript has been revised carefully by our coauthor Professor Ton Bisseling in Wageningen University and also been polished by the senior editor of the AJE company (American Journal Experts, https://secure.aje.com, the contract #6G58F9VV) and finally been proofread by Professor Jocelyn Rose, from School of Integrative Plant Science, Cornell University.

In Supplementary Table 1 the authors give the scaffold N50 of Castanea mollissima of Stanton at al as 2.75 Mb, but the longest scaffold as 3.58 kb. All values and units should be carefully checked.

Answer: Thank you for the suggestion. We have carefully checked and corrected all values and units in the manuscript again.

Close
