# Supplementary material for: Hybrid de novo genome assembly of Chinese chestnut (Castanea mollissima)
Source: Gigascience. 2019 Sep 12;8(9):giz112. doi: 10.1093/gigascience/giz112 (PMC6741814; doi:10.1093/gigascience/giz112)
Supplement: giz112_Reviewer_1_Report_Original_Submission — Jean-Marc Aury -- 12/14/2018 Reviewed [file giz112_reviewer_1_report_original_submission.pdf]

Reviewer Report

Title: Hybrid de novo genome assembly of Chinese chestnut (Castanea mollissima)

Version: Original Submission **Date:** 12/14/2018

Reviewer name: Jean-Marc Aury

Reviewer Comments to Author:

The article describes the sequencing, assembly and annotation of the Chinese chestnut tree (Castanea mollisima) as well as some analysis based on gene families. My main concern about the manuscript is related to the availability of the sequencing data. Indeed the genomic and transcriptomic data are not available through SRA portal. On the other hand, the comparative analyses are not of equal quality. For example, the functional enrichment of specific gene families has not been inspected when compared to the TAGs. In addition, a few points need to be addressed.

Sampling and Sequencing:

- Line 31: Genome DNA instead of Genomic DNA.
- Line 39: approximately 54 Gb, where Table S1 reports 39 Gb.
- Line 49: In total 80 Gb, where Table S1 reports 69 Gb.

Genome size and heterozygosity estimation:

- The authors report a heterozygosity rate of approximately 0.17%, I think it is of interest to compare this rate with other trees, like beech or oak.

Genome assembly and annotation:

- The authors describe the polishing step made using Quiver, but it is unclear how the several (how many?) rounds of iterative error correction, with short reads, were performed.
- The annotation process is not sufficiently described. The authors should describe which databases and which tools were used, and they should describe the transcriptomic dataset.

Quality assessment:

- line 28: genetic regions instead of genic regions.

Gene family expansion and contraction

- A total of 956 and 471 gene families were found to be specific to Chinese chestnut and oak. However on Figure 2a that refers to gene families, the numbers are different. The authors should clarify the text and/or the legend of Figure 2.

Tandemly arrayed genes

- The authors claim that "TAGs are gene clusters created by tandem duplication, and TAGs represent a large proportion of the genes in a genome". Authors should add citations to support this sentence.
- The authors suggest that an abundance of TAGs is a major feature if the genome, but they should compare to other species, such as the oak genome which contains a high proportion of TAGs (as shown in Plomion et al, 2018).

Level of Interest

Please indicate how interesting you found the manuscript: Choose an item.

Quality of Written English

Please indicate the quality of language in the manuscript: Choose an item.

Declaration of Competing Interests

Please complete a declaration of competing interests, considering the following questions:

- Have you in the past five years received reimbursements, fees, funding, or salary from an organisation that may in any way gain or lose financially from the publication of this manuscript, either now or in the future?
- Do you hold any stocks or shares in an organisation that may in any way gain or lose financially from the publication of this manuscript, either now or in the future?
- Do you hold or are you currently applying for any patents relating to the content of the manuscript?
- Have you received reimbursements, fees, funding, or salary from an organization that holds or has applied for patents relating to the content of the manuscript?
- Do you have any other financial competing interests?
- Do you have any non-financial competing interests in relation to this paper?

If you can answer no to all of the above, write 'I declare that I have no competing interests' below. If your reply is yes to any, please give details below.

I declare that I have no competing interests.

I agree to the open peer review policy of the journal. I understand that my name will be included on my report to the authors and, if the manuscript is accepted for publication, my named report including any attachments I upload will be posted on the website along with the authors' responses. I agree for my report to be made available under an Open Access Creative Commons CC-BY license (http://creativecommons.org/licenses/by/4.0/). I understand that any comments which I do not wish to be included in my named report can be included as confidential comments to the editors, which will not be published.

Choose an item.

To further support our reviewers, we have joined with Publons, where you can gain additional credit to further highlight your hard work (see: https://publons.com/journal/530/gigascience). On publication of this paper, your review will be automatically added to Publons, you can then choose whether or not to claim your Publons credit. I understand this statement.

Yes Choose an item.
