# Supplementary material for: Hybrid de novo genome assembly of Chinese chestnut (Castanea mollissima)
Source: Gigascience. 2019 Sep 12;8(9):giz112. doi: 10.1093/gigascience/giz112 (PMC6741814; doi:10.1093/gigascience/giz112)
Supplement: giz112_Reviewer_2_Report_Original_Submission — Marco Thines -- 1/14/2019 Reviewed [file giz112_reviewer_2_report_original_submission.pdf]

Reviewer Report

Title: Hybrid de novo genome assembly of Chinese chestnut (Castanea mollissima)

Version: Original Submission **Date:** 1/14/2019

Reviewer name: Marco Thines

Reviewer Comments to Author:

First of all, I congratulate the authors on there effort and the valuable results obtained. The genome seems mostly well asssembled, but there are some issues as outlined below that should be addressed. The English is of some concern. There are only few grammar and spelling mistakes, but there are many sentences that are phrased in an odd manner, sometimes there is also highly unusual word usage. The authors are thus strongly encouraged to have their manuscrot proofread by a native English speaker.
Title. The title should mention that a hybrid approach was used.
Page 5
Line 24. Castanea sativa has dominant role as forest tree only in few areas of Europe (e.g. some parts of northern Italy and southern France).
Lines 42-44. A citation should be given for this claim.
Lines 51-57. A citation should be given for this claim.
Page 6
Line 9. The available resources should be cited here, e.g. the genome published previously.
Line 28. the sequenced indivual(s) should be described in more detail and the sampling date should be given.
Genome assembly. As SMARTdenovo does not have a correction step, all data should have been corrected before assembly. At 100x, it might have been useful doing self-correction rather than Illumina-based filtering (first).
Page 7
Lines 46-49. More detail neeeds to be provided here. From the information given, it is unclear how gene predictions were actually done.
Line 51. The output from the evidence modeler could be checked against transcripts to vailidate exon/intron boundaries.
Page 8
Line 28. Most?
Page 9
Gene family expansion and contraction / phylogenetic analysis. For both of these analyses, the genome of Fagus sylvatica should be included as well as an important reference point in Fagaceae. In the Venn diagram, it could replace Malus domestica, which is largely unrelated to chestnut and oak.
Line 48. RAxML is a program not a method. It should be mentioned, which version and which substitution model were used, as well as the amount of bootstrap replicated done.
Line 57. It should be mentioned where these divergence times are coming from. the authors could also do their own estimations using a dating software.

Page 10
Tandemly arranged genes. This should be specifically compared to Quercus, Fagus and a species outside Fagaceae, such as walnut and poplar, to investigate, how special this trait is.

Level of Interest

Please indicate how interesting you found the manuscript: Choose an item.

Quality of Written English

Please indicate the quality of language in the manuscript: Choose an item.

Declaration of Competing Interests

Please complete a declaration of competing interests, considering the following questions:

- Have you in the past five years received reimbursements, fees, funding, or salary from an organisation that may in any way gain or lose financially from the publication of this manuscript, either now or in the future?
- Do you hold any stocks or shares in an organisation that may in any way gain or lose financially from the publication of this manuscript, either now or in the future?
- Do you hold or are you currently applying for any patents relating to the content of the manuscript?
- Have you received reimbursements, fees, funding, or salary from an organization that holds or has applied for patents relating to the content of the manuscript?
- Do you have any other financial competing interests?
- Do you have any non-financial competing interests in relation to this paper?

If you can answer no to all of the above, write 'I declare that I have no competing interests' below. If your reply is yes to any, please give details below.

I declare that I have no competing interests.

I agree to the open peer review policy of the journal. I understand that my name will be included on my report to the authors and, if the manuscript is accepted for publication, my named report including any attachments I upload will be posted on the website along with the authors' responses. I agree for my report to be made available under an Open Access Creative Commons CC-BY license (http://creativecommons.org/licenses/by/4.0/). I understand that any comments which I do not wish to be included in my named report can be included as confidential comments to the editors, which will not be published.

Choose an item.

To further support our reviewers, we have joined with Publons, where you can gain additional credit to further highlight your hard work (see: https://publons.com/journal/530/gigascience). On publication of this paper, your review will be automatically added to Publons, you can then choose whether or not to claim your Publons credit. I understand this statement.

Yes Choose an item.
