# Supplementary material for: Hybrid de novo genome assembly of Chinese chestnut (Castanea mollissima)
Source: Gigascience. 2019 Sep 12;8(9):giz112. doi: 10.1093/gigascience/giz112 (PMC6741814; doi:10.1093/gigascience/giz112)
Supplement: giz112_Supplemental_Figures_and_Tables [file giz112_supplemental_figures_and_tables.zip › Figure (S1-S5).doc]

Supplementary figures:

Figure S1

Figure S1 K-mer distribution of *C. mollissima*.

The frequency of each k-mer in raw sequencing reads was calculated for k=17.

Figure S2

Figure S2 GO term analysis for genes in *C. mollissima*

Figure S3

Figure S3. Analysis of the expanded and contracted gene families in *C. mollissima*.

The estimated numbers of orthologous groups in the common ancestral species are shown on the internal nodes. The numbers of orthologous groups that expanded or contracted in each lineage after speciation are shown on the corresponding branch, with ‘+’ referring to expansion and ‘-’ referring to contraction.

Figure S4

Figure S4: Tandemly arrayed gene (TAG) numbers in one cluster in *C. mollissima*

Figure S5

Figure S5: GO enrichment of genes from the TAGs in *C. mollissima*
