# Supplementary material for: Hybrid de novo genome assembly of Chinese chestnut (Castanea mollissima)
Source: Gigascience. 2019 Sep 12;8(9):giz112. doi: 10.1093/gigascience/giz112 (PMC6741814; doi:10.1093/gigascience/giz112)
Supplement: giz112_Supplemental_Figures_and_Tables [file giz112_supplemental_figures_and_tables.zip › Revised protocols.doc]

***Materials and sequencing***

A mature, healthy tree of wild *Castanea mollissima* was chosen from the Zhangcunping national forest reserve (31°16’49.25” N, 111°08’25.40” E, 1261 m altitude) of the city of Yichang in Hubei Province, China. The individual measured ~ 12 m in height, and its trunk was ~10 cm in diameter (at breast height). Fresh leaves were collected in 18 June, 2017. The samples were immediately frozen in liquid nitrogen and then stored at -80℃. The genomic DNA was extracted using the DNeasy Plant Mini Kit (Qiagen, Hilden, Germany) and used for sequencing. The DNA was sheared by Covaris S2 system (Covaris, USA) for short-insert paired-end (PE) library construction. The shearing conditions were as the following: the number of cycles is 2 and shearing time is 40 seconds per cycle. Genomic DNA was sheared using a sonication device for short-insert paired-end (PE) library construction. Short-insert libraries with a size of 500 bp were constructed according to the instructions described in the Illumina Library Preparation Kit (Illumina, CA, USA). All libraries were sequenced on an Illumina HiSeq 2500 sequencer with PE 2×150 bp protocol. Total raw reads were subsequently trimmed for quality using Trimmomatic (version 0.35). Illumina sequence adaptors were removed, low quality bases from the start or end of raw reads were trimmed, and reads were scanned using a 4 bp sliding window and trimmed when the average quality per base dropped below 15. The clean data obtained from this process were used for subsequent analyses. For PacBio library construction, the genomic DNA of *C. mollissima* was sheared to ~20 kb, and short fragments below the size of 7 kb were filtered using BluePippin (Sage Science, MA, USA). Filtered DNA was then converted into the proprietary SMRTbell library using the PacBio DNA Template Preparation Kit (Pacific Biosciences, CA, USA).

***Genome size estimation***

The k-mer frequency distribution, also known as the k-mer spectrum, is widely used for the estimation of genome size [1,2]. We adapted a method called Jellyfish [3], which is based on the k-mer distribution, to estimate the genome size with high quality reads from short-insert-size libraries (500 bp). We obtained a k-mer depth distribution from the Jellyfish analysis and clearly observed the peak depth from the distribution data. To obtain the estimated size of the genome, the following formula was applied: genome size = total_k-mer_num/k-mer_depth, where total_k-mer_num is the total number of k-mers from all reads, and k-mer_depth is the peak depth.

***Genome assembly***

SMARTdenovo software (https://github.com/ruanjue/smartdenovo) was applied to assemble to all of the subreads from PacBio sequencing (The default values for all parameters were used except for -J, which had a value of 4000). -J was used to filter out subreads that did not satisfy the length threshold. The assembly sequence was then polished using Quiver (SMRT Analysis version 2.3.0) with default parameters. To increase the accuracy of the assembly genome, six rounds of iterative error correction were performed using the Illumina clean data above.

***Quality assessment of genome assembly***

To evaluate the quality of the genome assembly, we mapped partial reads from the short-insert-size library back to the scaffolds using Bowtie2 with the following parameters: --rdg 3,1 --rfg 3,1 --gbar 2. A total of 99.1% of the reads could be mapped back to the current assembly. To evaluate the completeness of the genome assembly in genic regions, we analyzed conserved core genes by running the BUSCO software [4] on the assembly of the *C. mollissima* genome.

## *Alignment with physical map*

The BAC end sequences from physical map (version 4) [5] were aligned to our genome sequences by blastn. The match criteria are at least 90% coverage and 90% sequence similarity. The matches were verified empirically by manually examining the FASTA search results.

***Repeat annotation and mask***

The *de novo* identification and masking of repeats were performed by both RepeatModeler and RepeatMasker [6]. To ensure the integrity of genes in the subsequent analyses, low complexity or simple repeats were not masked because some of these repeats could be within genes.

***Gene prediction and annotation***

Protein-coding region identification and gene prediction were performed via a combination of homology-based prediction, *de novo* prediction, and transcriptome-based prediction methods. Proteins from several plant species, namely, *Vitis* *vinifera*, *Prunus* *persica*, *Populus* *trichocarpa*, *Oryza* *sativa*, *Medicago* *truncatula*, *Glycine* *max*, *Citrus* *clementina*, *Theobroma* *cacao*, *Pyrus bretschneideri*, were downloaded from NCBI. Protein sequences were mapped against the *C. mollissima* with Exonerate (version 2.47.3) [7]. The BLAST hits were used to predict the exact gene structures of the corresponding genome regions. Augustus ab initio prediciton software (version 3.2.2) was used to predict coding regions in the repeat-masked genome. For transcriptome-based prediction, transcriptome data were generated from mixed samples from flowers, buds, leaves, nuts and roots on the Illumina HiSeq 2500 platform and mapped against the genome assembly using TopHhat (version 2.1.1). Cufflinks (version 2.2.1) was then used to link the transcripts from the Tophat results to gene models. EVidenceModeler (EVM version 1.1.1) was used to integrate all gene models from the above three methods into a nonredundant gene set.

The obtained gene set was functionally annotated using BLASTP with an E-value of 1e^-5^ against the NCBI-NR, Swiss-Prot, and euKaryotic Orthologous Groups (KOG) databases. To annotate gene functions, protein domains were mapped into the InterPro and Pfam databases using InterProScan [8] and HMMER [9]. The potential gene pathways were derived via gene mapping against the Kyoto Encyclopedia of Genes and Genomes (KEGG) database. Gene Ontology (GO) terms for genes were extracted from the InterProScan or Pfam results.

## *Gene family analysis*

To understand the evolutionary relationships of *C. mollissima* with other plants, we performed a systematic comparison of genes among different species. The full protein-coding genes of 9 genomes, namely, *O.* *sativa* [10], *Malus* *domestica* [11], *P.* *trichocarpa* [12], *P.* *persica* [13], *C.* *mollissima*, *Quercus* *robur* [14], *Fagus* *sylvatica* [15] *Juglans* *regia* [16] and *V.* *vinifera* [17] were used for the comparison. Gene gains and losses are among the primary contributors to functional changes. To obtain greater insights into the evolutionary dynamics of the genes, the expanded and contracted gene orthologue clusters of these 8 species were determined. We used CAFE software (version 3.0) [18] for the computational analysis of gene family evolution and then defined the expansion and contraction of clusters by determining the cluster size difference between each of the current species and ancestor species and comparing these values. The extinction and evolution of gene families were processed by CAFE using the random birth and death process model to identify gene gains and losses along lineages of the RAxML tree. The expanded and contracted gene families (in comparison to ancestors) of all species were compared with those of *C. mollissima* to identify gene families that were only expanded or contracted in the Chinese chestnut*.*

## *Phylogenetic tree construction*

Peptide sequences were clustered by the OrthoMCL Markov clustering program. The peptide sequences were also searched against the NR database by an all-versus-all BLASTP search with a threshold value of E ≤1e^-5^ and then clustered by MCL with an inflation value of 1.5. A total of 637 single-copy orthologous genes were clustered among the 8 genomes. Ortholog alignments were produced using MUSCLE (version 3.6) [19] and then concatenated into a single multiple sequence alignments by an in-house Perl script. A maximum likelihood genome-wide phylogeny was reconstructed using RAxML software (version 8.0.0; substitution model PROTGAMMAJTT, bootstrap value 100) [20].

## *Divergence time estimation*

The molecular clocks and divergence times were estimated by combining the r8s and RAxML programs. The maximum likelihood phylogeny and branch length data were obtained by RAxML with 1000 bootstrap replicates. A fossil-derived timescale and evolutionary history were obtained from TIMETREE (http://timetree.org/). Confidence intervals for r8s could not be obtained due to the lack of convergence of the subsamples.

***Long terminal repeat (LTR) insertion***

Complete LTR-RTs were identified using a combination of *de novo* searches and manual inspection by the LTR_Finder programs. LTR sequences were aligned using the program MUSCLE (version 3.6) [19]. The nucleotide distance for each of the complete LTR elements was calculated with the molecular paleontology approach described by SanMiguel et al [21].

## *Tandemly arrayed genes*

To find TAGs, we clustered genes into putative gene families using OrthoMCL with default parameters. TAGs were found by an in-house script that duplicated genes were separated by less than 10 spacers.

***Raw data quality control***

The raw data have been filtered and trimmed. Illumina data quality control settings are as follows: SLIDINGWINDOW: 4: 15 MINLEN:50 using software as Trimmomatic [22]. Pacbio data quality control standard was RQ>0.75 and the minimum subreads length was 500 bp using software SMRT Link 6.0.

***Check potentially contaminant***

1000 subreads sequences were randomly selected to search against the NT database using BLAST (threshold settings -F F -e 1e-5). If a subread showed the highest alignment with sequences from non-plant species, it is identified as contaminant.

## References

1. Li M, Tian S, Jin L, et al. Genomic analyses identify distinct patterns of selection in domesticated pigs and Tibetan wild boars. Nature genetics 2013; **45** (12): 1431-1438. <https://doi.org/10.1038/ng.2811.>

2. Zhang T, Hu Y, Jiang W, et al. Sequencing of allotetraploid cotton (*Gossypium hirsutum* L. acc. TM-1) provides a resource for fiber improvement. Nature Biotechnology 2015; **33** (5): 531-537. https://doi.org/10.1038/nbt.3207.

3. Marçais G, Kingsford C. A fast, lock-free approach for efficient parallel counting of occurrences of *k*-mers. Bioinformatics 2011; **27** (6): 764-770. <https://doi.org/10.1093/bioinformatics/btr011.>

4. Waterhouse RM, Seppey M, Simão FA, et al. BUSCO applications from quality assessments to gene prediction and phylogenomics. Molecular Biology and Evolution 2018; **35** (3): 543-548. <https://doi.org/10.1093/molbev/msx319.>

5. Fang GC, Blackmon BP, Staton ME, et al. A physical map of the Chinese chestnut (*Castanea mollissima*) genome and its integration with the genetic map. Tree Genetics & Genomes 2013; **9** (2): 525-537. https://doi.org/10.1007/s11295-012-0576-6.

6. Tarailo-Graovac M, Chen NS. Using RepeatMasker to identify repetitive elements in genomic sequences. Current Protocols in Bioinformatics 2009; **25**: 4.10.1-4.10.14. <https://doi.org/10.1002/0471250953.bi0410s25.>

7. Slater GSC and Birney E. Automated generation of heuristics for biological sequence comparison. BMC Bioinformatics 2005; **6**: 31. https://doi:10.1186/1471-2105-6-31.

8. Jones P, Binns D, Chang HY, et al. **InterProScan 5: genome-scale protein function classification.** Bioinformatics 2014; **30** (9): 1236-1240. https://doi.org/10.1093/bioinformatics/btu031.

9. Wheeler TJ, Eddy SR. [nhmmer: DNA homology search with profile HMMs](http://bioinformatics.oxfordjournals.org/content/29/19/2487). Bioinformatics 2013; **29** (19): 2487-2489. <https://doi.org/10.1093/bioinformatics/btt403.>

10. International Rice Genome Sequencing Project. The map-based sequence of the rice genome. Nature 2005; **436** (7052): 793-800. https://doi.org/10.1038/nature03895.

11. Velasco R, Zharkikh A, Affourtit J, et al. The genome of the domesticated apple (*Malus* × *domestica* Borkh.). Nature Genetics 2010; **42** (10): 833-839. https://doi.org/10.1038/ng.654.

12. Tuskan GA, Difazio S, Jansson S, et al. The genome of black cottonwood, *Populus trichocarpa* (Torr. & Gray). Science 2006; **313** (5793): 1596-1604. https://doi.org/10.1126/science.1128691.

13. Verde I, Abbott AG, Scalabrin S, et al. The high-quality draft genome of peach (*Prunus persica*) identifies unique patterns of genetic diversity, domestication and genome evolution. Nature Genetics 2013; **45** (5): 487-494. https://doi.org/10.1038/ng.2586.

14. Plomion C, Aury JM, Amselem J, et al. Oak genome reveals facets of long lifespan. Nature Plants 2018; **4** (7): 440-452. <https://doi.org/10.1038/s41477-018-0172-3.>

15. Mishra B, Gupta DK, Pfenninger M, et al. A reference genome of the European beech (*Fagus sylvatica* L.). GigaScience 2018; **7** (6): 1-8. https://doi.org/10.1093/gigascience/giy063.

16. Martínez-García PJ, Crepeau MW, Puiu D, et al. The walnut (*Juglans regia*) genome sequence reveals diversity in genes coding for the biosynthesis of non-structural polyphenols. The Plant Journal 2016; **87** (5): 507-532. https://doi.org/10.1111/tpj.13207.

17. The French-Italian Public Consortium for Grapevine Genome Characterization. The grapevine genome sequence suggests ancestral hexaploidization in major angiosperm phyla. Nature 2007; **449** (7161): 463-467. <https://doi.org/10.1038/nature06148.>

18. De Bie T, Cristianini N, Demuth J, et al. CAFE: a computational tool for the study of gene family evolution. Bioinformatics 2006; **22** (10): 1269-1271. https://doi.org/10.1093/bioinformatics/btl097.

19. Edgar RC. MUSCLE: multiple sequence alignment with high accuracy and high throughput. Nucleic Acids Research 2004; **32** (5): 1792-1797. <https://doi.org/10.1093/nar/gkh340.>

20. Stamatakis A. RAxML version 8: a tool for phylogenetic analysis and post-analysis of large phylogenies. Bioinformatics 2014; **30** (9): 1312-1313. https://doi.org/10.1093/bioinformatics/btu033.

21. SanMiguel P, Gaut BS, Tikhonov A, et al. The paleontology of intergene retrotransposons of maize. Nature Genetics 1998; **20** (1): 43-45. <https://doi.org/10.1038/1695.>

22. Bolger AM, Lohse M, Usadel B. Trimmomatic: A flexible trimmer for Illumina Sequence Data. Bioinformatics 2014; doi:10.1093/bioinformatics/btu170.
