# Supplementary material for: Hybrid de novo genome assembly of Chinese chestnut (Castanea mollissima)
Source: Gigascience. 2019 Sep 12;8(9):giz112. doi: 10.1093/gigascience/giz112 (PMC6741814; doi:10.1093/gigascience/giz112)
Supplement: giz112_Supplemental_Figures_and_Tables [file giz112_supplemental_figures_and_tables.zip › Tables (S1-S5, S9).doc]

Supplementary tables:

Table S1. Comparison of assembly quality in two genomes of *C. mollissima*

| **Species** | ***C. mollissima***  **(in this study)** | ***C. mollissima* （Staton et al. 2019）** | | | |
| --- | --- | --- | --- | --- | --- |
|  |  | **Assembly 1.1** | **Assembly 2.0** | **Assembly 3.2** | **Assembly 4.0** |
| Total length of contig sequences | 785.53 Mb | 843.3 Mb | 760 Mb | 783.4 Mb | 783.4 Mb |
| Maximum length of contigs | 6.6 Mb | / | 3.58 Kb | 1.1 Mb | 1.1 Mb |
| Counts of contig sequences | 2,707 | 323,611 | 60,546 | 12,684 | 12,684 |
| Counts of scaffold sequences | / | 41,270 | 14,358 | / | / |
| Contig length range | 1 Kb - 6.6 Mb | / | 2 Kb - 3.58 Kb | 2 Kb - 1.1 Mb | 2 Kb - 1.1 Mb |
| Scaffold length range | / | 2 Kb - 429 Kb | 2 Kb - 5.5 Mb | / | / |
| Counts of contig N50 | 235 | / | / | / | / |
| Contig N50 | 944,461 bp | 9,473 bp | / | / | / |
| Scaffold N50 | / | 39.6 Kb | 2.75 Mb | / | / |

Table S2. Statistics of clean data of *C. mollissima* for Illumina and PacBio sequencing.

| **Clean Reads** | **Illumina Sequencing** | | **PacBio Sequencing** | |
| --- | --- | --- | --- | --- |
|  | **Raw reads** | **Clean reads** | **Polymerase reads** | **Subreads (RQ>0.75 length>500)** |
| Size of library | 500 bp | 500 bp | 20 kb | 20 kb |
| Number of reads | 118,123,542*2 | 113,618,248*2 | 4,961,145 | 9,581,541 |
| Average length of reads | 150 bp | 147 bp | 13,971 bp | 7,170 bp |
| Total bases | 35,437,062,600 bp | 33,580,328,092 bp | 69,312,850,332 bp | 68,707,578,456 bp |
| Sequencing depth | ~45.1 X | ~42.7 X | ~639 X | ~87 X |

Table S3. Comparison of genome size and heterozygosity in three species of *C. mollissima*, *Q. robur* and *F. sylvatica*.

|  | **Chinese chestnut**  **(*C. mollissima*)** | **oak**  **(*Q. robur*)** | **European beech**  **(*F. sylvatica*)** |
| --- | --- | --- | --- |
| Genome size | 772 Mb | 1,060 Mb | 611 Mb |
| Heterozygosity | 0.87 % | 1.62 % | 0.63 % |

Heterozygosity rate of oak is from Ramos AM et al., 2018 [22]. In the genome paper of European beech [36], the heterozygosity rate is not mentioned. In order to compare the heterozygosity rate in three species, we download the PRJEB24056 (ERX2326485 and EXR2326486) from NBCI database and estimated the heterozygosity rate is approximately 0.63% based on k-mers method.

Table S4: Statistics of repeat elements for *C. mollissima* assembly using both RepeatModeler and RepeatMasker software

|  | **Number** | **Length (bp)** | **Percentage (%)** |
| --- | --- | --- | --- |
| **SINEs:** | 100 | 6,815 | 0.00 |
| **LINEs:** | 53,947 | 28,492,759 | 3.63 |
| **LINE1** | 35,231 | 23,564,558 | 3.00 |
| **LINE2** | 256 | 50,883 | 0.01 |
| **LTR elements:** | 162,435 | 156,475,746 | 19.92 |
| **ERV_classI** | 613 | 185,817 | 0.02 |
| **DNA elements:** | 113,355 | 42,298,189 | 5.38 |
| **Unclassified:** | 647,126 | 159,487,992 | 20.30 |
| **Total interspersed repeats:** |  | 386,761,501 | 49.24 |
| **Satellites:** | 1,510 | 388,945 | 0.05 |
| **Simple repeats:** | 13,180 | 3,153,925 | 0.40 |
| **Total** |  | 390,304,371 | 49.69 |

**Table S5. Core gene estimation for *C. mollissima* assembly using BUSCO**

| **Feature** | **Number** | **Percentage** (%) |
| --- | --- | --- |
| Complete BUSCOs (C) | 1,392 | 96.70 |
| Complete and single-copy BUSCOs (S) | 1,281 | 89.00 |
| Complete and duplicated BUSCOs (D) | 111 | 7.70 |
| Fragmented BUSCOs (F) | 20 | 1.40 |
| Missing BUSCOs (M) | 28 | 1.90 |
| Total BUSCO groups searched | 1,440 |  |

Table S9 Numbers and proportions of TAGs in *C. mollissima* and other species

|  | ***C. mollissima*** | ***F.***  ***sylvatica*** | ***Q.***  ***robur*** | ***P. trichocarpa*** | ***J.***  ***regia*** | ***M.***  ***domestica*** | ***P.***  ***persica*** |
| --- | --- | --- | --- | --- | --- | --- | --- |
| Gene cluster number | 1,112 | 1,304 | 1,467 | 636 | 696 | 673 | 906 |
| Gene number | 4,198 | 4,989 | 5,099 | 2,263 | 2,054 | 2,008 | 3,027 |
| Percentage (%) | 11.5 | 8.0 | 19.7 | 4.9 | 5.6 | 4.2 | 13.3 |
